# Synthesis of (±)-Angustatin A: Assembly of the Phenanthrene Moiety Despite Increasing Ring Strain

**DOI:** 10.1021/acs.orglett.3c02742

**Published:** 2023-09-25

**Authors:** Hoang D. Doan, Christian Rugen, Christopher Golz, Manuel Alcarazo

**Affiliations:** Institut für Organische und Biomolekulare Chemie, Georg-August-Universität Göttingen, 37077 Göttingen, Germany

## Abstract

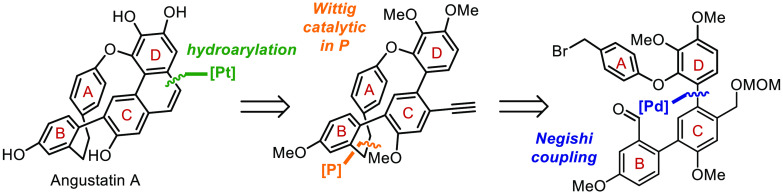

The synthesis of (±)-angustatin A, a phenanthrene-containing cyclophane that possesses conformational chirality, is reported. Key steps include a Pd-catalyzed Negishi coupling to assemble the necessary terphenyl intermediate, its closure into a 14-membered macrocycle via a catalytic-in-phosphine Wittig olefination, and finally a Pt-catalyzed alkyne hydroarylation, which is able to assemble the phenanthrene unit despite the thermodynamic cost of significantly bending arene A from the ideal plane.

The cyclophane angustatin A, which has been isolated from the liverwort *Asterella angusta*, displays several intriguing architectural features that makes it an attractive synthetic objective.^1^ It shares with the closely related and more popular target cavicularin a highly strained 14-membered macrocyclic core; however, in contrast to the structure of the latter, in angustatin A the phenanthrene motif remains completely unsaturated.^2,3^ An additional hydroxy-substituent on ring D of angustatin A completes the differences between the two natural products. Although no crystallographic evidence is available to date, angustatin A is expected to contain a boat-like arene moiety (ring A) and probably a significantly twisted phenanthrene unit (Scheme 1). The nonequivalent ^1^H NMR signals from ring A, and their unusual chemical shift, also suggest that as result of its rigid skeleton angustatin A displays conformational chirality;^2,4^ however, no optical rotation value has been reported to date.

**Scheme 1:**
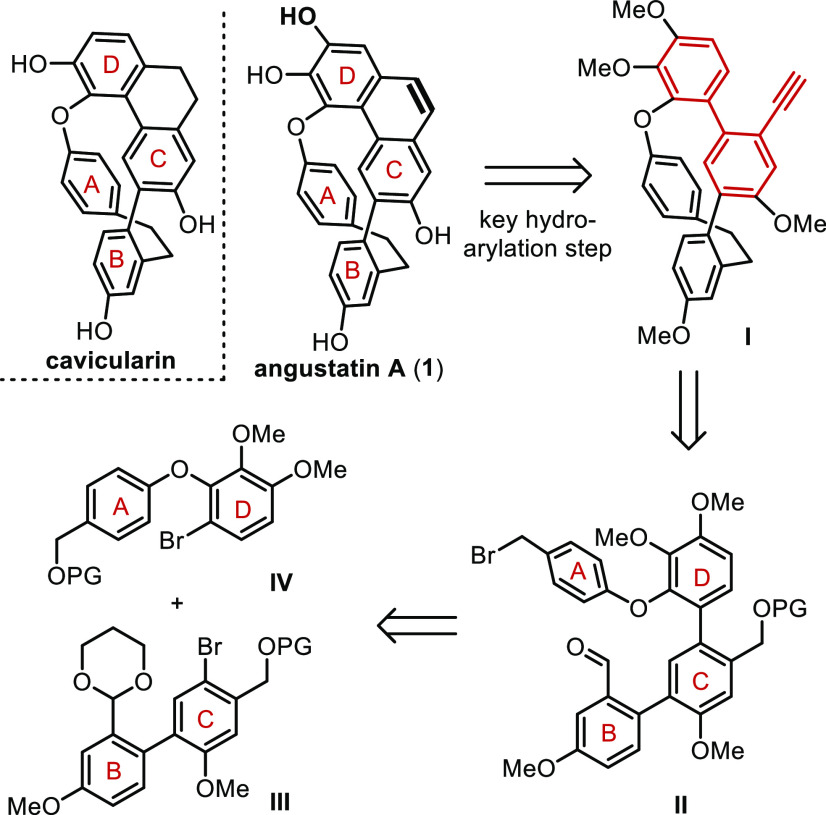
Synthetic Strategy Towards Angustatin A

It is for these unique molecular attributes, and also due to our long-term interest in the synthesis of twisted polyaromatic structures via Au(I)- or Pt(II)-catalyzed alkyne hydroarylation reactions, that we decided to develop a synthetic route toward angustatin A. Specifically, we plan to construct the phenanthrene unit at a late stage of the synthesis.^5^ This will allow a comparative evaluation of the available catalysts in a challenging cyclization event. Note that the transformation of the 2-alkynyl biaryl moiety in **I** (Scheme 1, highlighted in red) into the desired phenanthrene must occur by overcoming the thermodynamic cost of heavily contorting aromatic ring A.

These notions are reflected in our retrosynthetic plan. We initially aimed at the preparation of cyclophane intermediate **I**, in which the phenanthrene skeleton is still not assembled and, consequently, the ring strain of the structure is significantly reduced. The higher flexibility of **I** should also facilitate its closure from **II** via Wittig olefination. The preparation of precursor **II** was envisioned through the Negishi coupling of conveniently substituted biphenyl and diarylether fragments **III** and **IV**, which were respectively prepared in short sequences from simple commercially available materials (Scheme 1).

Hence, the assembly of fragment **IV** started with 2,3-di(methoxy)phenol, which was regioselectively brominated on multigram scale to deliver **2**.^6^ Treatment of this compound with 4-fluorobenzaldehyde delivered ether **3**.^7^ Reduction of the aldehyde unit in **3** to the corresponding benzylic alcohol **4** took place under standard conditions, and final protection with dihydropyrane gave rise to **5** (Scheme 2a).^8^

**Scheme 2:**
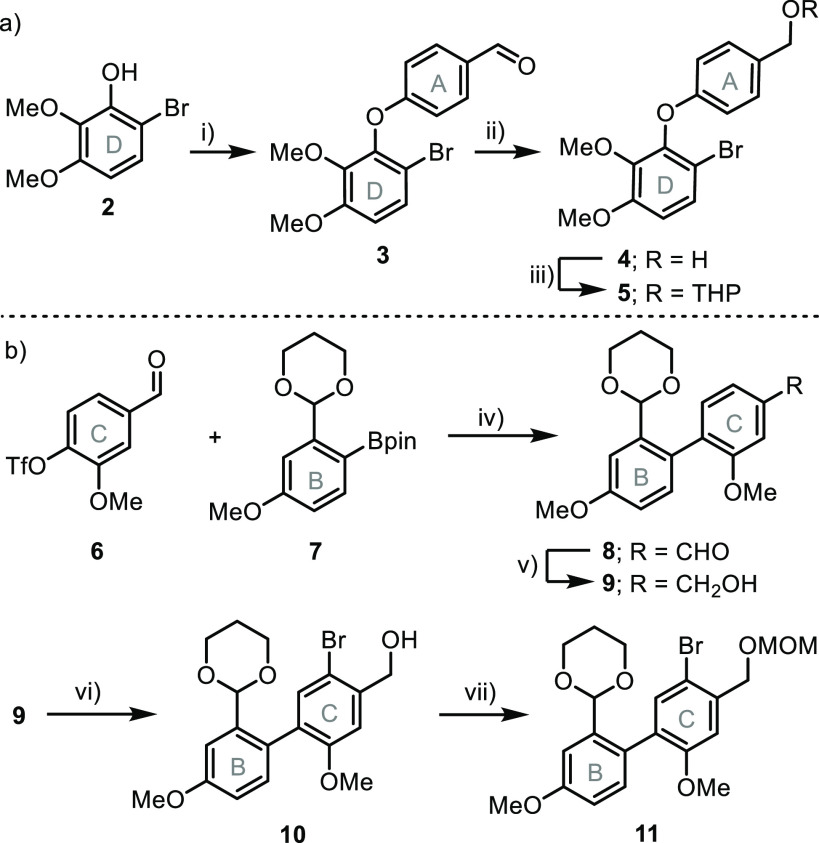
Elaboration of the Synthetic Equivalents of Fragments **III** and **IV** Reagents and conditions: (i) 4-fluorobenzaldehyde, K_2_CO_3_, DMSO, 95 °C, 40%; (ii) NaBH_4_, THF/EtOH, r.t., 84%; (iii) DHP, PPTS (10 mol %), CH_2_Cl_2_, 92%; (iv) Pd(PPh_3_)_4_ (13 mol %), Na_2_CO_3_, EtOH/toluene 105 °C, 80%; (v) NaBH_4_, THF/EtOH, 0 °C, 97%; vi) NBS, THF, −78 °C, 75%; (vii) MOMCl, DIPEA, CH_2_Cl_2_, 0 °C, 99%.

The equivalent of fragment **III** was prepared in a straightforward sequence via the Suzuki coupling between already described triflate **6** and pinacol borate **7** to deliver biaryl **8**; both precursors are efficiently prepared in multigram scale from vanillin and *m*-anisaldehyde, respectively.^9,10^ The reduction of the aldehyde functionality in **8** to the corresponding alcohol **9** is required at this stage in order to introduce the bromine substituent selectively at 4-position of ring C in **10**.^11^ Final MOM protection of the benzylic alcohol affords **11** in a 58% yield after 4 steps (Scheme 2b).

The Negishi reaction between **5** and **11** delivered **12** albeit in a moderate yield; this is probably a consequence of the steric bulk of the fragments to be coupled, which are both *o*-substituted. Actually, the ^1^H NMR spectrum of **12** depicts many broad signals, indicating that rotation between rings B–C and C–D are hindered (see the Supporting Information). In the subsequent steps, compound **14** was generated to enable the desired macrocyclization through an intramolecular Wittig olefination.^12^ This was done by acid removal of the two acetal moieties in **12** and transformation of the benzylic alcohol unit in **13** into a bromide via Appel reaction. At that stage, addition of PPh_3_ to **14** generated the corresponding phosphonium salt, which upon treatment with NaOMe delivered the desired macrocycle **15** in a 38% yield (two steps). ^1^H NMR analysis of the crude reaction mixture indicated that under these experimental conditions the (*E*)-stereoisomer is exclusively obtained (^3^*J*_(R*H*C=C*H*R′)_ = 16.9 Hz).^13^ The assembly of **15** was also attempted via the alternative, and more direct, reaction of **13** with [Ph_3_PH]Br followed by basic treatment; however, this broadly used and more direct protocol was lower yielding for that specific substrate.^14^ HPLC analysis of a sample of **15** on chiral stationary phase (Daicel IA-3 SFC) followed by measurement of the circular dichroism (CD) spectrum of the eluates showed that this cyclophane already exists as a mixture of two configurationally stable enantiomers.

This result suggests that if a catalytic-in-phosphine Wittig reaction could be made operative for the ring closing step, then an enantioselective synthesis of angustatin A might be developed. Thus, we initially turned our attention to **22**, a 9-phosphabicyclo[4.2.1]nonane derivative that has already demonstrated its utility as a catalyst for Wittig reactions when diphenylsilane is used as a reductant.^15^ To our delight, the macroolefination took place under these conditions in moderate yield (60%); yet, instead of **15** the product obtained was **16**, in which the regio- and diastereoselective hydrosilylation of the initially formed double bond had occurred. Actually, treatment of **15** with H_2_SiPh_2_ (1.0 equiv) under the reaction conditions applied during the olefination step (toluene, 125 °C) affords a mixture that contains the (*Z*)-isomer of **15** and **16**. Hydrosilylation reactions are often promoted by metal, Lewis acid, or Lewis base catalysts;^16^ we believe, however, that the release of strain in **15** facilitates this spontaneous addition. The unexpected hydrosilylation is inconsequential regarding the total number of steps and final yield, since the treatment of **16** with TBAF quantitatively delivers **17**, and otherwise the double bond in **15** would need to be reduced. When (1*R*,4*R*)-7-phenyl-7-phosphabicyclo[2.2.1] heptane-7-oxide **23a** was employed as the catalyst under otherwise identical conditions, the macroolefination still worked (42% yield) but compound **16** was obtained with low enantioselectivity (40% ee, determined after desilylation in **17**). The more sterically demanding catalyst **23b** is characterized by reduced reactivity and required higher operating temperatures; unfortunately, when the reaction conditions were forced (180 °C), only decomposition was observed. It is for this reason that the following steps of the synthesis, namely, MOM-deprotection of **17** to alcohol **18** and the homologation of this moiety into an alkyne in **21**, were continued with racemic material (Scheme 3).

**Scheme 3:**
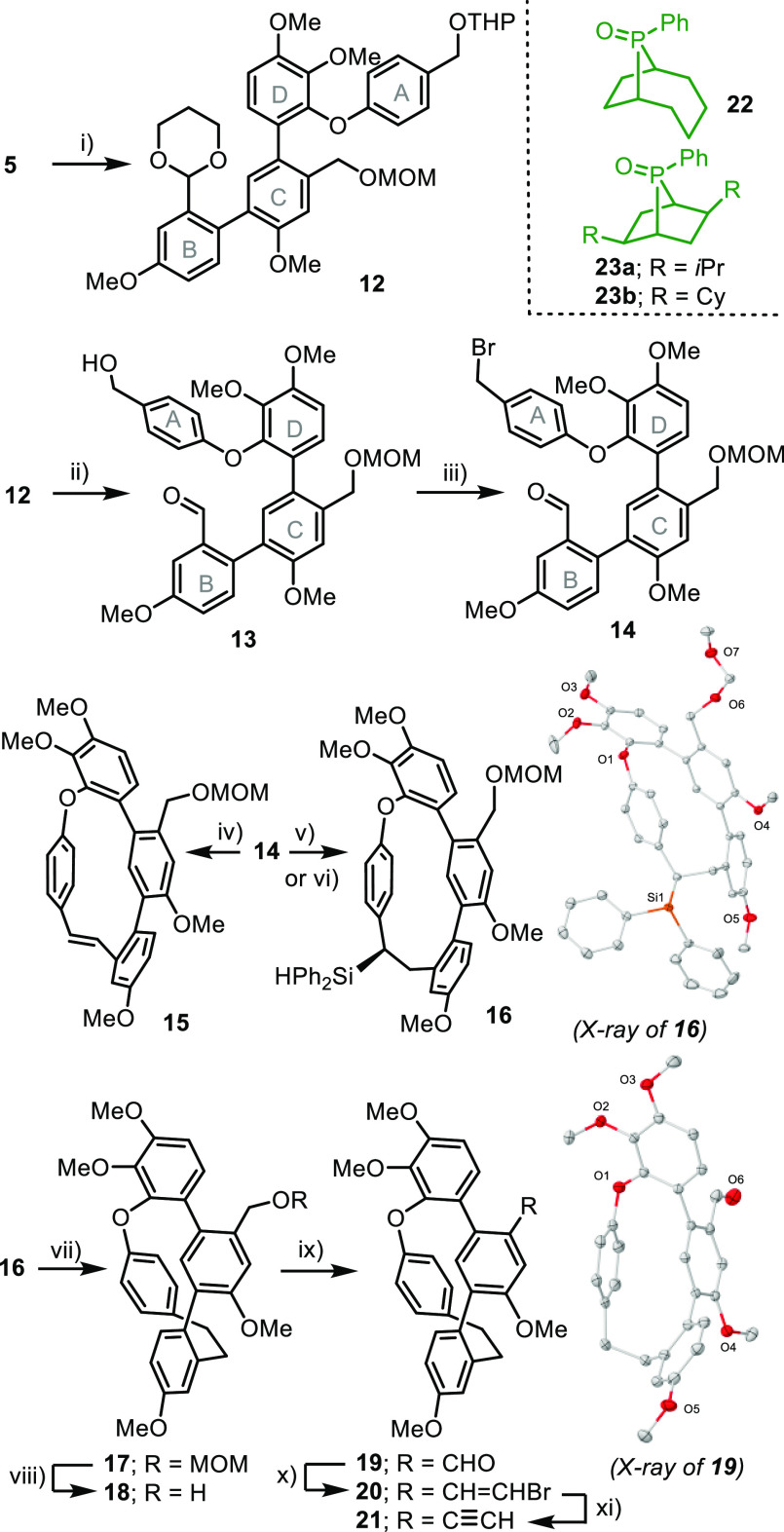
Assembling the Macrocycle via the Wittig Reaction Reagents and conditions: (i) *n*BuLi, THF, −78 °C, 1 h, then ZnBr_2_, r.t., 2 h, and then **11** (1.0 equiv), Pd(PPh_3_)_4_ (5 mol %), 100 °C, 61%; (ii) HCl in EtOH, r.t., 84%; (iii) CBr_4_ (1.1 equiv), PPh_3_ (1.1 equiv), 0 °C, 80%; (iv) PPh_3_ (1.1 equiv), toluene, 110 °C, and then NaOMe (3.0 equiv), r.t., CH_2_Cl_2_, 6 h, 38%, (two steps); (v) **22** (30 mol %), KO*t*Bu (2.0 equiv), Ph_2_SiH_2_ (1.2 equiv), toluene, 110 °C, 3 d, 60%; (vi) **23a** (30 mol %), KO*t*Bu (2.0 equiv), Ph_2_SiH_2_ (1.2 equiv), toluene, 125 °C, 3 d, 42% or **23b** (30 mol %), KO*t*Bu (2.0 equiv), Ph_2_SiH_2_ (1.2 equiv), toluene, 180 °C, 3 d, decomp.; (vii) TBAF (1.5 equiv), THF, r.t., quant.; (viii) HCl, acetone, r.t., 74%; (ix) DMP (1.5 equiv), K_2_CO_3_ (3 equiv), r.t., 98%; (x) [Ph_3_PCH_2_Br]Br (1.1 equiv), KO*t*Bu (1.3 equiv), THF, −78 °C, 73%; (xi) Et_2_NH (2.0 equiv), *n*BuLi (2.0 equiv), −78 °C, then **20** (1.0 equiv), r.t., 12 h., 99%. Molecular structures of **16** and **19** are in the solid state. Anisotropic displacements are shown at the 50% probability level. Hydrogen atoms and solvent molecules were removed for clarity.

At that point, the stage was set for the key 6-*endo*-dig hydroarylation step. Two typical catalytic conditions for this reaction were initially chosen, namely, PtCl_2_ in Cl(CH_2_)_2_Cl at 80 °C^17^ and Ph_3_PAuCl/AgSbF_6_ in CH_2_Cl_2_ at r.t.^18^ The first one proved to be moderately effective, furnishing the desired phenanthrene **24** in a 33% yield, whereas the use of the Au-based catalytic system resulted in the formation of just traces of the desired product. Up to a certain level, increasing the π-acceptor character of the ancillary ligand coordinated to Au seems to be beneficial. For example, (PhO)_3_PAuCl and **26**, bearing an α-pyridiniophosphine ligand, delivered **24** in 31% and 61% yields, respectively;^19^ however, the employment of Au-precatalysts based on even more electron-poor α-cationic phosphines resulted in fast catalyst decomposition after activation with AgSbF_6_.^20^ Interestingly, it was the Pt precatalyst **25** the one that more effectively promoted the cyclization in terms of isolated yields.^21^ We ascribe this remarkable result to several synergic effects: (i) the enhanced electrophilicity at Pt center, which derives from the use of a polyfluoronated and cationic ancillary phosphine; (ii) the low appetence of the Pt(II) atom to be reduced by the substrate; and (iii) the robustness of **25** at elevated temperatures (Scheme 4a and b).^22^

**Scheme 4:**
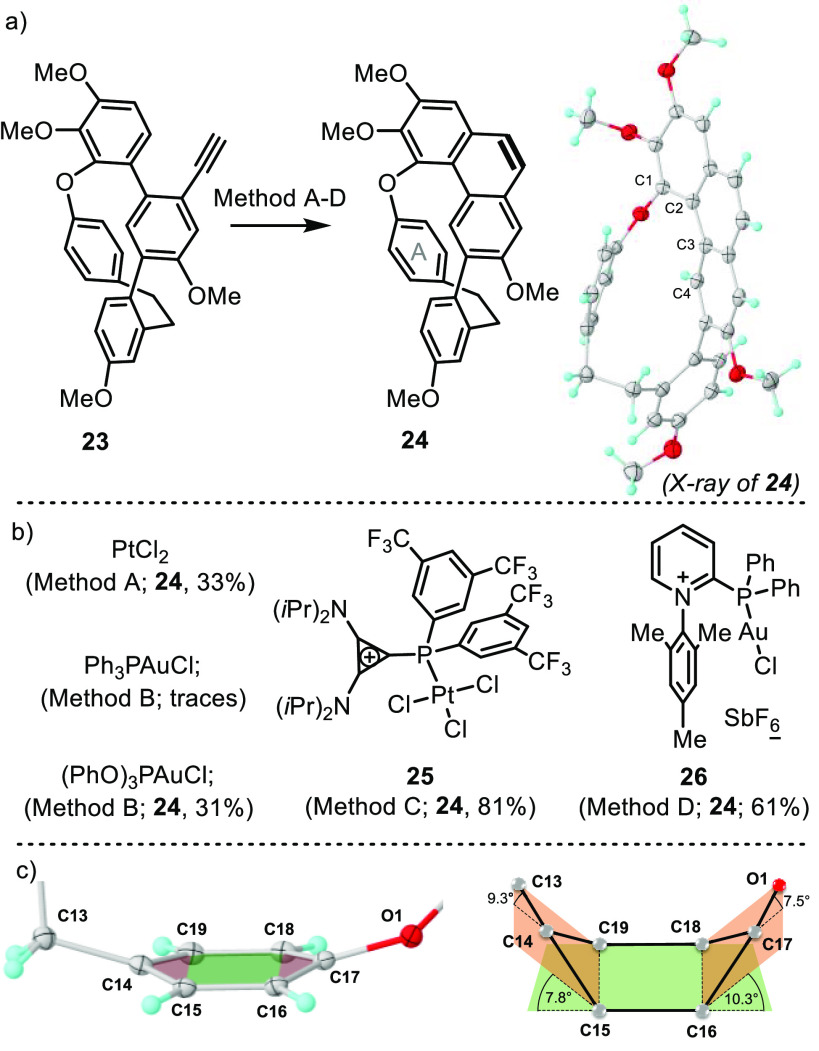
Construction of the Phenanthrene Core Reagents and conditions: method A, PtCl_2_ (5 mol %), dichloroethane, 80 °C, 12h; method B, Au catalyst (5 mol %), AgSbF_6_ (5 mol %), rt, 12 h; method C, **25** (5 mol %), AgSbF_6_ (5 mol %), toluene, 80 °C, 1.5 h; method D, **26** (5 mol %), AgSbF_6_ (5 mol %), rt, 1 h. Molecular structure of **22** in the solid state. Anisotropic displacements are shown at the 50% probability level. Solvent molecules were removed for clarity.

X-ray analysis of **24** confirmed that the angustatin A core structure had been assembled despite the need to bend arene A 17° from ideal planarity and the imposition of a torsion to the phenanthrene unit (Φ_C1–C2–C3–C4_ = 13.7°) to achieve this goal (Scheme 4c). This situation seems to be even more strained than that in the case of cavicularin, where the same analysis only yields a mean bending of 14° in ring A.^3c^ The higher torsional flexibility of the 9,10-dihydrophenanthrene unit in cavicularin when compared to the phenanthrene in **24** probably accounts for this partial strain relaxation.

Finally, removal of the methyl protecting groups in **24** with BBr_3_ (10.0 equiv., r.t.) allowed the clean production and isolation of angustatin A (**1**) (Scheme 5a). All spectral data (^1^H and ^13^C NMR, HRMS) from the synthetic material were consistent to those reported for the natural product.^1^ Crystals of **1** could not be grown; yet, the addition of 1 equiv of AcOK to a MeOH solution of **1** readily induced the crystallization of the angustatin A·KOAc complex, in which a potassium cation coordinates the catechol moiety of **1** (Scheme 5b). Finally, the separation of the constitutional enantiomers of **1** was carried out via semipreparative HPLC with a chiral stationary phase (Daicel IA-3 SFC). The CD spectra of the samples were, as expected, mirror images (Scheme 5c), and the optical rotation of both optically pure enantiomers showed opposite values, [α]_D_^20^ = −210 (*c* 0.13, MeOH, for the second eluted enantiomer); these phenomena definitively proved that **1** displays conformational chirality.

**Scheme 5:**
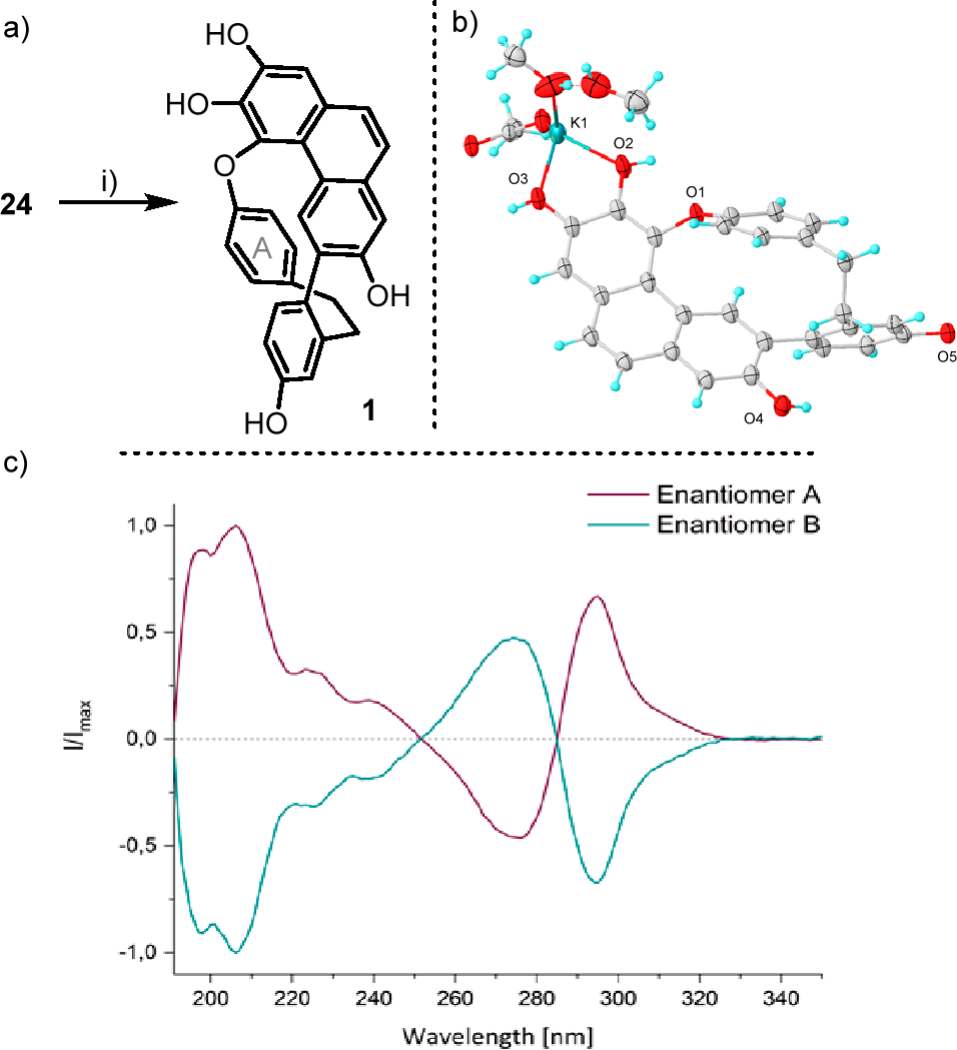
Final Deprotection Reagents and conditions: (i) BBr_3_ (10.0 equiv), CH_2_Cl_2_, r.t., 95%; Molecular structure of **1**·KOAc in the solid state. Anisotropic displacements are shown at the 30% probability level; cocrystallized MeOH molecules are shown. Enantiomer A eluted first (red line), and enantiomer B eluted second (green line)

In conclusion, the first synthesis of angustatin A has been accomplished. Noteworthy features of the route developed are the use of a Wittig reaction catalytic-in-phosphorus to effect the macrocyclization and the late construction of the phenanthrene ring via Pt-catalyzed alkyne hydroarylation. As initially expected, angustatin A displays conformational chirality due to ring strain.

## Data Availability

The Data underlying this study are available in the published article and its Supporting Information.

## Supporting Information Available

General experimental procedures and characterization data, including ^1^H- and ^13^C NMR spectra of new compounds (PDF)
