# Supplementary material for: Synthesis of (±)-Angustatin A: Assembly of the Phenanthrene Moiety Despite Increasing Ring Strain
Source: Org Lett. 2023 Sep 25;25(39):7181–5. doi: 10.1021/acs.orglett.3c02742 (PMC10563161; doi:10.1021/acs.orglett.3c02742)
Supplement: Supplementary file 1 — ol3c02742_si_001.pdf [file ol3c02742_si_001.pdf]

SUPPORTING INFORMATION

SYNTHESIS OF (±)-ANGUSTATIN A: ASSEMBLY OF THE PHENANTHRENE MOIETY DESPITE INCREASING RING STRAIN

Hoang D. Doan, Christian Rugen, Christopher Golz and Manuel Alcarazo

Institut für Organische und Biomolekulare Chemie, Georg August Universität Göttingen, 37077 Göttingen, Germany.

TABLE OF CONTENT

MATERIALS AND METHODS 4

EXPERIMENTAL PROCEDURE 6

SYNTHESIS OF THE FRAGMENT IV 6

- Synthesis of 2 6
- Synthesis of 3 6
- Synthesis of 4 7
- Synthesis of 5 8

SYNTHESIS OF FRAGMENT III 8

- Synthesis of 2-(2-bromo-5-methoxyphenyl)-1,3-dioxane 8
- Synthesis of 7 9
- Synthesis of 6 10
- Synthesis of 8 10
- Synthesis of 9 11
- Synthesis of 10 12
- Synthesis of 11 12

PREPARATION OF FRAGMENT II AND SUBSEQUENT SYNTHESIS 13

- Synthesis of 12 13
- Synthesis of 13 14
- Synthesis of 14 15
- Synthesis of 15 15
- Synthesis of 16 16
- Synthesis of 17 17
- Synthesis of 18 18
- Synthesis of 19 19
- Synthesis of 20 20
- Synthesis of 21 20
- Synthesis of 24 21
- Synthesis of 1 22

$^{1}H/^{13}C$ NMR DATA COMPARISON WITH DATA AVAILABLE 23

SPECTRA 24

- Compound 2 24
- Compound 3 25
- Compound 4 26

Compound 5 27
Compound S04 28
Compound 6 29
Compound 7 30
Compound 8 31
Compound 9 32
Compound 10 33
Compound 11 34
Compound 12 35
Compound 13 36
Compound 14 37
Compound 15 38
Compound 16 39
Compound 17 40
Compound 18 41
Compound 19 42
Compound 20 43
Compound 21 44
Compound 24 45
Compound 1 46

CD MEASUREMENTS 47

HPLC SPECTRA 47

CRYSTALLOGRAPHIC SUPPLEMENT 48

Compound 1◦2MeOH◦KOAc 48
Compound 11 49
Compound 16◦0.5 $CHCl_3$ 50
Compound 19 51
Compound 22 53
Compound 24 54

Materials and Methods

Unless stated otherwise, all reactions were carried out using pre-dried glassware under an inert atmosphere (nitrogen or argon) using standard Schlenk techniques, or in a MBraun UNIlab plus glovebox. After quenching the reaction mixtures were concentrated under reduced pressure by rotary evaporation at 25–40 °C. Purified compounds were further dried under high vacuum. Yields refer to purified and spectroscopically pure compounds.

Solvents: Dry and degassed solvents (THF, dichloromethane, toluene, diethyl ether, pentane, acetonitrile) were obtained from a MBraun Solvent Purification System (MB-SPS-800) or by distillation over the appropriate drying agent and stored under a protective gas atmosphere.

Chromatography: Thin layer chromatography (TLC) was performed using polygram SIL G/UV254 TLC plates from Macherey Nagel and visualized by UV irradiation and/or phosphomolybdic acid or $KMnO_4$ dip. Flash column chromatography was performed using Macherey Nagel 60 (40-63 µm) silica gel.

Starting materials: Commercially available reagents (including **S01**, **S03** and **S05**) were purchased from ABCR, Acros Organics, Alfa Aesar, ChemPur, Sigma-Aldrich, TCL, and Thermo Fischer Scientific and used as received.

NMR: Spectra were recorded on Bruker Avance Neo 600, Avance Neo 400, Avance III HD 400, Avance III 400 or Avance III HD 300 spectrometers. ^1H and ^{13}C chemical shifts (δ) are reported in ppm relative to TMS using the solvent signals as reference in $CDCl_3$ (^1H: 7.26 ppm, ^{13}C: 77.16 ppm) or C_6D_6 (^1H: 7.16 ppm, ^{13}C: 128.1 ppm). Coupling constants (*J*) are given in Hertz (Hz). Data is reported as follows: s = singlet, d = doublet, t = triplet, q = quartet, m = multiplet, br = broad; coupling constants in Hz; integration.

HRMS: Spectra were recorded using Bruker Daltonik maXis Q-TOF (ESI), Bruker Daltonik micrOTOF (ESI), Thermo Scietific LTQ Orbitrap XL (ESI), Thermo Scietific Exactive GC-Orbitrap-MS (EI) or Jeol AccuTOF (EI) instruments. Dimensionless mass-to-charge ratios (*m*/*z*) are given.

IR: Infrared spectra were recorded on a FT/IR-4600 spectrometer and reported in wavenumbers (cm^{-1}) with the intensity of the signals described with vs (very strong), s (strong), m (medium) or w (weak).

Melting point: Melting points were measured with a Büchi M-560 apparatus with a heating rate of 5°C/min.

Circular dichroism: The spectra were conducted on a JASCO J-1500 at 20 °C in MeOH (0.2 mmol). The samples were measured in a quartz sample cell with an optical path length of 0.1 cm.

Chiral HPLC: chiral HPLC measurements were performed using a Shimadzu Prominence-i LC2030C 3D Plus equipment with integrated downstream UV/Vis PDA detector. System control and chromatogram analysis were carried out with LabSolutions software version 5.92. Enantioselective separations were conducted on a Chiralpak® IA-3 (150 mm, i.d. 4.6 mm, particle size 3 µm) or a Chiralpak® IC-3 (150 mm, i.d. 4.6 mm, particle size 3 µm) column, which were bought from Daicel Chiral Technologies. The solvents used (*n*-hexane, *iso*-propanol, ethyl acetate) were purchased from Fisher Scientific or Sigma-Aldrich in HPLC-

grade quality. Specific conditions, such as eluent mixtures, flow rates and temperatures are provided for each compound individually.

Single crystal X-ray diffraction analysis: Data collection was done on two dual source equipped *Bruker D8 Venture* four-circle-diffractometer from *Bruker AXS GmbH*; used X-ray sources: microfocus *IμS 2.0* Cu/Mo and microfocus *IμS 3.0* Ag/Mo from *Incoatec GmbH* with mirror optics *HELIOS* and single-hole collimator from *Bruker AXS GmbH;* used detector: *Photon III CE14* (Cu/Mo) and *Photon III HE* (Ag/Mo) from *Bruker AXS GmbH*.

Used programs: *APEX4 Suite* (v2022.1-1) for data collection and therein integrated programs *SAINT* V8.40A (Integration) und *SADABS* 2016/2 (Absorption correction) from *Bruker AXS GmbH*; structure solution was done with *SHELXT*, refinement with *SHELXL*-2018/3 (G.M. Sheldrick, *Acta Cryst*. **2008,** *A64*, 112-122. $OLEX^2$ and FinalCif were used for data finalization (O.V. Dolomanov, L.J. Bourhis, R.J Gildea, J.A.K. Howard, H. Puschmann, *J. Appl. Cryst*. **2009,** *42*, 339-341; D. Kratzert, *FinalCif*, *V113*, https://dkratzert.de/finalcif.html.).

Special Utilities: *SMZ1270* stereomicroscope from *Nikon Metrology GmbH* was used for sample preparation; crystals were mounted on *MicroMounts* or *MicroLoops* from *MiTeGen* in NVH oil; crystals were cooled to given temperature with *Cryostream 800* from *Oxford Cryosystems.*

Experimental procedure

Synthesis of the fragment IV

Synthesis of 2

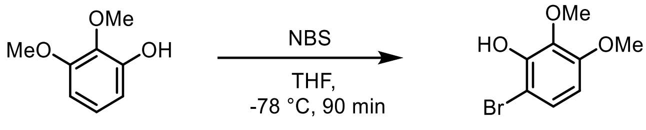

In a 100 mL round-bottom flask 2,3-Dimethoxyphenol (2.50 g, 16.2 mmol, 1.0 eq.) was dissolved in THF (81 mL) and cooled to -78 °C. Then, NBS (2.88 g, 16.2 mmol, 1.0 eq.) was added and the reaction mixture was stirred for 90 min at -78 °C. Afterwards, the reaction was warmed up to room temperature and the solvent was removed under reduced pressure. The crude product was dissolved in ethyl acetate (80 mL) and filtered through celite. Column chromatography (hexane:ethyl acetate 3:1) delivered compound **2** as a brownish oil (3.61 g, 15.6 mmol, 96%).

TLC (SiO_2): R_f = 0.5 (hexane:ethyl acetate 2:1)

^{1}H NMR (300 MHz, $CDCl_3$): δ = 7.15 (d, J= 9.1 Hz, 1H), 6.41 (d, J= 9.1 Hz, 1H), 6.02 (s, 1H), 3.90 (s, 3H), 3.84 (s, 3H) ppm.

^{13}C NMR (75 MHz, $CDCl_3$): δ = 152.2, 147.0, 136.6, 127.0, 105.3, 100.5, 61.2, 56.2 ppm.

HRMS (ESI-TOF) m/z: $[M + H]^+$ Calcd for $C_8H_{11}O_3{}^{79}Br$ 232.9808; Found: 232.9808. Calcd for $C_8H_{11}O_3{}^{81}Br$ 234.9788; Found: 234.9793.

FTIR (ATR, neat): $\tilde{\nu}$ = 3457 (s), 3003 (m), 2944(s), 2834 (s), 1589 (s), 1491 (s), 1463 (s) cm^{-1}.

Synthesis of 3

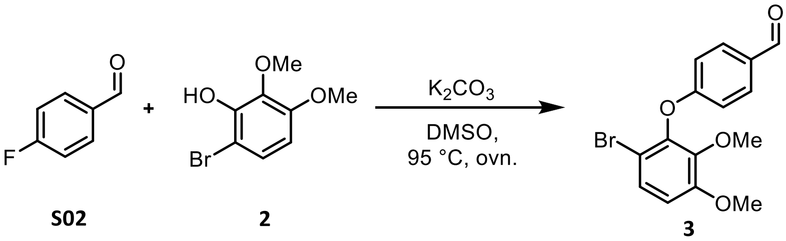

4-Fluorobenzaldehyde (4.40 mL, 41.3 mmol, 1.3 equiv.), 6-bromo-2,3-dimethoxyphenol (**2**) (7.38 g, 31.7 mmol, 1.0 equiv.) and potassium carbonate (8.80 g, 63.7 mmol, 2.0 equiv) were added as solids to a 100 mL Schlenk flask equipped with a magnetic stir bar and subsequently, anhydrous DMSO (75 mL) was added. The flask was then sealed with a septum-cap and the reaction mixture stirred at 95 °C in an oil bath overnight. After cooling to room temperature, water (50 mL) was added and the mixture was extracted with ethyl acetate (100 mL). The combined organic layer was subsequently washed with brine (50 mL) and dried over $MgSO_4$. Removal of the solvent under reduced pressure followed by column chromatography (hexane:ethyl acetate 8:1) delivered compound **3** (4.30 g, 12.8 mmol, 40%) as a colorless oil.

TLC (SiO_2): R_f = 0.38 (hexane:ethyl acetate 3:1)

^{1}H NMR (300 MHz, $CDCl_3$): δ = 9.91 (s, 1H), 7.84 (d, *J*= 8.7 Hz, 2H), 7.34 (d, *J*= 9.0 Hz, 1H), 6.98 (d, *J*= 8.8 Hz, 2H), 6.77 (d, *J*= 9.1 Hz, 1H), 3.89 (s, 3H), 3.75 (s, 3H) ppm.

^{13}C NMR (101 MHz, $CDCl_3$): δ = 190.9, 162.4, 153.7, 145.1, 143.5, 132.1, 131.4, 127.5, 115.8, 110.9, 108.3, 61.3, 56.4 ppm.

FTIR (ATR, neat): $\tilde{\nu}$ = 2938 (m), 2835 (m), 2736 (m), 2361 (m), 1688 (s), 1599 (s), 1501 (s), 1481 (s), 1449 (s), 1420 (s) cm^{-1}.

HRMS (ESI-TOF) m/z: $[M + H]^+$ Calcd for $C_{15}H_{15}O_4{}^{79}Br$ 337.0070; Found: 337.0071. Calcd for $C_{15}H_{15}O_4{}^{81}Br$ 339.0050; Found: 339.0054.

Synthesis of 4

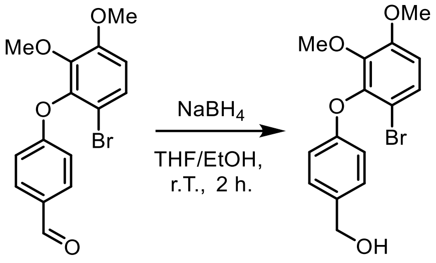

3 **4**

In a Schlenk flask **3** (236 mg, 0.702 mmol, 1.0 equiv.) was dissolved in THF (0.4 mL) and EtOH (2 mL) at 0 °C. Then, sodium borohydride (26.6 mg, 0.702 mmol, 1.0 equiv.) was added. The solution was initially stirred for 30 minutes at 0 °C and subsequently, for two hours at room temperature. Afterwards, water (10 mL) and diethyl ether (10 mL) were added and the aqueous phase was extracted with diethyl ether (3 x 10 mL). The combined organic phase was finally washed with brine (10 mL) and dried over $MgSO_4$. Removal of the solvent in vacuum afforded the crude product as a colorless oil, which was purified by column chromatography (hexane:ethyl acetate 1:1) (193 mg, 0.571 mmol, 84% yield).

TLC (SiO_2): R_f = 0.21 (hexane:ethyl acetate 2:1)

^{1}H NMR (300 MHz, $CDCl_3$): δ = 7.31 (d, *J* = 8.9 Hz, 1H), 7.28 (d, *J* = 8.9 Hz, 2H), 4.62 (d, *J* = 5.7 Hz, 2H), 3.87 (s, 3H), 3.74 (s, 3H), 1.63 – 1.56 (m, 1H) ppm.

^{13}C NMR (75 MHz, $CDCl_3$): δ = 157.3, 153.6, 145.9, 143.8, 134.8, 128.7, 127.3, 115.4, 110.3, 108.9, 65.1, 61.2, 56.4 ppm.

FTIR (ATR, neat): $\tilde{\nu}$ = 3380 (w), 3000 (w), 2931 (m), 2840 (w), 1608 (m), 1575 (m), 1504 (s), 1480 (s), 1450 (s), 1437 (s), 1421 (s) cm^{-1}.

HRMS (ESI-TOF) m/z: $[M + Na]^+$ Calcd for $C_{15}H_{15}O_4{}^{79}BrNa$ 361.0046; Found: 361.0046. Calcd for $C_{15}H_{15}O_4{}^{81}BrNa$ 363.0026; Found: 363.0019.

Synthesis of 5

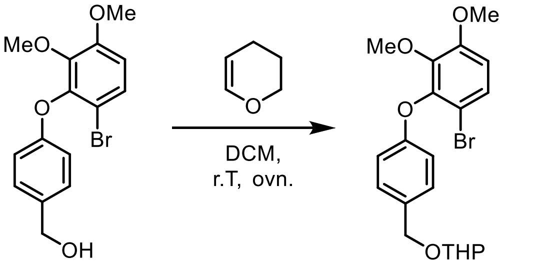

4 **5**

In a Schlenk flask containing **4** (1.00 g, 2.96 mmol, 1.0 equiv.), 3,4-dihydro-2*H*-pyran (0.40 mL, 4.44 mmol, 1.5 equiv.) and pyridinium *p*-toluenesulfonate (74.0 mg, 0.296 mmol, 0.1 equiv.), DCM (9.5 mL) was added and the reaction mixture stirred overnight at room temperature. Subsequently, DCM (20 mL) and water (20 mL) were added to the flask, the phases separated, and the aqueous phase extracted with DCM (3 x 20 mL). The combined organic phase was washed with brine (20 mL) and dried over $MgSO_4$. Afterwards, the solvent was removed under reduced pressure. Compound **5** (1.15 g, 2.72 mmol, 92% yield) was isolated as a yellow oil after purifying by column chromatography (hexane:ethyl acetate 1:2).

TLC (SiO_2): R_f = 0.83 (hexane: ethyl acetate 1:3)

^{1}H NMR (300 MHz, $CDCl_3$): δ = 7.30 (d, J = 8.6 Hz, 1H), 7.27 (d, J = 7.7 Hz, 2H), 6.83 (d, J = 8.6 Hz, 2H), 6.72 (d, J = 8.9 Hz, 1H), 4.72 (d, J = 11.8 Hz, 1H), 4.70 – 4.67 (m, 1H), 4.43 (d, J = 11.8 Hz, 1H), 3.95 – 3.85 (m, 1H), 3.87 (s, 3H), 3.74 (s, 3H), 3.58 – 3.49 (m, 1H), 1.92 – 1.47 (m, 6H) ppm.

^{13}C NMR (101 MHz, $CDCl_3$): δ = 157.2, 153.6, 145.9, 143.8, 132.1, 129.4, 127.2, 115.2, 110.2, 109.0, 97.7, 68.5, 62.2, 61.2, 56.4, 30.7, 25.6, 19.5 ppm.

FTIR (ATR, neat): $\tilde{\nu}$ =2939 (s), 2867 (m), 2851 (m), 2359 (m), 1610 (s), 1575 (s), 1506 (s), 1480 (s), 1450 (s), 1436 (s), 1418 (s) cm^{-1}.

HRMS (ESI-TOF) m/z: $[M + NH_4]^+$ Calcd for $C_{20}H_{27}O_5{}^{79}BrN$ 440.1067; Found: 440.1073. Calcd for $C_{20}H_{27}O_5{}^{81}BrN$ 442.1048; Found: 440.1055.

Synthesis of Fragment III

Synthesis of 2-(2-bromo-5-methoxyphenyl)-1,3-dioxane

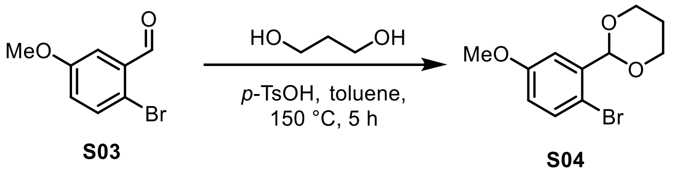

S03 **S04**

In a 100 mL round bottom flask equipped with a Dean-Stark apparatus and a stir bar, 2-Bromo-5-methoxybenzaldehyde (1.33 g, 6.17 mmol, 1.0 equiv.), 1,3-propanediol (900 µL, 12.4 mmol, 2.0 equiv.) and *p*-toluenesulfonic acid monohydrate (0.12 g, 0.62 mmol, 0.1 equiv.) were dissolved in toluene (45 mL) and the reaction mixture refluxed in an oil bath for 5 h. Upon completion, the reaction mixture was cooled to r.t. and sat. aq. $NaHCO_3$ (30 mL) was added. The mixture was extracted using ethyl acetate (100 mL) and the organic phase was washed with brine and dried over $MgSO_4$. After removing the solvent under reduced pressure, column

chromatography (hexane:ethyl acetate 4:1) of the residue yielded compound **S04** (1.60 g, 5.88 mmol, 95%) as a colorless oil.

TLC (SiO_2): R_f = 0.53 (hexane:ethyl acetate 3:1);

^{1}H NMR (300 MHz, $CDCl_3$): δ = 7.40 (d, *J*= 8.8 Hz, 1H), 7.24 (d, *J*= 3.2 Hz, 1H), 6.77 (dd, *J*= 8.8, 3.2 Hz, 1H), 5.72 (s, 1H), 4.31-4.23 (m, 2H), 4.09-3.98 (m, 2H), 3.81 (s, 3H), 2.34-2.16 (m, 1H), 1.50-1.41 (m, 1H) ppm.

^{13}C NMR (75 MHz, $CDCl_3$): δ = 159.2, 138.4, 133.4, 117.3, 112.8, 112.7, 101.0, 67.7, 55.7, 25.8 ppm.

HRMS (ESI-TOF) m/z: [M + H]$^+$ Calcd for $C_{11}H_{15}O_3{}^{79}Br$ 273.0121; Found: 273.0126, *m/z:*[M + H]$^+$ Calcd for $C_{11}H_{15}O_3{}^{81}Br$ 275.0101; Found: 275.0106

FTIR (ATR, neat): $\tilde{\nu}$ = 2963 (s), 2851 (s), 2724 (s), 1594 (s), 1573 (s), 1466 (s) cm^{-1}.

Synthesis of 7

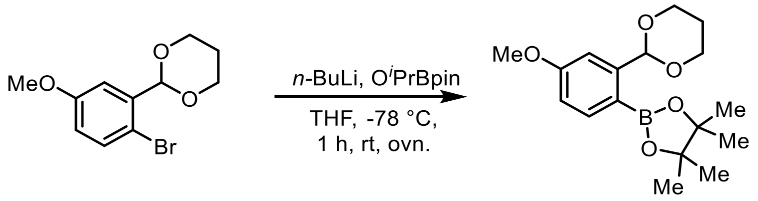

S04 **7**

A 25 mL Schlenk flask with magnetic stir bar was charged with 2-(2-Bromo-5-methoxyphenyl)-1,3-dioxane (800 mg, 2.93 mmol, 1.0 equiv.) and anhydrous THF (15 mL), and cooled to -78 °C. Then, *n*-BuLi (1.28 mL, 3.20 mmol, 1.1 equiv., 2.5 M) was added dropwise and the reaction mixture was stirred for 1 h at -78 °C. Afterwards, isopropoxyboronic acid pinacol ester (650 µL, 3.21 mmol, 1.1 equiv.) was added, and the reaction stirred overnight at r.t. Subsequently, water (20 mL) and ethyl acetate (20 mL) were added, the phases separated, and the aqueous phase extracted with additional ethyl acetate (80 mL). The combined organic phases were then washed with brine and dried over $MgSO_4$. Column chromatography of the residue (hexane:ethyl acetate 2:1) delivered compound **7** (0.690 mg, 2.15 mmol, 74%) as a colorless oil.

TLC (SiO_2): R_f = 0.5 (hexane:ethyl acetate 3:1)

^{1}H NMR (300 MHz, $CDCl_3$): δ = 7.69 (d, *J*= 8.2 Hz, 1H), 7.26 (d, *J*= 2.4 Hz, 1H), 6.83 (dd, *J*= 8.3 Hz, 2.7 Hz, 1H), 6.07 (s, 1H), 4.27-4.19 (m, 2H), 4.07-3.97 (m, 2H), 3.83 (s, 3H), 2.32-2.12 (m, 1H), 1.46-1.37 (m, 1H), 1.34 (s, 12H) ppm.

^{13}C NMR (75 MHz, $CDCl_3$): δ = 162.0, 146.3, 137.2, 114.3, 110.1, 100.3, 83.5, 67.5, 55.3, 26.1, 25.1 ppm.

FTIR (ATR, neat): $\tilde{\nu}$ = 2974 (s), 2933 (w), 2848 (s), 1605 (s), 1568 (s), 1463 (m) cm^{-1}.

HRMS (ESI-TOF) m/z: [M + H]$^+$ Calcd for $C_{17}H_{27}O_5B$ 321.1871; Found: 321.1876.

Synthesis of 6

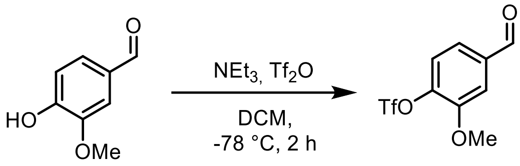

S05 **6**

In a 100 ml Schlenk flask, 4-hydroxy-3-methoxybenzaldehyde (5.00 g, 32.8 mmol, 1.0 equiv.) was dissolved in anhydrous DCM (60 mL) and triethylamine (13.8 mL, 98.6 mmol, 3.0 equiv.) was added. The reaction mixture was cooled to -78 °C and trifluoromethanesulfonic anhydride (6.10 mL, 36.1 mmol, 1.1 equiv.) was added dropwise. The mixture was stirred for further 2 h at -78 °C and afterwards allowed to warm up to room temperature. Water (40 mL) and DCM (40 mL) were then added, the phases separated, and the aqueous phase was extracted with additional DCM (100 mL). The combined organic phases were then washed with brine and dried over $MgSO_4$. Purification of the crude residue by column chromatography (hexane:ethyl acetate 3:1) yielded compound **6** as a colorless oil (7.83 g, 27.6 mmol, 94%).

TLC (SiO_2): R_f = 0.48 (hexane:ethyl acetate 3:1)

^{1}H NMR (300 MHz, $CDCl_3$): δ = 9.98 (s, 1H), 7.56 (d, *J*= 1.8 Hz, 1H), 7.51 (dd, *J*= 8.2 Hz, 1.8 Hz, 1H), 7.41 (d, *J*= 8.2 Hz, 1H), 3.99 (s, 3H) ppm.

^{13}C NMR (101 MHz, $CDCl_3$): δ = 190.5, 152.3, 142.8, 136.9, 124.2, 123.3, 118.8 (q, *J* = 320.3 Hz), 111.9, 56.6 ppm.

FTIR (ATR, neat): $\tilde{\nu}$ = 3078 (w), 2946 (w), 2848 (w), 2738 (w), 1703 (s), 1604 (s), 1498 (s), 1465 (s), 1421 (s) cm^{-1}.

HRMS (EI-orbitrap) m: $[M\ ]^+$ Calcd for $C_9H_7F_3O_5S$ 283.9961; Found: 283.9960.

Synthesis of 8

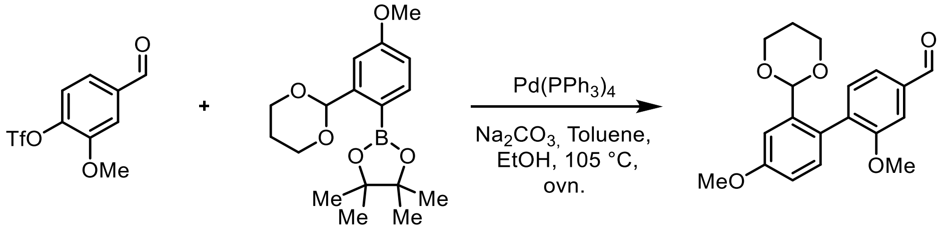

6 **7** **8**

In a 50 mL pressure Schlenk flask, **6** (0.780 g, 2.76 mmol, 1.3 equiv.), **7** (0.680 g, 2.12 mmol, 1.0 equiv.) and Na_2CO_3 (9.74 mL, 0.194 mmol, 9.2 equiv., 2 M in H_2O) were initially suspended in a toluene (19.5 mL) ethanol (9.7 mL) mixture. After thoroughly degassing the flask with nitrogen for 5 min, $Pd(PPh_3)_4$ (318 mg, 0.276 mmol, 0.13 eq.) was added, and the mixture was stirred overnight at 105 °C in an oil bath. After this, the reaction mixture was cooled down to room temperature and filtered through a pad of silica. The solvents were then removed under reduced pressure and the crude product purified using column chromatography (hexane:ethyl acetate 2:1). Compound **8** was obtained as a colorless oil (558 mg, 1.70 mmol, 80%).

TLC (SiO_2): R_f = 0.27 (hexane:ethyl acetate 3:1)

^{1}H NMR (300 MHz, $CDCl_3$): δ = 10.03 (s, 1H), 7.50 (dd, *J*= 7.5 Hz, 1.4 Hz, 1H), 7.49-7.46 (m, 1H), 7.39 (d, *J*= 7.5 Hz, 1H), 7.32 (d, *J*= 2.8 Hz, 1H), 7.12 (d, *J*= 8.5 Hz, 1H), 6.93 (dd, *J*= 8.5 Hz, 1H), 5.17 (s, 1H), 4.24-4.03 (m, 2H), 3.87 (s, 3H), 3.83 (s, 3H), 3.78-3.57 (m, 2H), 2.27-2.09 (m, 1H), 1.35-1.28 (m, 1H) ppm.

^{13}C NMR (101 MHz, $CDCl_3$): δ = 192.1, 159.7, 157.6, 138.1, 137.0, 136.2, 132.6, 131.2, 128.1, 124.0, 115.5, 110.7, 109.0, 100.1, 67.5, 67.4, 55.8, 55.5, 25.7 ppm.

FTIR (ATR, neat): $\tilde{\nu}$ = 2960 (s), 2936 (w), 2843 (s), 2728 (m), 1686 (s), 1609 (s), 1514 (s), 1462 (s) cm^{-1}.

HRMS (ESI-TOF) m/z: $[M + H]^+$ Calcd for $C_{19}H_{22}O_5$ 329.1384; Found: 329.1386.

Synthesis of 9

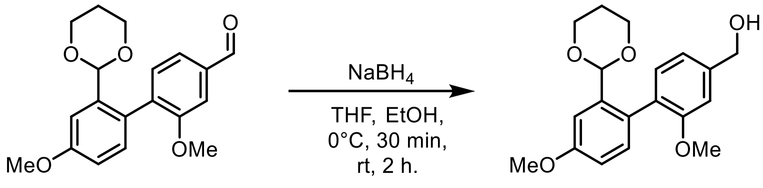

In a 50 mL pressure Schlenk flask, compound **8** (540 mg, 1.65 mmol, 1.0 equiv.) was dissolved in THF (2 mL) and ethanol (10 mL) and cooled to 0 °C. Sodium borohydride (62.0 mg, 1.65 mmol, 1.0 equiv.) was subsequently added and the reaction mixture stirred for 30 min at 0 °C, and subsequently for two additional hours at room temperature. Then, water (10 mL) and diethyl ether (10 mL) were added, the phases separated, and the aqueous phase extracted using diethyl ether (50 mL). The combined organic phases were finally washed with brine and dried over $MgSO_4$. Evaporation of the solvent afforded **9** (530 mg, 1.60 mmol, 97%) as a colorless oil, which was used without further purification.

TLC (SiO_2): R_f = 0.11 (hexane:ethyl acetate 2:1)

^{1}H NMR (400 MHz, $CDCl_3$): δ = 7.30 (d, *J* = 2.8 Hz, 1H), 7.20 (d, *J* = 7.5 Hz, 1H), 7.12 (d, *J* = 8.5 Hz, 1H), 7.02 – 6.95 (m, 2H), 6.91 (dd, *J* = 8.5, 2.8 Hz, 1H), 5.21 (s, 1H), 4.76 (d, *J* = 5.0 Hz, 2H), 4.24 – 4.05 (m, 2H), 3.87 (s, 3H), 3.77 (s, 3H), 3.80 – 3.62 (m, 2H), 2.28- 2.20 (m, 1H), 1.35 – 1.27 (m, 1H) ppm.

^{13}C NMR (101 MHz, $CDCl_3$): δ = 159.2, 157.2, 141.7, 138.1, 132.2, 131.7, 129.1, 128.3, 118.7, 115.5, 110.5, 109.4, 100.3, 67.5, 67.4, 65.5, 55.6, 55.5, 25.8 ppm.

FTIR (ATR, neat): $\tilde{\nu}$ = 3451 (s), 2960 (s), 2936 (s), 2859 (s), 2839 (s), 1734 (s), 1609 (s), 1463 (s) cm^{-1}.

HRMS (ESI-TOF) m/z: $[M + H]^+$ Calcd for $C_{19}H_{24}O_5$ 331.1540; Found: 331.1543.

Synthesis of 10

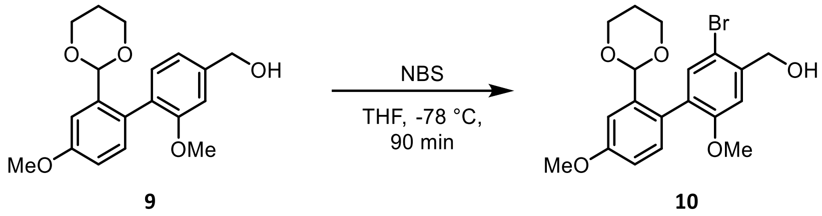

To a solution of **9** (3.92 g, 11.8 mmol, 1.0 equiv.) in THF (59 mL), NBS (2.11 g, 11.8 mmol, 1.0 equiv.) was added at -78 °C and the reaction mixture further stirred at that temperature for 90 min. Afterwards, the reaction was allowed to reach r.t. and then the solvent was removed under reduced pressure. The residue thus obtained was re-dissolved in ethyl acetate (80 mL) and filtered through a small pad of celite. The solvent was removed again under reduced pressure and the residue purified *via* column chromatography (hexane:ethyl acetate 1:1) to deliver compound **10** (3.60 g, 8.80 mmol, 75%) as a colorless oil.

TLC (SiO_2): Rf = 0.13 (hexane:ethyl acetate 2:1)

^{1}H NMR (300 MHz, $CDCl_3$): δ = 7.42 (s, 1H), 7.30 (d, J = 2.8 Hz, 1H), 7.12 (s, 1H), 7.11 (d, J = 8.5 Hz, 1H), 6.91 (dd, J = 8.5, 2.8 Hz, 1H), 5.19 (s, 1H), 4.80 (d, J = 6.2 Hz, 2H), 4.25 - 4.07 (m, 2H), 3.87 (s, 3H), 3.77 (s, 3H), 3.79 – 3.67 (m, 2H), 2.30 – 2.12 (m, 1H), 1.34 (m, 1H) ppm.

^{13}C NMR (75 MHz, $CDCl_3$): δ = 159.5, 156.5, 139.9, 138.1, 135.4, 131.6, 129.7, 127.6, 115.5, 112.0, 110.6, 100.1, 67.5, 67.3, 65.0, 55.9, 55.4, 25.7 ppm.

FTIR (ATR, neat): $\tilde{\nu}$ = 3441 (vs), 2960 (vs), 2933 (vs), 2846 (s), 1734 (s), 1611 (s), 1462 (s) cm^{-1}.

HRMS (ESI-TOF) m/z: $[M + H]^+$ Calcd for $C_{19}H_{23}O_5{}^{79}Br$ 409.0645; Found: 409.0645. m/z: $[M + H]^+$ Calcd for $C_{19}H_{23}O_5{}^{81}Br$ 411.0626; Found: 411.0627.

Synthesis of 11

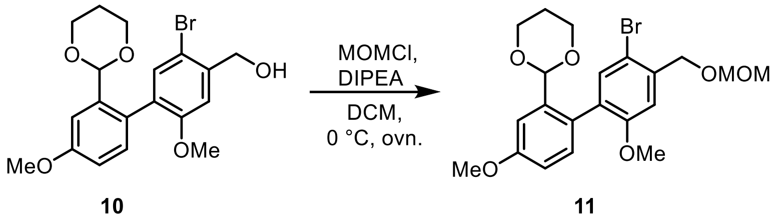

In a Schlenk flask equipped with a magnetic stir bar, compound (**10**) (2.86 g, 7.11 mmol, 1.0 equiv.) was dissolved in anhydrous DCM (30 mL) at 0°C. Then, DIPEA (4.17 mL, 24.9 mmol, 3.5 equiv.) and MOMCl (1.17 mL, 15.6 mmol, 2.2 eq.) were added into the solution and the reaction mixture was stirred, initially for one hour at 0 °C, and afterwards at room temperature overnight. After this, water (20 mL) and DCM (20 mL) were added, the phases separated, and the aqueous phase extracted using DCM (3 x 20 mL).The combined organic phase was finally washed with brine (20 mL) and dried over $MgSO_4$. Purification of the crude product using column chromatography (hexane:ethyl acetate 1:1) afforded compound **11** as a yellow oil (3.20 g, 7.08 mmol, 99% yield).

TLC (SiO_2): R_f = 0.68 (hexane:ethyl acetate 1:3)

^{1}H NMR (300 MHz, $CDCl_3$): δ = 7.43 (s, 1H), 7.30 (d, J = 2.8 Hz, 1H), 7.13 – 7.08 (m, 2H), 6.91 (dd, J = 8.6, 2.59 Hz, 1H), 5.21 (s, 1H), 4.84 (s, 2H), 4.71 (s, 2H), 4.25 – 4.08 (m, 2H), 3.86 (s, 3H), 3.81 – 3.68 (m, 2H), 3.77 (s, 3H), 3.49 (s, 3H), 2.03 – 2.11 (m, 1H), 1.40 – 1.27 (m, 1H) ppm.

^{13}C NMR (101 MHz, $CDCl_3$): δ = 159.5, 156.4, 138.2, 137.4, 135.5, 131.7, 129.9, 127.6, 115.5, 112.6, 111.6, 110.6, 100.1, 96.5, 69.2, 67.5, 67.3, 55.9, 55.8, 55.5, 25.8 ppm.

FTIR (ATR, neat): $\tilde{\nu}$ = 2992 (w), 2949 (m), 2936 (m), 2845 (s), 2249 (m), 1610 (s), 1509 (s), 1461 (s) cm^{-1}.

HRMS (ESI-TOF) m/z: $[M + Na]^+$ Calcd for $C_{21}H_{25}O_6{}^{79}BrNa$ 475.0727; Found: 475.0730. m/z: $[M + Na]^+$ Calcd for $C_{21}H_{25}O_6{}^{81}BrNa$ 477.0707; Found: 475.0712.

Melting point: 79.5-80.5 °C

Preparation of fragment II and subsequent synthesis

Synthesis of 12

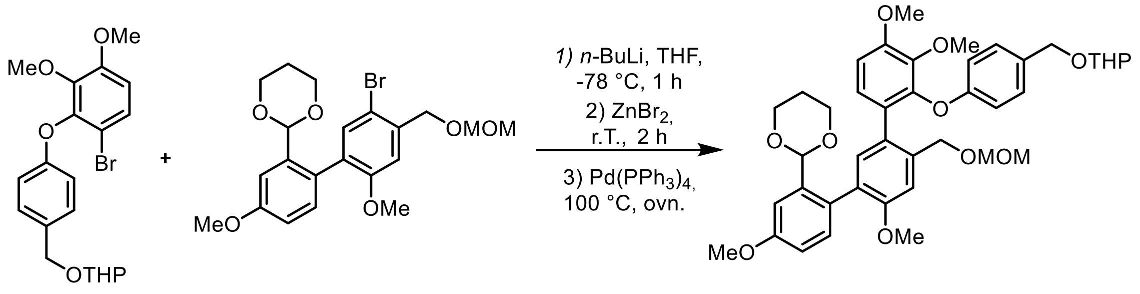

5 **11** **12**

Compound **5** (1.49 g, 3.54 mmol, 1.0 equiv.) was dissolved in THF (38 mL) in a pressure Schlenk and the solution cooled down to -78 °C using an acetone/dry ice bath. Then, *n*-BuLi (1.6 mL, 3.89 mmol, 1.0 eq., 2.5 M) was slowly added and the solution was stirred for one hour at -78 °C. Independently, $ZnBr_2$ (875 mg, 3.89 mmol, 1.0 eq.) was dissolved in THF (2 mL) and slowly added into the first solution, which was then allowed to stir for two hours at room temperature. Afterwards, **11** (1.60 g, 3.54 mmol, 1.0 equiv.) dissolved in THF (2 mL) was added into the reaction mixture followed by $Pd(PPh_3)_4$ (204 mg, 0.170 mmol, 5 mol%). The reaction mixture was then heated up to 100 °C in an oil bath and stirred for 3 days. Then, water (100 mL) and ethyl acetate (100 mL) were added, the phases separated, and the aqueous one extracted with ethyl acetate (3 x 100 mL). Finally, the combined organic phase was washed with brine, dried over $MgSO_4$ and the solvent evaporated. The oil obtained was further purified via column chromatography (hexane:ethyl acetate 2:1 → 1:2). Compound **12** (1.54 g, 2.15 mmol, 61% yield) was isolated as a yellow oil.

TLC (SiO_2): R_f = 0.59 (hexane:ethyl acetate 1:3)

^{1}H NMR (300 MHz, $CDCl_3$): δ = 7.24 (d, J = 2.4 Hz, 1H), 7.10 (d, J = 8.5 Hz, 2H), 7.06 – 6.3 (m, 6H), 7.02 (d, J = 8.9 Hz, 1H), 6.83 (d, J = 8.5 Hz, 1H), 5.32 – 5.02 (m, 1H), 4.75 - 4.47 (m, 6H), 4.39 – 4.27 (m, 1H), 3.96 – 3.88 (m, 1H), 3.91 (s, 3H), 3.84 (s, 3H), 3.74 (s, 6H),

3.57 – 3.45 (m, 1H), 3.36 (s, 3H), 2.28 – 1.96 (m, 1H), 1.90 – 1.44 (m, 7H), 1.39 – 1.07 (m, 3H) ppm.

^{13}C NMR (101 MHz, $CDCl_3$): δ 159.1,157.8, 156.1, 153.2, 146.1, 142.3, 138.1, 136.8, 134.4, 133.6, 131.7, 131.3, 129.0, 128.8, 128.2, 127.7, 125.8, 115.4, 110.3, 110.0, 108.8, 100.3, 97.6, 96.4, 68.7, 68.5, 67.8, 67.3, 62.1, 61.0, 56.2, 55.6, 55.5, 55.4, 30.6, 25.5, 19.4.

FTIR (ATR, neat): $\tilde{\nu}$ = 2938 (s), 2853 (m), 2246 (w), 1605 (s), 1506 (m), 1484 (s), 1460 (m), 1453 (m) cm^{-1}.

HRMS (ESI-TOF) m/z: $[M + NH_4]^+$ Calcd for $C_{41}H_{52}O_{11}N$ 734.3535; Found: 734.3543.

Synthesis of 13

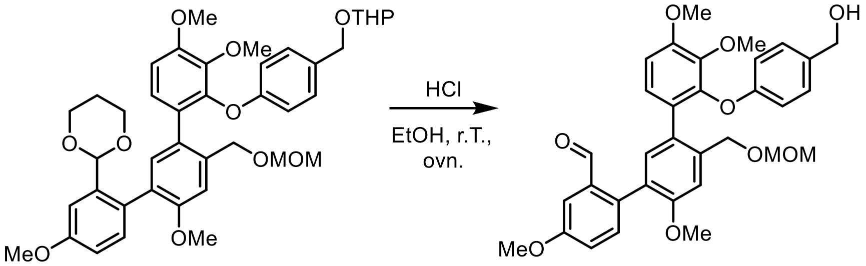

12 13

Compound **12** (2.29 g, 3.19 mmol, 1.0 equiv.) was dissolved in ethanol (46 mL) and hydrochloric acid (9.3 mL, 1 M) was added. The reaction mixture was then stirred at room temperature overnight. After this water (50 mL) and ethyl acetate (50 mL) were added, the phases separated, and the aqueous phase additionally extracted with ethyl acetate (3 x 50 mL). Afterwards, the combined organic phase was further washed using brine (50 mL), dried over $MgSO_4$ and the solvent evaporated in vacuum. Purification of the residue via column chromatography (hexane: ethyl acetate 1:1) afforded **13** as colorless oil (1.53 g, 2.66 mmol, 84% yield).

TLC (SiO_2): R_f = 0.44 (hexane:ethyl acetate 1:3)

^{1}H NMR (300 MHz, $CDCl_3$): δ = 9.22 (d, J = 314.7 Hz, 1H), 7.41 (s, 1H), 7.28 – 7.02 (m, 5H), 6.99 (d, J = 8.5 Hz, 1H), 6.85 (d, J = 8.72 Hz, 1H), 6.82 – 6.40 (m, 3H), 4.79 – 4.42 8m, 6H), 3.93 (s, 3H), 3.85 (s, 6H), 3.72 (s, 3H), 3.36 (s, 3H) ppm.

^{13}C NMR (101 MHz, $CDCl_3$): δ = 193.5, 159.2, 157.5, 156.4, 153.5, 146.0, 142.7, 138.6, 135.5, 135.0, 134.6, 134.0, 132.6, 128.2, 127.2, 125.5, 124.7, 121.9, 115.3, 109.8, 109.2, 109.1, 96.4, 77.4 , 67.5, 64.8, 61.4, 56.3, 55.7, 55.6, 55.5 ppm.

FTIR (ATR, neat): $\tilde{\nu}$ = 2939 (s), 2869 (m), 2845 (m), 2361 (w), 1608 (s), 1575 (s), 1504 (s), 1480 (s), 1450 (s), 1421 (s) cm^{-1}.

HRMS (ESI-TOF) m/z: $[M + Na]^+$ Calcd for $C_{33}H_{34}O_9Na$ 597.2095; Found: 597.2099.

Synthesis of 14

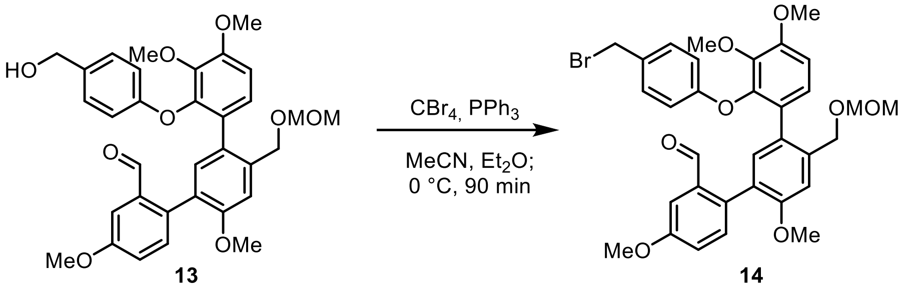

In a Schlenk flask equipped with a magnetic stir bar, compound **13** (338 mg, 0.588 mmol, 1.0 equiv.) was dissolved in a mixture of anhydrous acetonitrile (3 mL) and diethyl ether (3 mL), and cooled down to 0 °C using an ice bath. Then, CBr_4 (215 mg, 0.647 mmol, 1.1 eq.) and PPh_3 (170 mg, 0.647 mmol, 1.1 eq.) were added and the reaction mixture stirred at 0 °C for 90 minutes. Subsequently, water (5 mL) and diethyl ether (5 mL) were added, the phases separated, and the aqueous phase extracted using diethyl ether (3 x 5 mL). The combined organic phase was then dried over $MgSO_4$ and the solvent removed under reduced pressure. The residue thus obtained was further purified by column chromatography (hexane:ethyl acetate 5:1 → 1:2) yielding compound **14** as a yellow oil (300 mg, 0.471 mmol, 80% yield).

TLC (SiO_2): R_f = 0.51 (hexane: ethyl acetate 1:1)

^{1}H NMR (300 MHz, $CDCl_3$): δ = 9.52 (d, J = 133.7 Hz, 1H), 7.44 (d, J = 2.54 Hz, 1H), 7.25 – 7.10 (m, 3H), 7.05 (s, 1H), 7.03 (d, J = 8.5 Hz, 1H), 6.91 (s, 1H), 6.88 (s, 1H), 6.85 (d, J = 8.43 Hz, 1H), 6.75 – 6.51 (m, 2H), 4.67 (s, 2H), 4.50 (s, 2H), 4.41 (s, 2H), 3.94 (s, 3H), 3.86 (s, 3H), 3.81 (s, 3H), 3.73 (s, 3H), 3.37 (s, 3H) ppm.

^{13}C NMR (101 MHz, $CDCl_3$): δ = 192.4, 159.1, 158.3, 156.0, 153.4, 145.8, 142.4, 138.1, 134.7, 133.5, 132.4, 131.1, 130.8, 130.1, 129.7, 128.5, 126.9, 125.7, 125.0, 121.1, 115.7, 115.6, 110.0, 109.3, 109.0, 96.2, 67.3, 61.1, 56.6, 55.5, 46.0, 33.6 ppm.

FTIR (ATR, neat): $\tilde{\nu}$ = 2997 (w), 2936 (m), 2839 (m), 1733 (s), 1688 (s), 1605 (s), 1504 (s), 1484 (s), 1453 (m) cm^{-1}.

HRMS (ESI-TOF) m/z: $[M + Na]^+$ Calcd for $C_{33}H_{33}O_8{}^{79}BrNa$ 659.1251; Found: 659.1224. $[M + Na]^+$ Calcd for $C_{33}H_{33}O_8{}^{81}BrNa$ 661.1233; Found: 661.1210.

Synthesis of 15

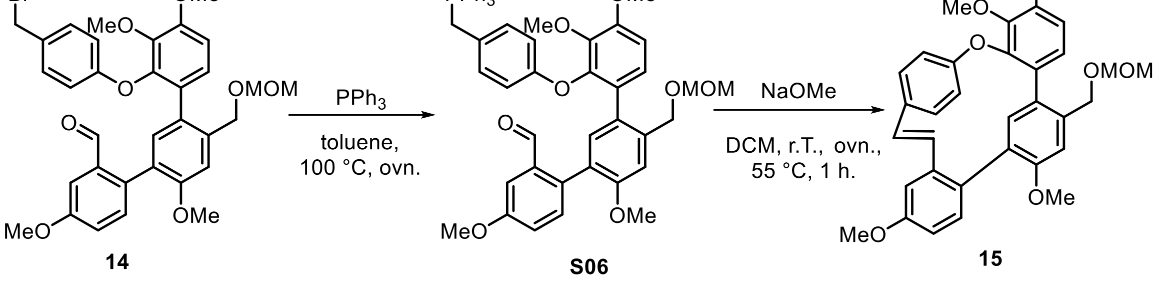

Initially, compound **14** (490 mg, 0.770 mmol, 1.0 equiv.) was dissolved in toluene (11 mL) and then triphenylphosphine (222 mg, 0.840 mmol, 1.1 equiv.) was added. The reaction mixture was stirred at 100 °C in an oil bath overnight causing the precipitation of a white solid. Afterwards, the solvent was removed under reduced pressure, the white solid obtained washed with anhydrous diethyl ether (3 x 20 mL) and finally dried under vacuum. Crude **S06** was used without further purification for the next step.

Subsequently, in a Schlenk flask, sodium methanolate (110 mg, 2.04 mmol, 4 eq.) was dissolved in anhydrous DCM (77 mL). Then, **S06** was dissolved in anhydrous DCM (102 mL) and added over five hours to the first solution using a syringe pump. After this the reaction mixture was stirred overnight at room temperature and subsequently at 55 °C in an oil bath for one additional hour the following day. Finally, the reaction mixture was filtered through a short pad of celite and the solvent was evaporated affording **15** as a yellow oil (160 mg, 0.296 mmol, 38%).

TLC (SiO_2): R_f = 0.34 (hexane:ethyl acetate 1:1)

^{1}H NMR (300 MHz, $CDCl_3$): δ = 7.08 (d, *J* = 8.3 Hz, 1H), 6.99 (s, 1H), 6.93 (d, *J* = 9.1 Hz, 1H), 6.85 (d, *J* = 2.8 Hz, 1H), 6.79 (dd, *J* = 8.3, 2.6 Hz, 1H), 6.71 (s, 2H), 6.65 (d, *J* = 8.6 Hz, 1H), 6.56 (d, *J* = 8.6 Hz, 1H), 6.44 (d, *J* = 8.9 Hz, 1H), 6.10 (d, *J* = 16.8 Hz, 1H), 5.87 (s, 1H), 5.53 (d, *J* = 16.9 Hz, 1H), 4.73 – 4.65 (m, 2H), 4.60 (d, *J* = 6.36 Hz, 1H), 4.35 (d, *J* = 12.7 Hz, 1H), 4.08 (s, 3H), 3.91 (s, 3H), 3.85 (s, 3H), 3.77 (s, 3H), 3.33 (s, 3H) ppm.

^{13}C NMR (101 MHz, $CDCl_3$): δ = 159.8, 159.1, 156.1, 153.0, 151.7, 143.5, 142.5, 139.8, 139.5, 137.6, 134.9, 131.9, 131.2, 131.0, 129.7, 129.5, 127.9, 127.3, 126.3, 124.1, 121.8, 118.8, 111.6, 109.2, 108.8, 107.6, 96.5, 67.5, 61.9, 56.1, 56.0, 55.5, 55.4 ppm.

HRMS (ESI-TOF) m/z: $[M + H]^+$ Calcd for $C_{33}H_{33}O_7$ 541.2221; Found: 541.2210.

HPLC (IG-U, isopropanol/n-hexane = 30/70, flow rate = 0.5 mL/min, l = 254 nm) t_R = 4.3 min (major), 6.9 min (minor) [temp 25°C]

Synthesis of 16

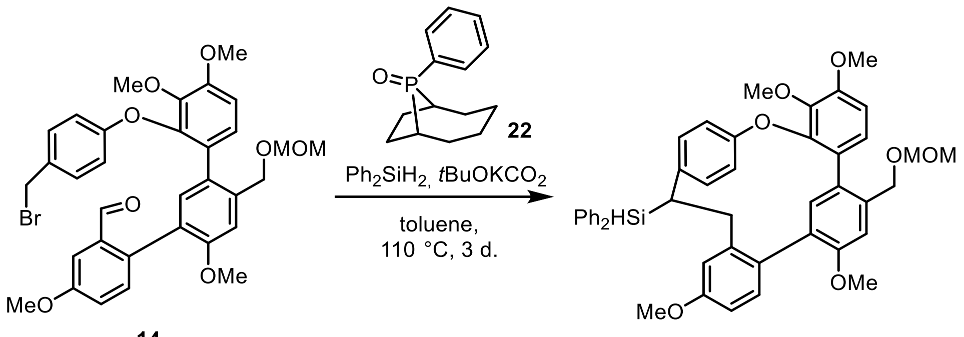

14

16

In a Schlenk flask, compound **14** (280 mg, 0.439 mmol, 1.0 equiv.), potassium *tert*-butylcarbonate (137 mg, 0.878 mmol, 2.0 equiv.) and **22** (29.8 mg, 127 µmol, 0.3 equiv.) were dissolved in anhydrous toluene (110 mL) at room temperature. Then, diphenylsilane (97.8 µL, 0.527 mmol, 1.2 equiv.) was added to the solution and the mixture heated up to 110 °C in an oil bath for three days. After this time, the reaction mixture was filtered through a pad of celite, and the solvent removed under reduced pressure. Purification of the crude thus obtained via

column chromatography yielded compound **16** as a colorless oil (190 mg, 0.262 µmol, 60% yield).

TLC (SiO_2): R_f = 0.37 (hexane:ethyl acetate 1:1)

^{1}H NMR (300 MHz, $CDCl_3$): δ = 7.81 – 7.71 (m, 2H), 7.53 – 7.41 (m, 3H), 7.37 – 7.31 (m, 2H), 7.29 – 7.25 (m, 1H), 7.23 – 7.15 (m, 2H), 6.81 (d, J = 8.98 Hz, 2H), 6.77 (d, J = 8.58 Hz, 1H), 6.72 – 6.65 (m, 2H), 6.63 – 6.54 (m, 1H), 6.47 (d, J = 2.61 Hz, 1H), 6.43 – 6.29 (m, 3H), 5.83 (s, 1H), 5.04 (d, J = 5.76 Hz, 1H), 4.68 (d, J = 6.58 Hz, 1H), 4.62 (d, J = 6.58 Hz, 1H), 4.44 (d, J = 12.31 Hz, 1H), 4.33 (d, J = 12.31 Hz, 1H), 4.01 (s, 3H), 3.93 (s, 3H), 3.75 (s, 3H), 3.75 (s, 3H), 3.34 (s, 3H), 3.13 – 2.88 (m, 2H), 2.48 – 2.40 (m, 1H) ppm.

^{13}C NMR (101 MHz, $CDCl_3$): δ = 159.3, 157.2, 154.3, 153.1, 151.1, 143.4, 141.4, 137.5, 136.4, 136.3, 135.9, 135.5, 133.7, 133.2, 132.0, 130.2, 130.1, 129.8, 129.74, 129.68, 129.5, 128.9, 128.4, 127.9, 126.4, 125.2, 122.5, 118.2, 115.0, 111.8, 108.4, 106.8, 96.5, 77.2, 67.7, 61.7, 56.2, 55.6, 55.4, 55.2, 39.7, 37.6 ppm.

FTIR (ATR, neat): $\tilde{\nu}$ = 2994 (w), 2931 (w), 2834 (m), 1730 (m), 1691 (w), 1599 (s), 1570 (w), 1484 (s) cm^{-1}.

HRMS (ESI-TOF) m/z: $[M + Na]^+$ Calcd for $C_{45}H_{44}O_7SiNa$ 747.2749; Found: 747.2745.

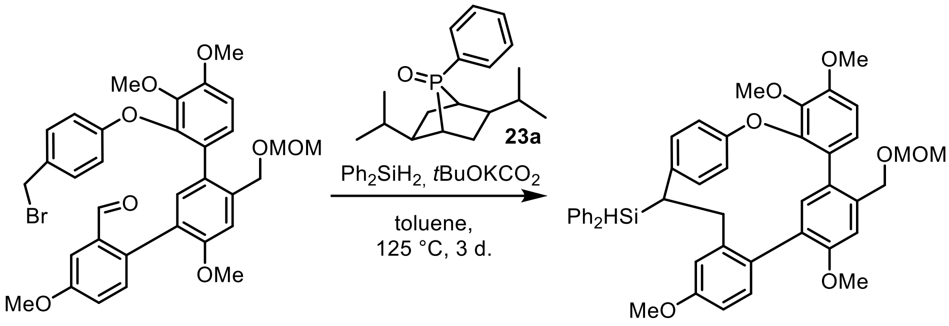

14 16

In a 50 mL pressure Schlenk flask, compound **14** (40 mg, 62.7 µmmol, 1.0 equiv.), potassium *tert*-butylcarbonate (19.6 mg, 126 µmmol, 2.0 equiv.) and phosphine oxide **23a** (5.47 mg, 18.8 µmol, 0.3 equiv.) were dissolved in anhydrous toluene (15.7 mL) at room temperature. Subsequently, diphenylsilane (14.0 µL, 75.3 µmol, 1.2 equiv.) was added into the solution and the mixture heated to 125 °C in an oil bath for three days. Afterwards, the reaction mixture was filtered through a pad of celite and the solvent removed under reduced pressure. Purification of the crude oil thus obtained using column chromatography yielded **16** as a colorless oil (19 mg, 0.262 µmol, 42% yield; 40% ee).

Synthesis of 17

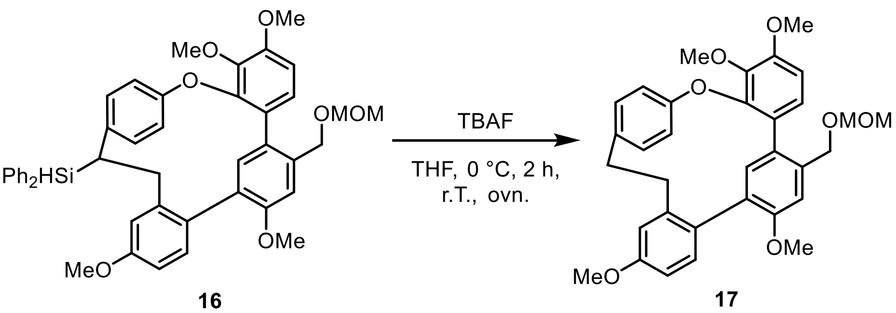

16 17

In a Schlenk flask, compound **16** (0.075 mg, 0.103 mmol, 1.0 equiv.) was dissolved in THF (1 mL) and cooled down to 0 °C. TBAF (0.155 mL, 0.155 mmol, 1.5 eq., 1 M) was then added, the reaction mixture stirred for two hours at 0 °C and subsequently, overnight at room temperature. Afterwards, water (5 mL) and DCM (5 mL) were added, the phases separated, and the aqueous phase extracted with DCM (3 x 5 mL). Finally, the combined organic phases were washed with brine (5 mL) and NH_4Cl (5 mL) and dried over $MgSO_4$. Evaporation of the solvent under reduced pressure afforded an oil that was further purified via column chromatography (hexane:ethyl acetate 5:1 → 1:2). Compound **17** was obtained as a colorless oil (56.1 mg, quant. yield).

TLC (SiO_2): R_f = 0.60 (hexane:ethyl acetate 1:1)

^{1}H NMR (300 MHz, $CDCl_3$): δ = 6.86 (d, J = 8.5 Hz, 1H), 6.83 (d, J = 2.7 Hz, 1H), 6.82 – 6.75 (m, 2H), 6.72 (dd, J = 8.3, 2.7 Hz, 1H), 6.70 (d, J = 8.7 Hz, 1H), 6.59 (dd, J = 8.1, 1.7 Hz, 1H), 6.49 – 6.38 (m, 2H), 6.34 (dd, J = 8.1, 1.8 Hz, 1H), 5.86 (s, 1H), 4.68 (d, J = 6.5 Hz, 1H), 4.63 (d, J = 6.5 Hz, 1H), 4.44 (d, J = 12.3 Hz, 1H), 4.34 (d, J = 12.3 Hz, 1H), 4.00 (s, 3H), 3.93 (s, 3H), 3.83 (s, 3H), 3.78 (s, 3H), 3.35 (s, 3H), 3.17 – 2.97 (m, 2H), 2.88 - 2.75 (m, 1H), 2.41 – 2.27 (m, 1H), 1.63 (s, 1H) ppm.

^{13}C NMR (101 MHz, $CDCl_3$): δ = 159.3, 157.5, 154.2, 153.2, 150.7, 142.0, 141.4, 137.5, 136.6, 136.4, 131.9, 131.1, 129.8, 129.4, 129.1, 128.3, 126.5, 125.0, 122.1, 118.0, 116.0, 111.7, 108.3, 106.8, 96.5, 67.7, 61.7, 56.2, 55.6, 55.4, 55.3, 38.3, 38.1 ppm.

FTIR (ATR, neat): $\tilde{\nu}$ = 2928 (s), 2845 (vs), 1602 (vs), 1506 (s), 1484 (s), 1457 (m), 1418 (m) cm^{-1}.

HRMS (ESI-TOF) m/z: $[M + Na]^+$ Calcd for $C_{33}H_{34}O_7Na$ 565.2197; Found: 565.2191.

Synthesis of 18

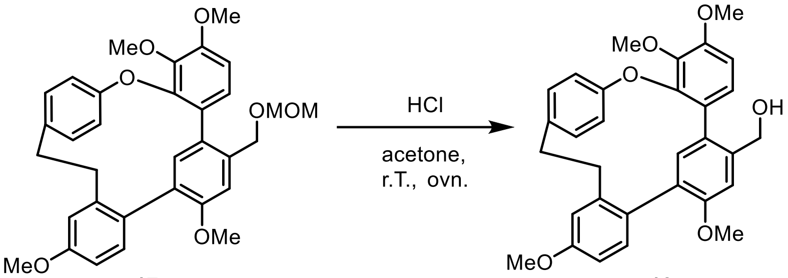

17 **18**

Compound **17** (310 mg, 0.570 mmol, 1.0 equiv.) was dissolved in acetone (15 mL) and subsequently, hydrochloric acid (10 mL, 4 M) was added. This mixture was stirred overnight at room temperature. Afterwards, $NaHCO_3$ (10 mL) was added and the reaction mixture was extracted using DCM (3 x 10 mL). The combined organic phase was then washed with brine (10 mL), dried over $MgSO_4$, and concentrated under reduced pressure. Crude **18** was further purified via column chromatography (hexane:ethyl acetate 5:1 → 1:2) and finally isolated as a colorless oil (210 mg, 0.422 mmol, 74% yield).

TLC (SiO_2): R_f = 0.13 (hexane:ethyl acetate 2:1)

^{1}H NMR (300 MHz, $CDCl_3$): δ = 6.89 - 6.70 (m, 4H), 6.76 – 6.70 (m, 2H), 6.61 (d, J = 8.6 Hz, 1H), 6.47 (dd, J = 8.3, 1.8 Hz, 1H), 6.41 – 6.32 (m, 2H), 5.88 (s, 1H), 4.54 – 4.33 (m, 2H), 4.01

(s, 3H), 3.95 (s, 3H), 3.84 (s, 3H), 3.79 (s, 3H), 3.18 – 2.99 (m, 2H), 2.86 – 2.73 (m, 1H), 2.41 – 2.28 (m, 1H), 1.95 (m, 1H) ppm.

^{13}C NMR (101 MHz, $CDCl_3$): δ = 159.3, 157.3, 154.5, 153.3, 150.6, 142.0, 141.7, 138.7, 137.7, 136.6, 132.0, 130.9, 130.0, 129.4, 129.2, 128.4, 126.3, 125.3, 121.8, 117.7, 116.1, 111.7, 108.8, 107.3, 63.9, 61.7, 56.3, 55.6, 55.3, 38.2 ppm.

FTIR (ATR, neat): $\tilde{\nu}$ = 3476 (w), 2931 (s), 2837 (s), 1602 (s), 1504 (s), 1482 (s), 1460 (s), 1445 (s), 1421 (s), 1383 (s) cm^{-1}.

HRMS (ESI-TOF) m/z: $[M + Na]^+$ Calcd for $C_{31}H_{30}O_6Na$ 521.1935; Found: 521.1936.

Synthesis of 19

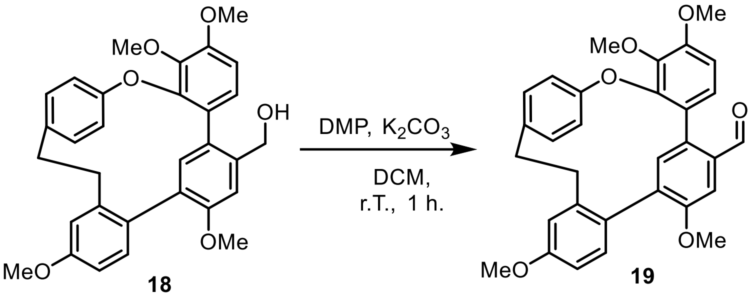

18 **19**

In a Schlenk flask equipped with a magnetic stir bar, compound **18** (65.0 mg, 0.130 mmol, 1.0 equiv.) was dissolved in anhydrous DCM (1.5 mL), and subsequently, DMP (83.0 mg, 0.190 mmol, 1.5 eq.) and K_2CO_3 (0.072 mg, 0.520 mmol, 4.0 eq.) were added. The reaction mixture was stirred for one hour at room temperature. Afterwards, water (2 mL) was added, the phases separated, and the aqueous phase was extracted with DCM (3 x 5 mL). The combined organic phase was dried over $MgSO_4$ and concentrated under reduced pressure. The crude oil thus obtained was further purified via column chromatography (hexane: ethyl acetate 5:1 → 1:2) to deliver **19** as a colorless oil (63 mg, 98% yield).

TLC (SiO_2): R_f = 0.71 (hexane:ethyl acetate 1:1)

^{1}H NMR (300 MHz, $CDCl_3$): δ = 9.77 (s, 1H), 7.22 (s, 1H), 6.95 – 6.87 (m, 3H), 6.83 – 6.79 (m, 2H), 6.6 (dd, J = 8.4, 2.3 Hz, 1H), 6.5 (dd, J = 8.1, 2.6 Hz, 1H), 6.4 (dd, J = 8.1, 2.3 Hz, 1H), 6.3 (dd, J = 8.4, 2.6 Hz, 1H), 6.04 (s, 1H), 4.07 (s, 3H), 4.01 (s, 3H), 3.89 (s, 3H), 3.87 (s, 3H), 3.19 – 3.08 (m, 2H), 2.83 – 2.74 (m, 1H), 2.42 – 2.32 (m, 1H) ppm.

^{13}C NMR (101 MHz, $CDCl_3$): δ = 192.3, 159.9, 157.3, 154.7, 153.8, 151.4, 141.8, 141.4, 138.7, 136.7, 136.4, 135.4, 133.7, 131.6, 130.2, 130.1, 128.6, 126.4, 122.5, 118.5, 116.3, 112.0, 106.9, 106.1, 61.8, 56.3, 55.7, 55.4, 38.3, 38.0.

FTIR (ATR, neat): $\tilde{\nu}$ =2933 (s), 2838 (s), 2361 (s), 2340 (s), 1682 (s), 1600 (s), 1504 (m), 1482 (s), 1460 (m), 1421 (m) cm^{-1}.

HRMS (ESI-TOF) m/z: $[M + H]^+$ Calcd for $C_{32}H_{28}O_6$ 497.1959; Found: 497.1960.

Melting point: 179.0-180.0 °C.

Synthesis of 20

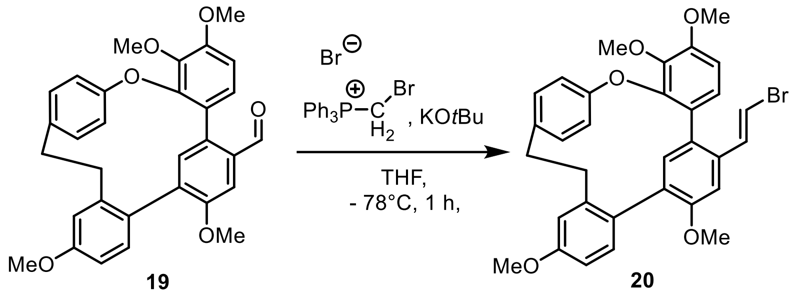

In a Schlenk flask, (bromomethyl)triphenylphosphonium bromide (58.0 mg, 0.133 mmol, 1.1 equiv.) and KO*t*Bu (17.6 mg, 0.157 mmol, 1.3 equiv.) were dissolved in anhydrous THF (0.5 mL) at -78 °C. Then, compound **19** (60.0 mg, 0.121 mmol, 1.0 eq.) dissolved in anhydrous THF (1.0 mL) was added to the first solution and the mixture stirred for one hour at -78 °C. Subsequently, the reaction mixture was allowed to warm to room temperature; then, water (5 mL) and ethyl acetate (5 mL) were added, the phases separated and the aqueous phase extracted with ethyl acetate (3 x 10 mL). The combined organic phase was then washed with brine (10 mL), dried over $MgSO_4$, and finally, the solvent was evaporated under reduced pressure. The oil thus obtained was purified via column chromatography (hexane:ethyl acetate 5:1 → 1:2) to deliver **20** as a colorless oil (50.0 mg, 87.4 mmol, 73% yield).

TLC (SiO_2): R_f = 0.83 (hexane:ethyl acetate 1:3)

^{1}H NMR (400 MHz, $CDCl_3$): δ = 7.45 (s, 1H), 6.89 (d, J = 8.5, 1H), 6.84 (d, J = 2.7, 1H), 6.78 (d, J = 6.8 Hz, 1H), 6.76 (d, J = 6.9 Hz, 1H), 6.74 (dd, J = 8.4, 2.6 Hz, 1H), 6.70 (d, J = 8.3, 1H), 6.62 (dd, J = 8.5, 2.0 Hz, 1H), 6.51 (dd, J = 8.3, 2.6 Hz, 1H), 6.4 (dd, J = 8.1, 2.6 Hz, 1H), 6.36 – 6.30 (m, 2H), 5.91 (s, 1H), 4.01 (s, 3H), 3.94 (s, 3H), 3.84 (s, 3H), 3.81 (s, 3H), 3.15 – 2.99 (m, 2H), 2.88 – 2.80 (m, 1H), 2.39 – 2.29 (m, 1H) ppm.

^{13}C NMR (101 MHz, $CDCl_3$): δ = 159.4, 157.4, 153.4, 153.3, 151.0, 142.0, 141.3, 137.4, 136.6, 133.2, 131.7, 131.2, 131.0, 130.9, 130.2, 130.0, 128.3, 126.2, 125.2, 122.8, 118.1, 116.1, 111.8, 109.0, 106.7, 106.4, 61.7, 56.2, 55.7, 55.3, 38.3, 38.1 ppm.

FTIR (ATR, neat): $\tilde{\nu}$ = 3278 (s), 2925 (s), 2853 (s), 1602 (s), 1504 (s), 1485 (s), 1463 (s) cm^{-1}.

HRMS (ESI-TOF) m/z: $[M + Na]^+$ Calcd for $C_{32}H_{29}O_5{}^{79}BrNa$ 595.1091; Found: 595.1085. Calcd for $C_{32}H_{29}O_5{}^{81}BrNa$ 597.1072; Found: 595.1071.

Synthesis of 21

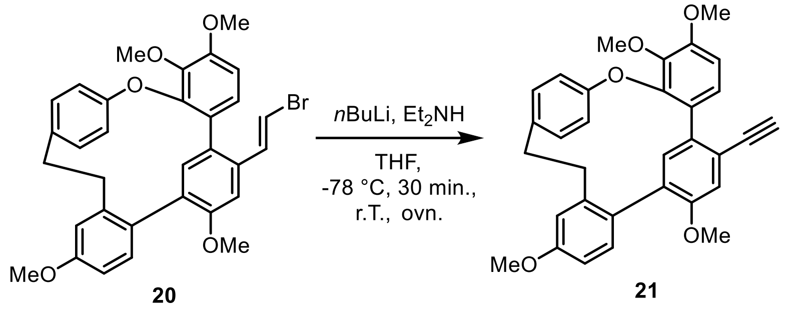

In a Schlenk flask containing anhydrous THF (0.5 mL), freshly distilled diethyl amine (7.54 µL, 73.2 µmol, 2.1 equiv.) was added and cooled down to - 78 °C. Then, *n*-BuLi (30.0 µL, 73.2 µmol, 2.1 equiv., 2.5 M) was added and the solution stirred for 30 min. at -78 °C. Subsequently, vinyl bromide **20** (20.0 mg, 34.9 µmol, 1.0 equiv.) dissolved in THF (0.5 mL) was added to the original solution, and the mixture stirred overnight at r.t. Afterwards, water (2 mL) and ethyl acetate (2 mL) were added, the phases separated, and the aqueous phase extracted with ethyl acetate (3 x 5 mL). The combined organic phase was then washed with brine (5 mL), dried over $MgSO_4$ and the solvent evaporated. Crude **21** was purified by column chromatography (hexane:ethyl acetate 5:1 → 1:2), and finally obtained as a colorless oil (17.0 mg, 34.5 µmol, 99% yield).

TLC (SiO_2): R_f = 0.81 (hexane:ethyl acetate 1:3)

^{1}H NMR (300 MHz, $CDCl_3$): δ = 6.87 (d, J = 8.56 Hz, 1H), 6.84 (d, J = 2.60 Hz, 1H), 6.80 – 6.71 (m, 3H), 6.70 (d, J = 8.38 Hz, 1H), 6.67 – 6.55 (m, 2H), 6.44 (dd, J = 8.15, 2.21 Hz, 1H), 6.34 (dd, J = 8.15, 1.79 Hz, 1H), 5.91 (s, 1H), 4.02 (s, 3H), 3.94 (s, 3H), 3.84 (s, 3H), 3.76 (s, 3H), 3.17 – 2.99 (m, 2H), 2.98 (s, 1H), 2.84 – 2.72 (m, 1H) 2.39 – 2.28 (m, 1H) ppm.

^{13}C NMR (101 MHz, $CDCl_3$) δ = 159.5, 157.6, 153.6, 153.2, 151.3, 141.9, 141.1, 137.2, 136.5, 134.6, 131.7, 131.2, 130.5, 129.9, 128.3, 126.0, 125.2, 122.6, 121.6, 118.3, 116.1, 112.8, 111.8, 106.3, 82.7, 79.3, 61.6, 56.2, 55.6, 55.3, 38.2, 38.1 ppm.

FTIR (ATR, neat): $\tilde{\nu}$ = 3284 (s), 3000 (m), 2933 (s), 2837 (s), 2361 (w), 2249 (m), 1599 (s), 1504 (s), 1482 (s), 1445 (s) cm^{-1}.

HRMS (ESI-TOF) m/z: $[M + H]^+$ Calcd for $C_{32}H_{29}O_5$ 493.2010; Found: 493.2014.

Synthesis of 24

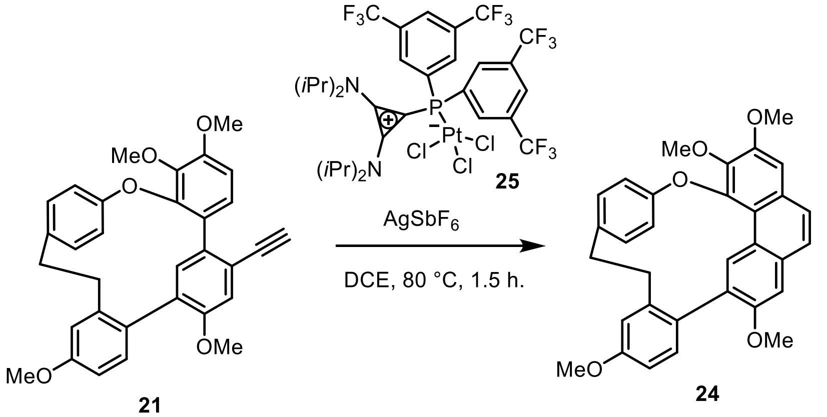

In a Schlenk flask equipped with a magnetic stir bar, alkyne **21** (15.0 mg, 30.5 µmol, 1.0 equiv.) and platinum catalyst **25** (2.10 mg, 2.00 µmol, 5mol%) were dissolved in anhydrous DCE (1 mL). Then, a solution of $AgSbF_6$ in anhydrous DCE (0.040 µL, 2.00 µmol, 5mol%, 0.05 M) was added, and the reaction mixture stirred for 1.5 h at 80 °C in an oil bath. Afterwards, the mixture was filtered through a pad of silica and the solvent removed under reduced pressure. Purification of crude **24** via column chromatography (hexane:ethyl acetate 100% hexane → 40:60) delivered an orange solid (12.0 mg, 24.4 µmol, 81% yield).

TLC (SiO_2): R_f = 0.85 (hexane:ethyl acetate 1:3)

^{1}**H NMR** (300 MHz, $CDCl_3$): δ = 7.63 (d, J = 8.71 Hz, 1H), 7.56 (d, J = 8.74 Hz, 1H), 7.34 (s, 1H), 7.19 (s, 1H), 7.03 (s, 1H), 6.94 (s, 1H), 6.90 (dd, J = 8.34, 2.74 Hz, 1H), 6.79 (s, 1H), 6.33 (dd, J = 8.60, 2.17 Hz, 1H), 6.23 (dd, J = 8.80, 2.15 Hz, 1H), 5.75 (dd, J = 8.50, 2.38 Hz 1H), 4.08 (s, 3H), 4.05 (s, 3H), 3.92 (s, 3H), 3.81 (s, 3H), 3.05 – 2.92 (m, 1H), 2.90 – 2.80 (m, 1H), 2.71 – 2.57 (m, 1H), 2.09 – 1.93 (m, 1H) ppm.

^{13}**C NMR** (101 MHz, $CDCl_3$): δ = 158.9, 155.9, 154.2, 152.0, 145.0, 143.3, 142.6, 134.9, 132.3, 131.9, 130.5, 130.1, 129.6, 129.4, 128.4, 127.3, 126.4, 121.3, 117.8, 117.0, 115.2, 113.1, 111.3, 105.9, 62.2, 56.1, 55.5, 55.3, 38.14, 38.07 ppm.

FTIR (ATR, neat): $\tilde{\nu}$ = 2936 (m), 2914 (m), 2848 (w), 2831 (m), 2356 (m), 1599 (s), 1498 (s), 1469 (s) cm^{-1}.

HRMS (ESI-TOF) m/z: $[M + H]^+$ Calcd for $C_{32}H_{29}O_5$ 493.2010; Found: 493.2009.

Synthesis of 1

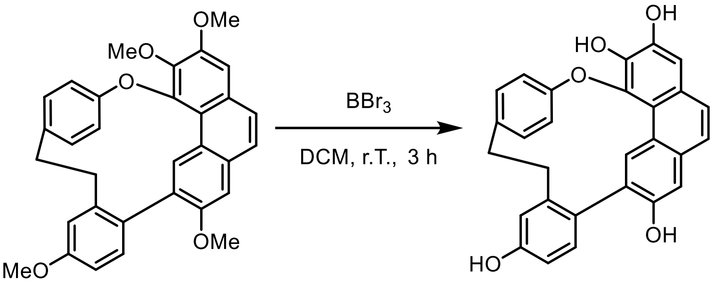

24 **angustatin (1)**

In a Schlenk flask equipped with magnetic stir bar, **24** (10.0 mg, 20.3 µmol, 1.0 eq.) was dissolved in anhydrous DCM (1 mL) and cooled down to 0 °C. A solution of BBr_3 in DCM (0.20 mL, 20 µmol, 10.0 eq., 1 M) was then slowly added to the initial solution, and the reaction mixture stirred at r.t. for three hours. Afterwards, methanol (2 mL) was added, and subsequently, the solvent removed under reduced pressure. Purification of crude **1** via column chromatography (hexane:ethyl acetate 1:2.5) yielded angustatin A as a yellow oil (8.41 mg, 19.3 µmol, 95% yield).

TLC (SiO_2): R_f = 0.58 (hexane:ethyl acetate 1:3)

^{1}**H NMR** (300 MHz, acetone-d6): δ = 8.79 (s, 1H), 8.27 (s, 1H), 8.15 (s, 1H), 7.79 (s, 1H), 7.54 (d, J = 8.72 Hz, 1H), 7.38 (d, J = 8.76 Hz, 1H), 7.24 (s, 1H), 7.16 (s, 1H), 7.06 (s, 1H), 6.89 (d, J = 2.49 Hz, 1H), 6.76 – 6.68 (m, 2H), 6.65 (d, J = 8.22 Hz, 1H), 6.37 (dd, J = 8.51, 2.17 Hz, 1H), 6.22 (dd, J = 8.37, 2.18 Hz, 1H), 5.75 (dd, J = 8.46, 2.65 Hz, 1H), 2.95 – 2.81 (m, 2H), 2.67 – 2.55 (m, 1H), 2.18 – 2.07 (m, 1H) ppm.

^{13}**C NMR** (101 MHz, CD_2Cl_2) δ = 156.3, 155.2, 150.4, 144.0, 142.8, 138.1, 137.3, 136.0, 133.4, 132.2, 130.7, 129.6, 129.3, 129.1, 127.8, 127.2, 127.0, 125.1, 121.1, 118.1, 117.1, 116.0, 114.8, 114.3, 110.3, 109.7, 77.9, 60.8, 38.4, 38.0, 30.1 ppm.

FTIR (ATR, neat): $\tilde{\nu}$ = 3497 (b), 3361 (b), 2920 (s), 2851 (s), 1602 (s), 1567 (s), 1501 (s) cm^{-1}.

HRMS (ESI-TOF) m/z: $[M - H]^+$ Calcd for $C_{28}H_{19}O_5$ 435.1238; Found: 435.1238.

HPLC (IA-3, isopropanol/n-hexane = 50/50, flow rate = 1.0 mL/min, l = 254 nm) t_R = 3.9 min (major), 16.7 min (minor) [temp 25°C]

$^1H/^{13}C$ NMR Data comparison with data available

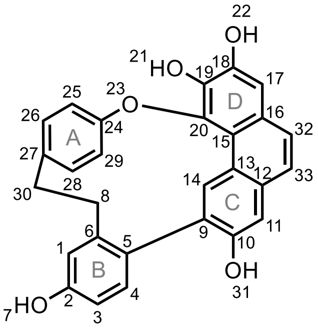

All values in ppm. Compared with 'J.-B. Qu, L.-m. Sun, H.-X. Lou, *Chin. Chem. Lett.* **2013**, *24*, 801.

Atom number	^1H (Lit.)	^1H (600 MHz)	^{13}C (Lit.)	^{13}C (125 MHz)
**1**			117.0	117.0
**2**			157.5	157.5
**3**	6.71	6.71	114.0	114.0
**4**	6.65	6.65	131.3	131.2
**5**			131.5	131.4
**6**			143.3	143.3
**7**	-	8.27		
**8**	2.90	2.90; 2.12	38.7	38.7
**9**			129.0	129.0
**10**	6.89	6.89	152.4	152.4
**11**	7.06	7.06	110.9	110.9
**12**			127.1	127.2
**13**			121.3	121.3
**14**	7.16	7.16	130.5	130.6
**15**			116.6	116.6
**16**			133.4	133.5
**17**	7.25	7.24	110.2	110.2
**18**			146.1	146.1
**19**			140.3	140.3
**20**			138.1	138.1
**21**	-	8.77		
**22**	-	8.01		
**23**				
**24**			156.3	156.3
**25**	6.73	6.72	118.3	118.3
**26**	6.22	6.22	131.1	131.0
**27**			135.8	135.8
**28**	6.38	6.37	129.3	129.3
**29**	5.75	5.75	114.1	114.1
**30**	2.61	2.91; 2.60	38.6	38.6
**31**	-	7.79		
**32**	7.54	7.54	127.8	127.8
**33**	7.38	7.38	124.9	124.9

Spectra

Compound 2

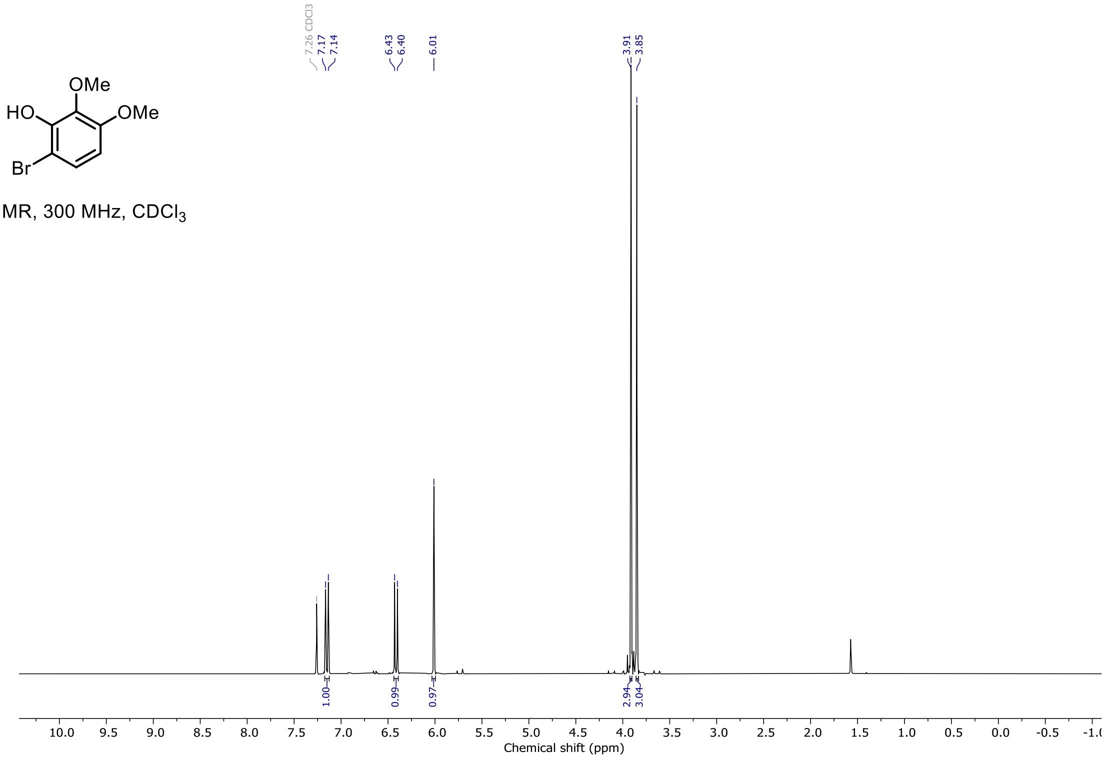

^{1}H NMR, 300 MHz, $CDCl_3$

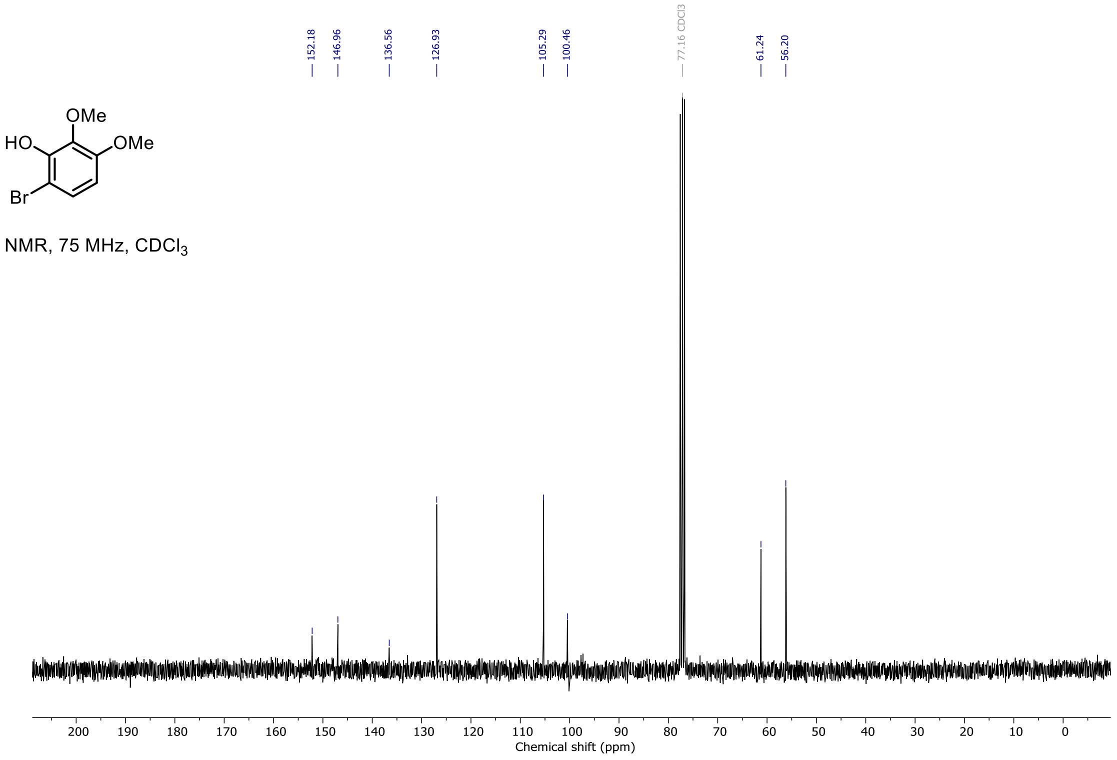

^{13}C NMR, 75 MHz, $CDCl_3$

Compound 3

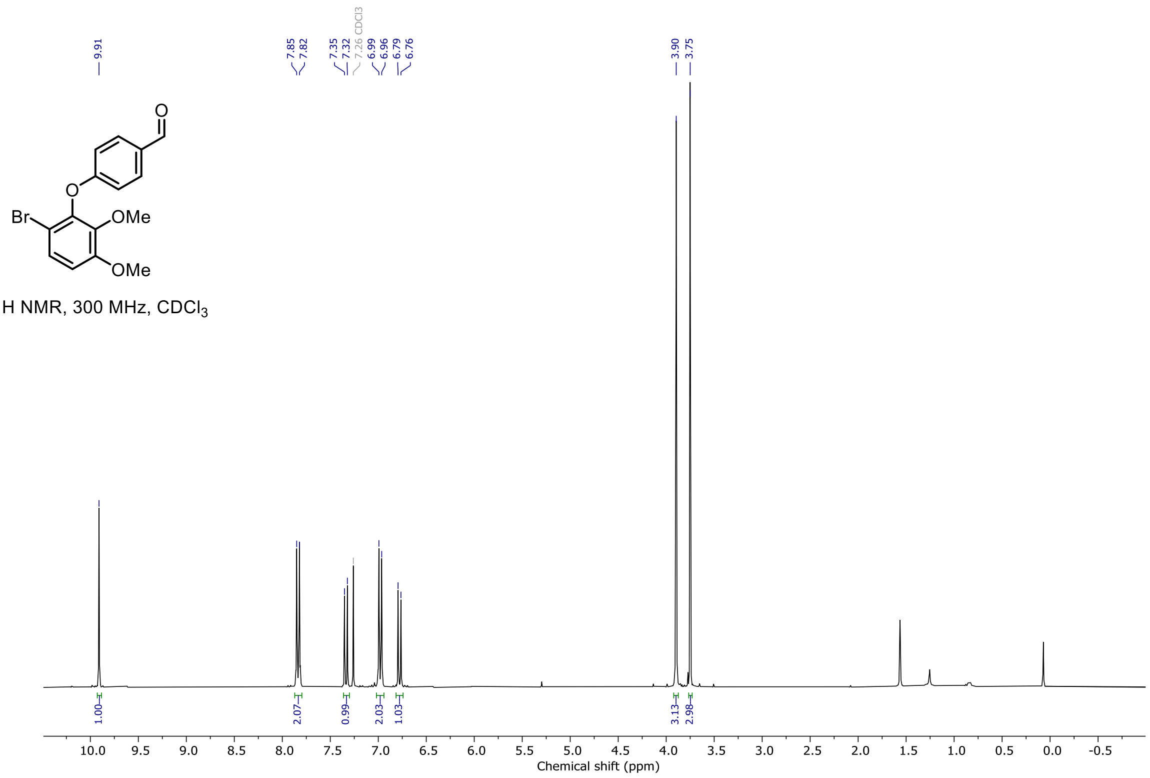

^{1}H NMR, 300 MHz, $CDCl_3$

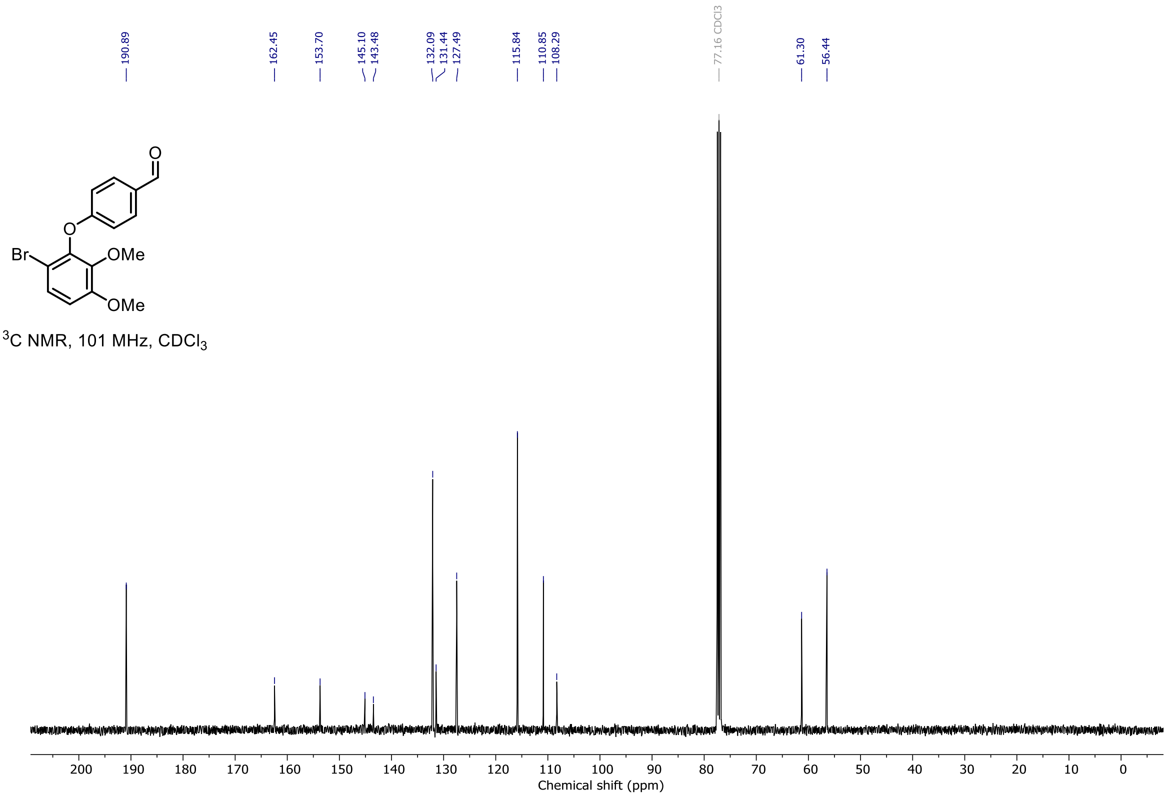

^{13}C NMR, 101 MHz, $CDCl_3$

Compound 4

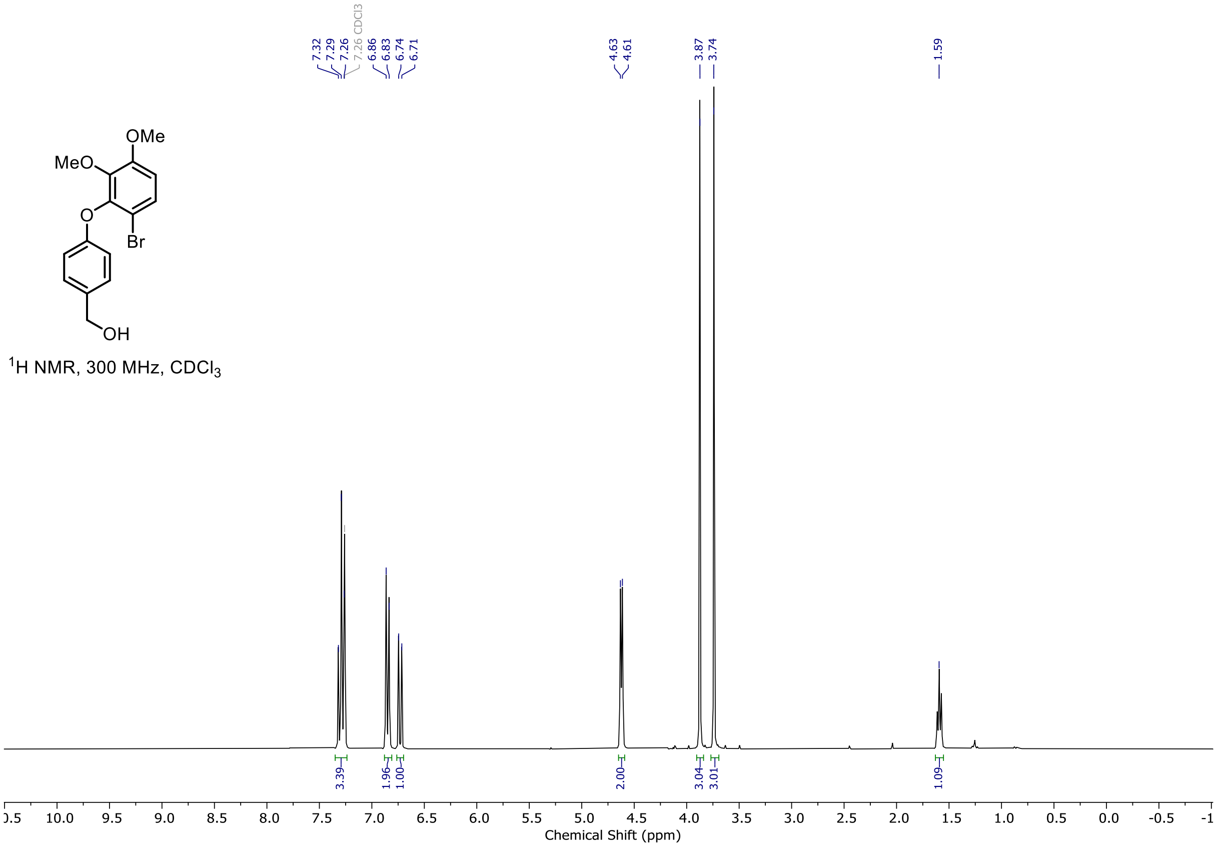
7.32
7.29
7.26
7.26 CDCl3
6.86
6.83
6.74
6.71
4.63
4.61
3.87
3.74
1.59
OMe
MeO
O
Br
OH
^{1}H NMR, 300 MHz, $CDCl_3$
3.39
1.96
1.00
2.00
3.04
3.01
1.09
Chemical Shift (ppm)

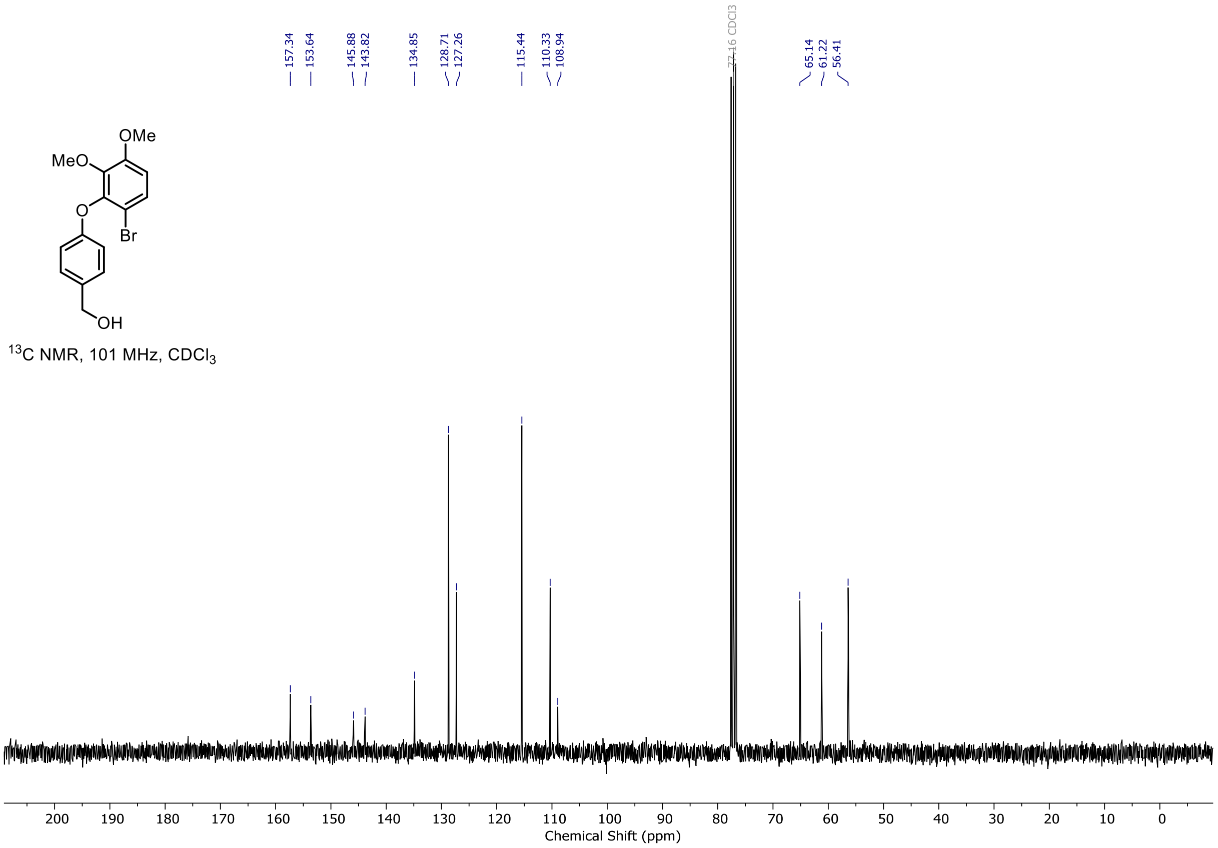
157.34
153.64
145.88
143.82
134.85
128.71
127.26
115.44
110.33
108.94
77.16 CDCl3
65.14
61.22
56.41
OMe
MeO
O
Br
OH
^{13}C NMR, 101 MHz, $CDCl_3$
Chemical Shift (ppm)

Compound 5

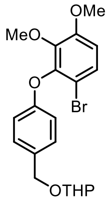

^{1}H NMR, 300 MHz, $CDCl_3$

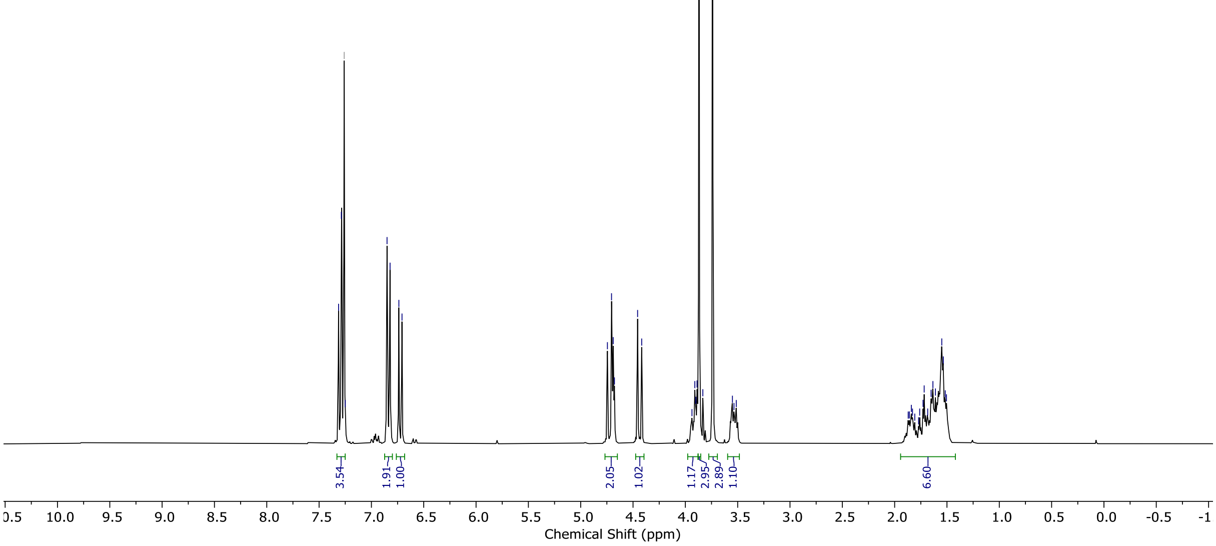

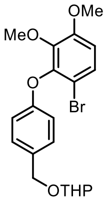

^{13}C NMR, 101 MHz, $CDCl_3$

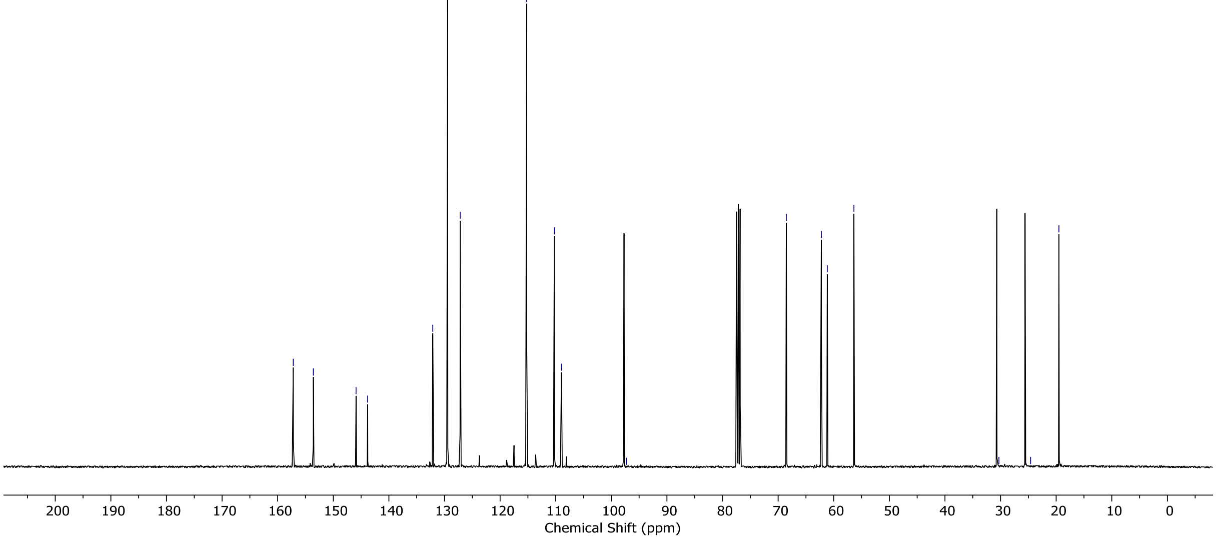

Compound S04

^{1}H NMR, 300 MHz, $CDCl_3$

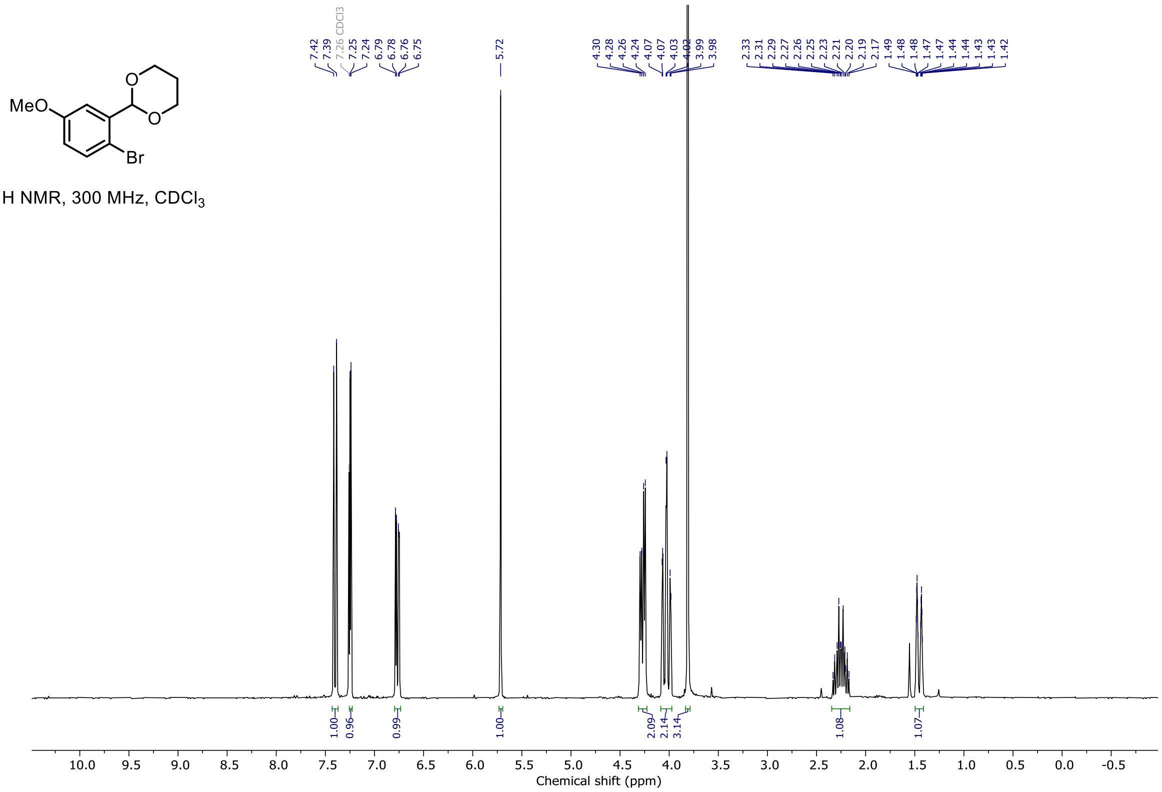

^{13}C NMR, 75 MHz, $CDCl_3$

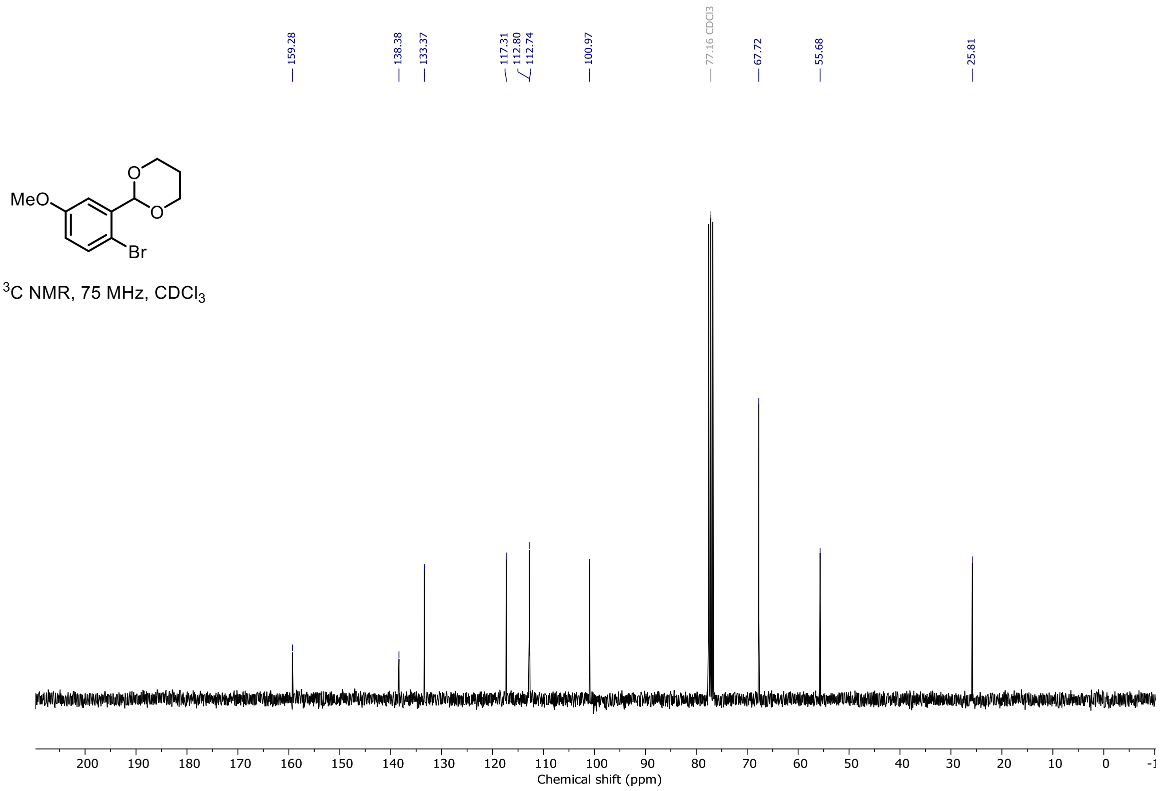

Compound 6

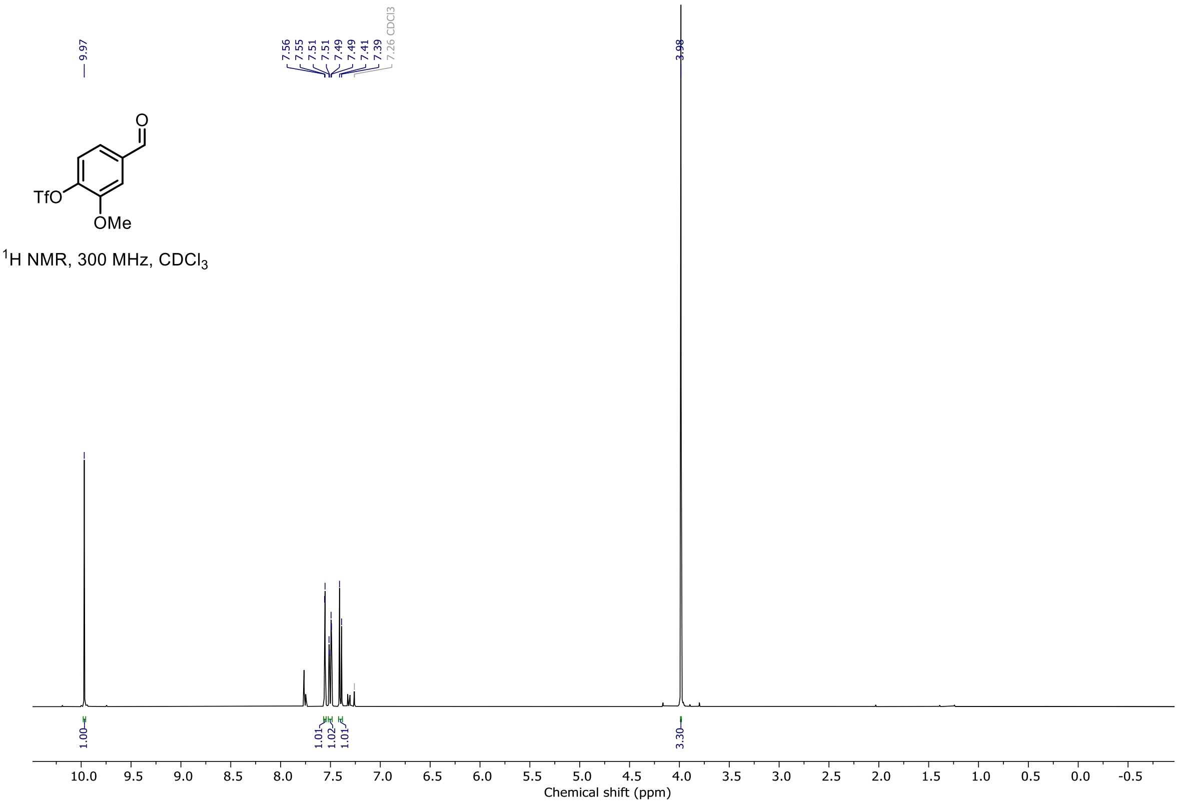
9.97
7.56
7.55
7.51
7.51
7.49
7.49
7.41
7.39
7.26 CDCl3
3.98
TfO
OMe
O
^{1}H NMR, 300 MHz, $CDCl_3$
1.00
1.01
1.02
1.01
3.30
10.0 9.5 9.0 8.5 8.0 7.5 7.0 6.5 6.0 5.5 5.0 4.5 4.0 3.5 3.0 2.5 2.0 1.5 1.0 0.5 0.0 -0.5
Chemical shift (ppm)

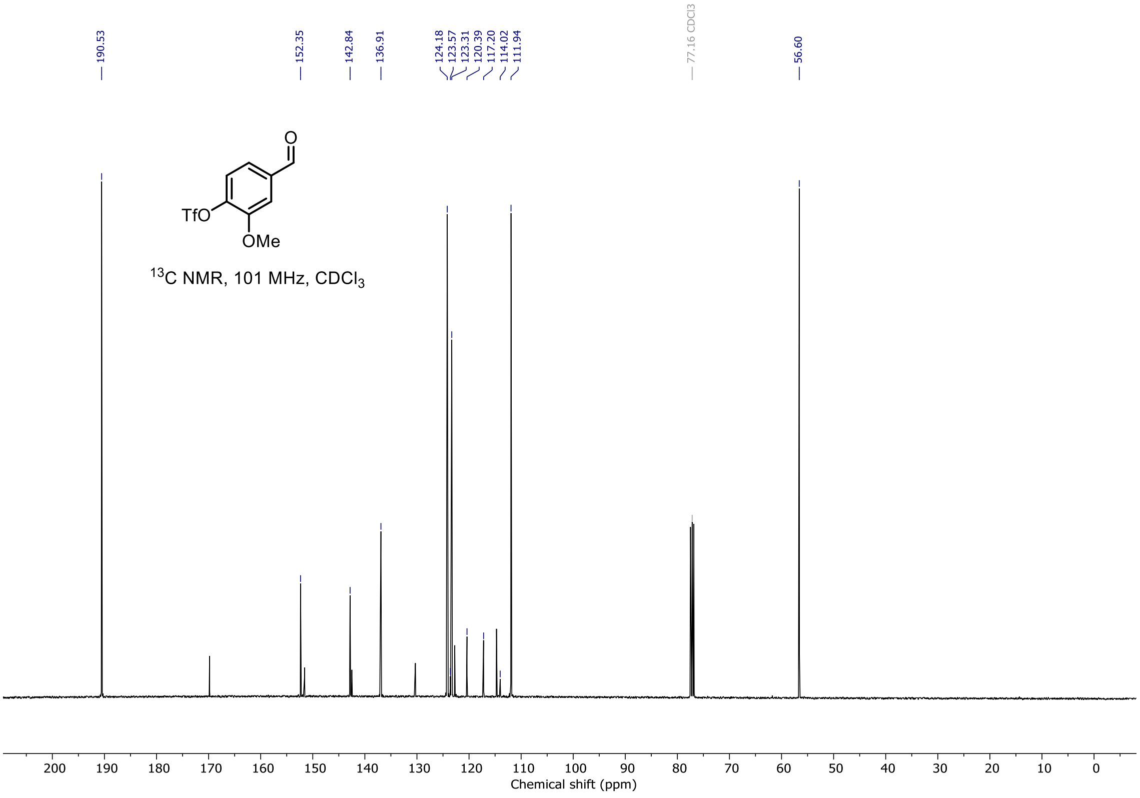
190.53
152.35
142.84
136.91
124.18
123.57
123.31
120.39
117.20
114.02
111.94
77.16 CDCl3
56.60
TfO
OMe
O
^{13}C NMR, 101 MHz, $CDCl_3$
200 190 180 170 160 150 140 130 120 110 100 90 80 70 60 50 40 30 20 10 0
Chemical shift (ppm)

Compound 7

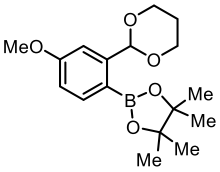

^{1}H NMR, 300 MHz, $CDCl_3$

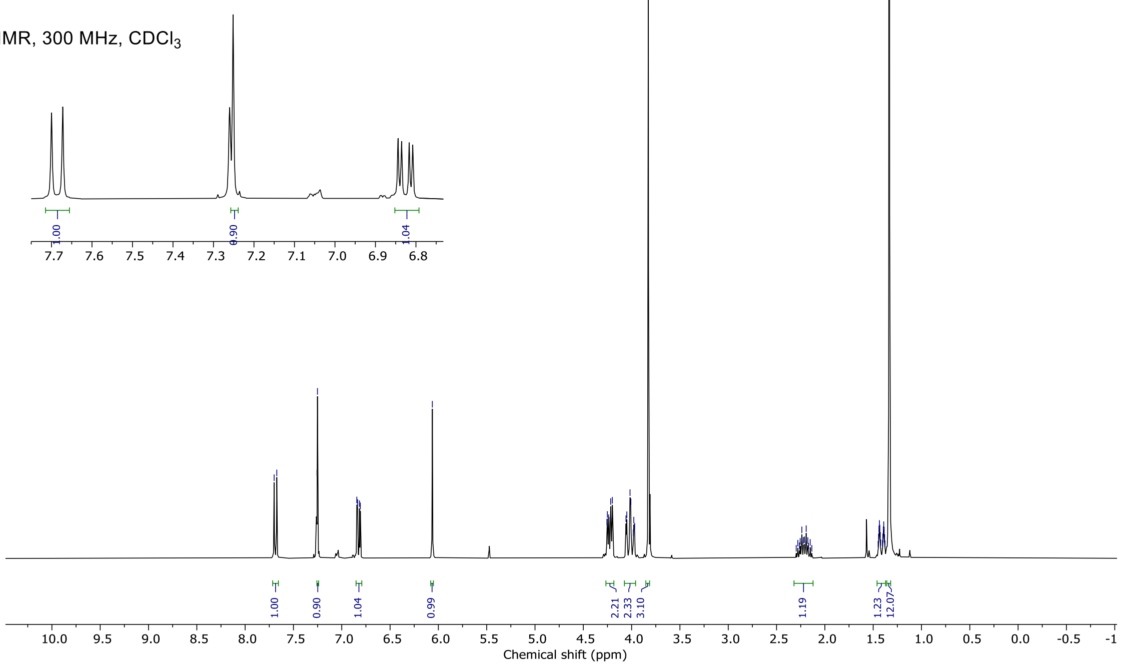

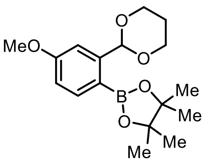

^{13}C NMR, 75 MHz, $CDCl_3$

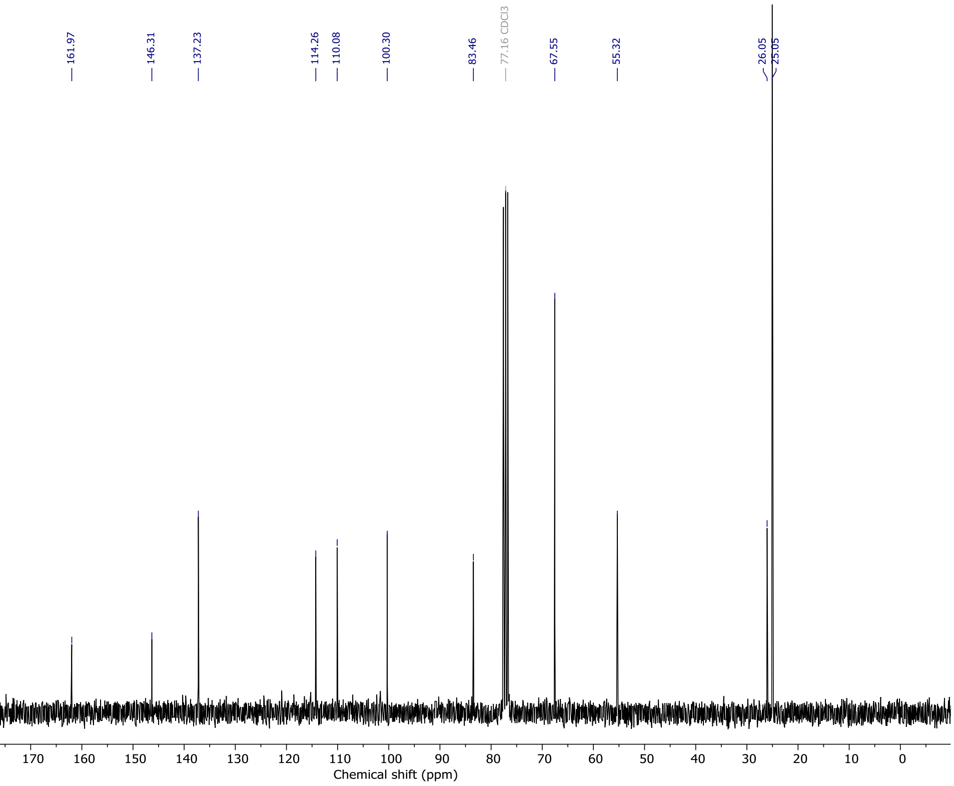

Compound 8

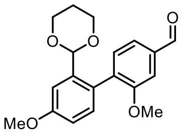

^{1}H NMR, 300 MHz, $CDCl_3$

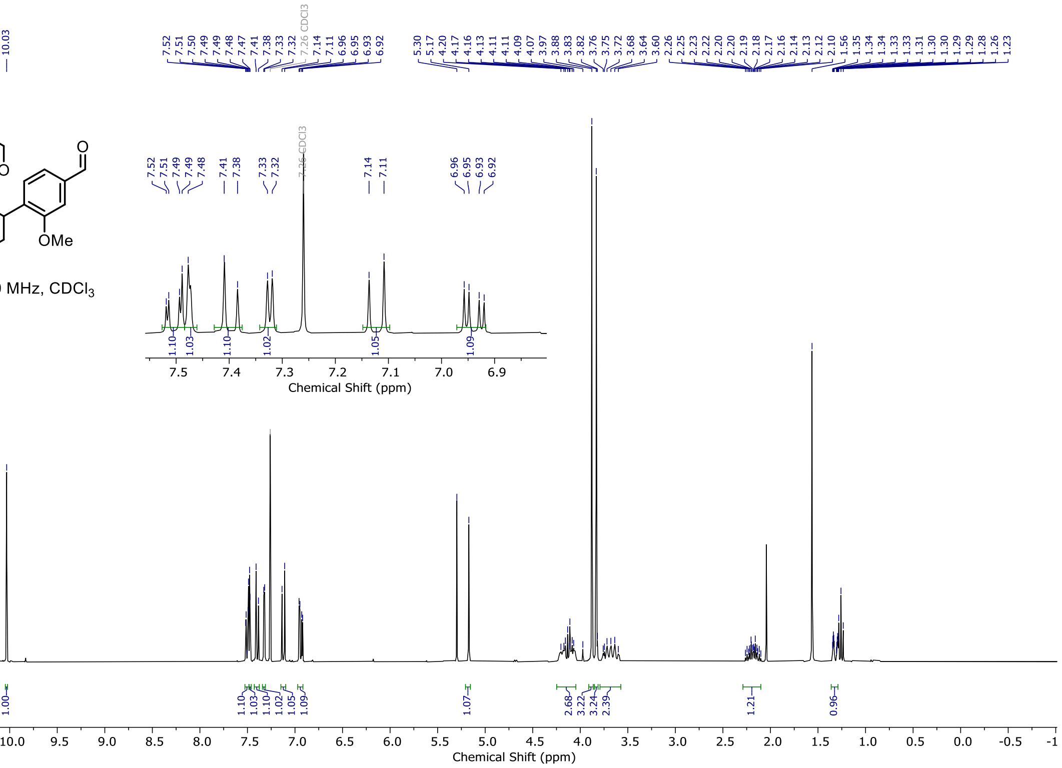

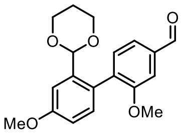

^{13}C NMR, 101 MHz, $CDCl_3$

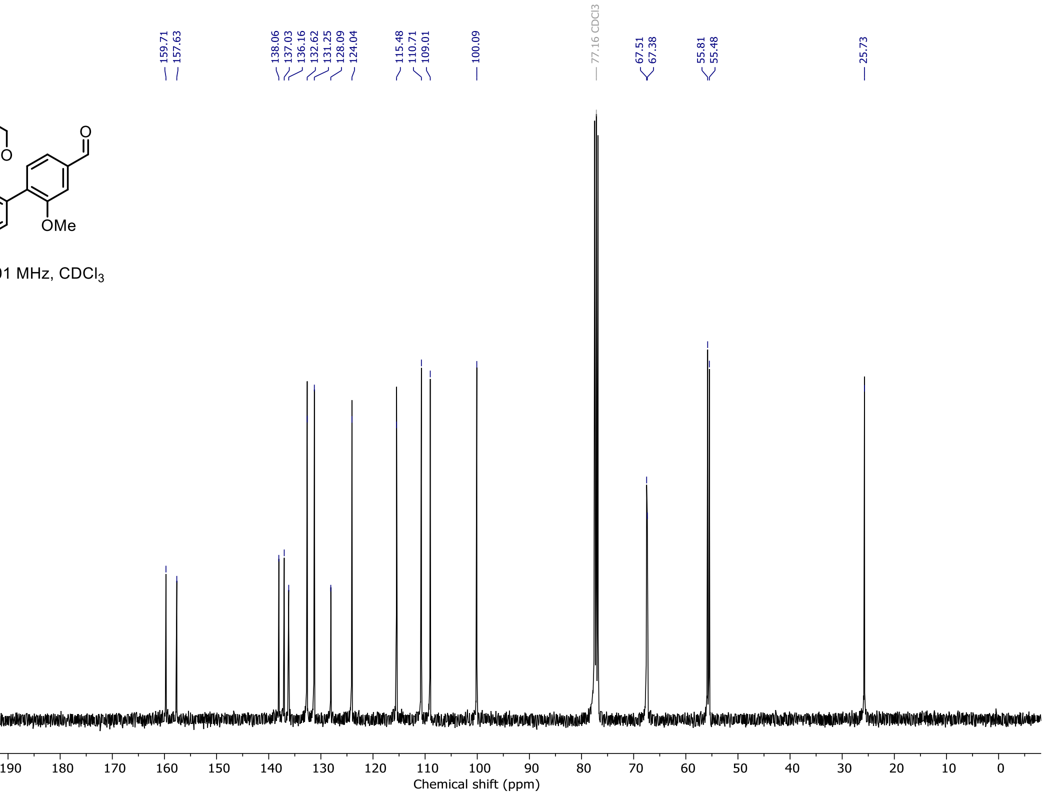

Compound 9

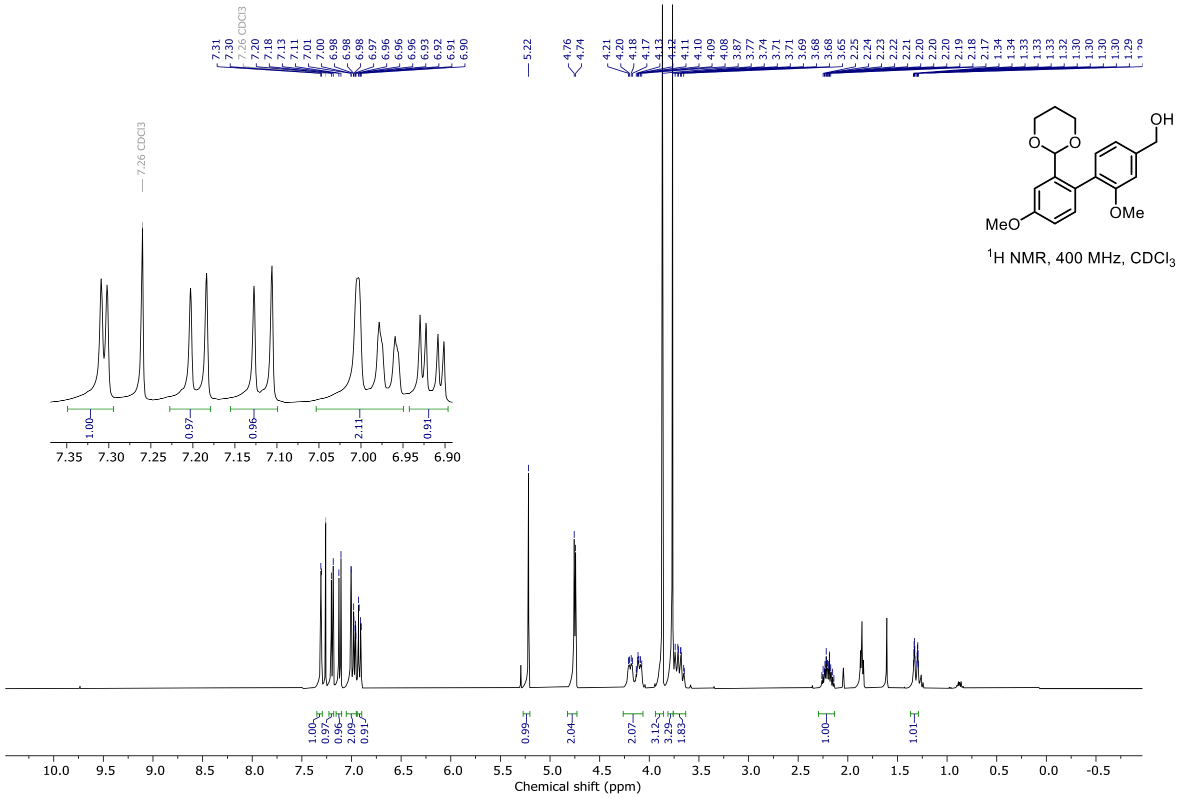
OH
O
O
MeO
OMe
^{1}H NMR, 400 MHz, CDCl$_3$
7.26 CDCl3
Chemical shift (ppm)

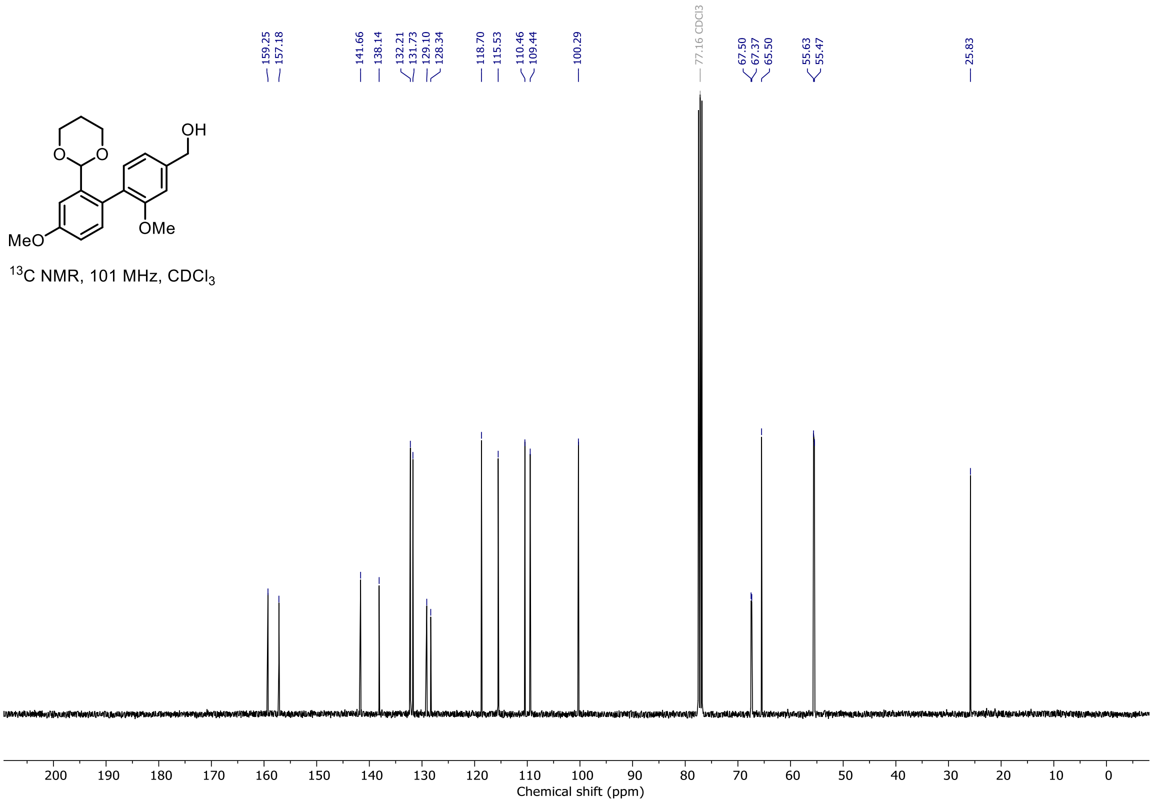
159.25
157.18
141.66
138.14
132.21
131.73
129.10
128.34
118.70
115.53
110.46
109.44
100.29
77.16 CDCl3
67.50
67.37
65.50
55.63
55.47
25.83
OH
O
O
MeO
OMe
^{13}C NMR, 101 MHz, CDCl$_3$
Chemical shift (ppm)

Compound 10

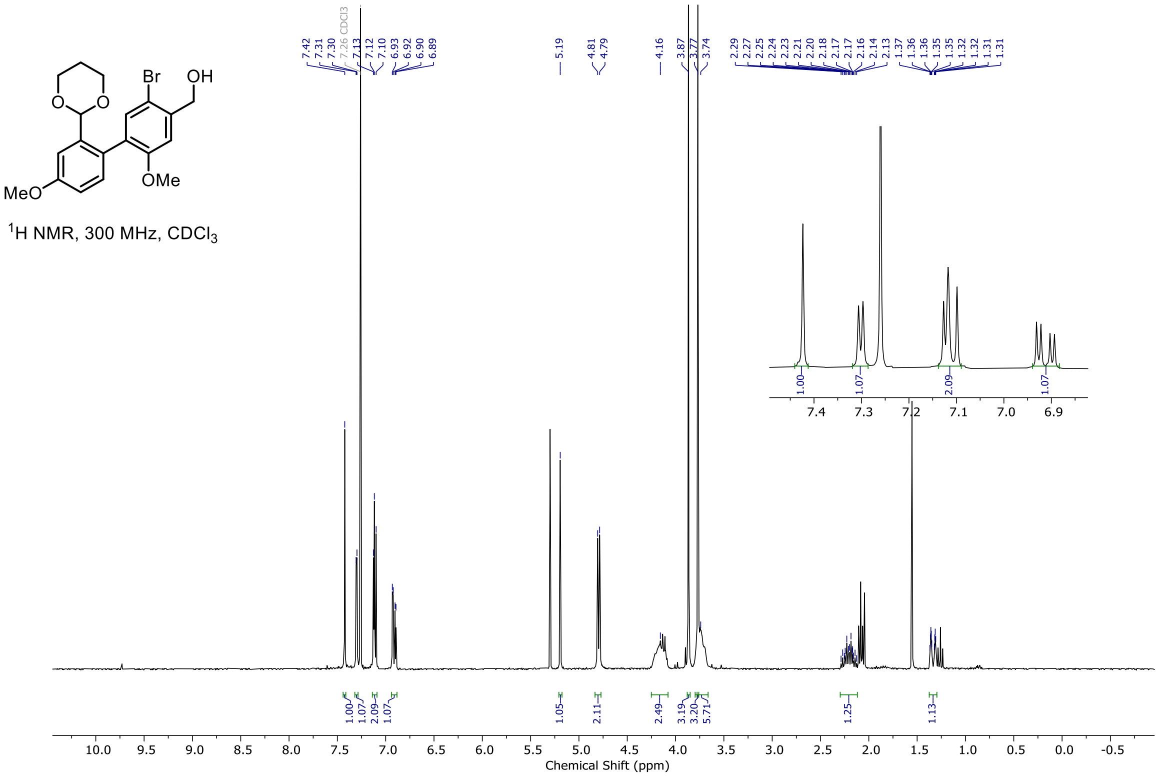

Br
OH
O
O
MeO
OMe
^{1}H NMR, 300 MHz, CDCl3
7.42
7.31
7.30
7.26 CDCl3
7.13
7.12
7.10
6.93
6.92
6.90
6.89
5.19
4.81
4.79
4.16
3.87
3.77
3.74
2.29
2.27
2.25
2.24
2.23
2.21
2.20
2.18
2.17
2.17
2.16
2.14
2.13
1.37
1.36
1.36
1.35
1.35
1.32
1.32
1.31
1.31
1.00
1.07
2.09
1.07
1.05
2.11
2.49
3.19
3.20
5.71
1.25
1.13
Chemical Shift (ppm)

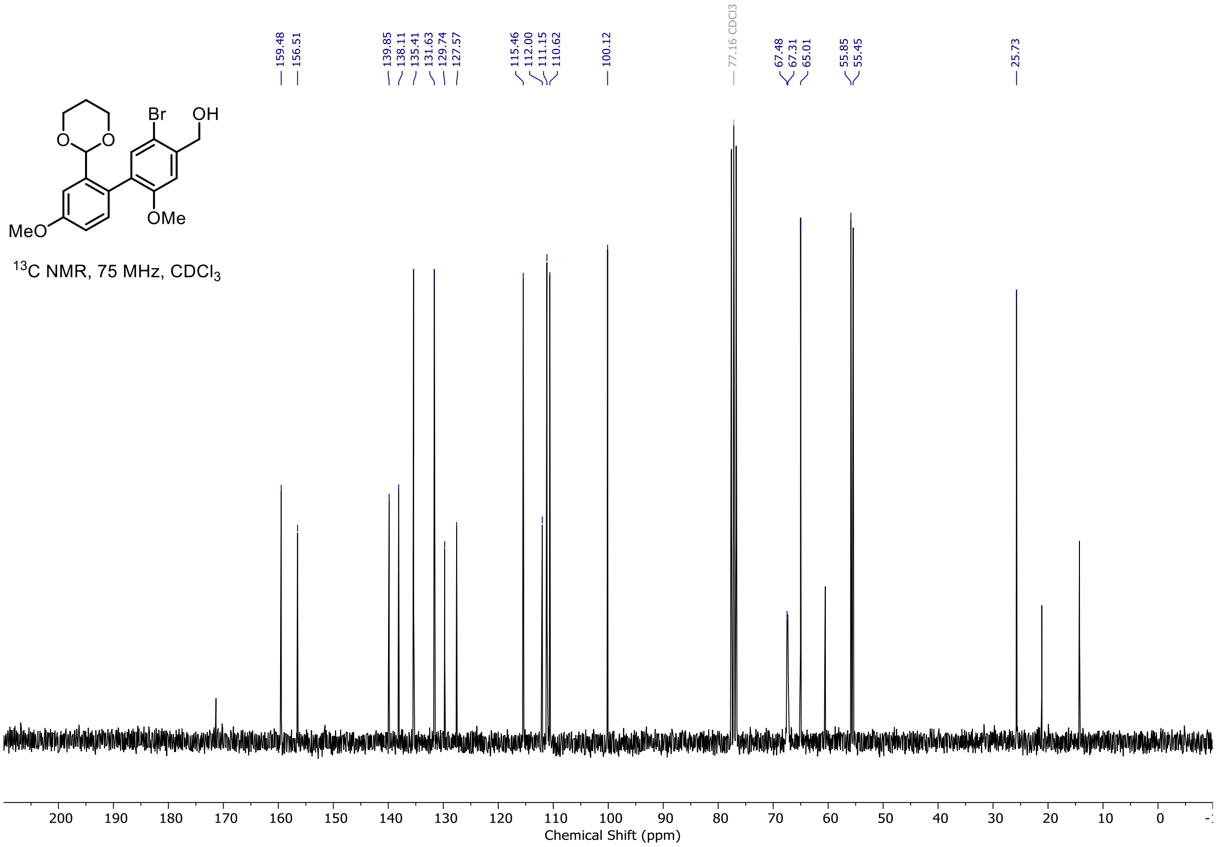

Br
OH
O
O
MeO
OMe
^{13}C NMR, 75 MHz, CDCl3
159.48
156.51
139.85
138.11
135.41
131.63
129.74
127.57
115.46
112.00
111.15
110.62
100.12
77.16 CDCl3
67.48
67.31
65.01
55.85
55.45
25.73
Chemical Shift (ppm)

Compound 11

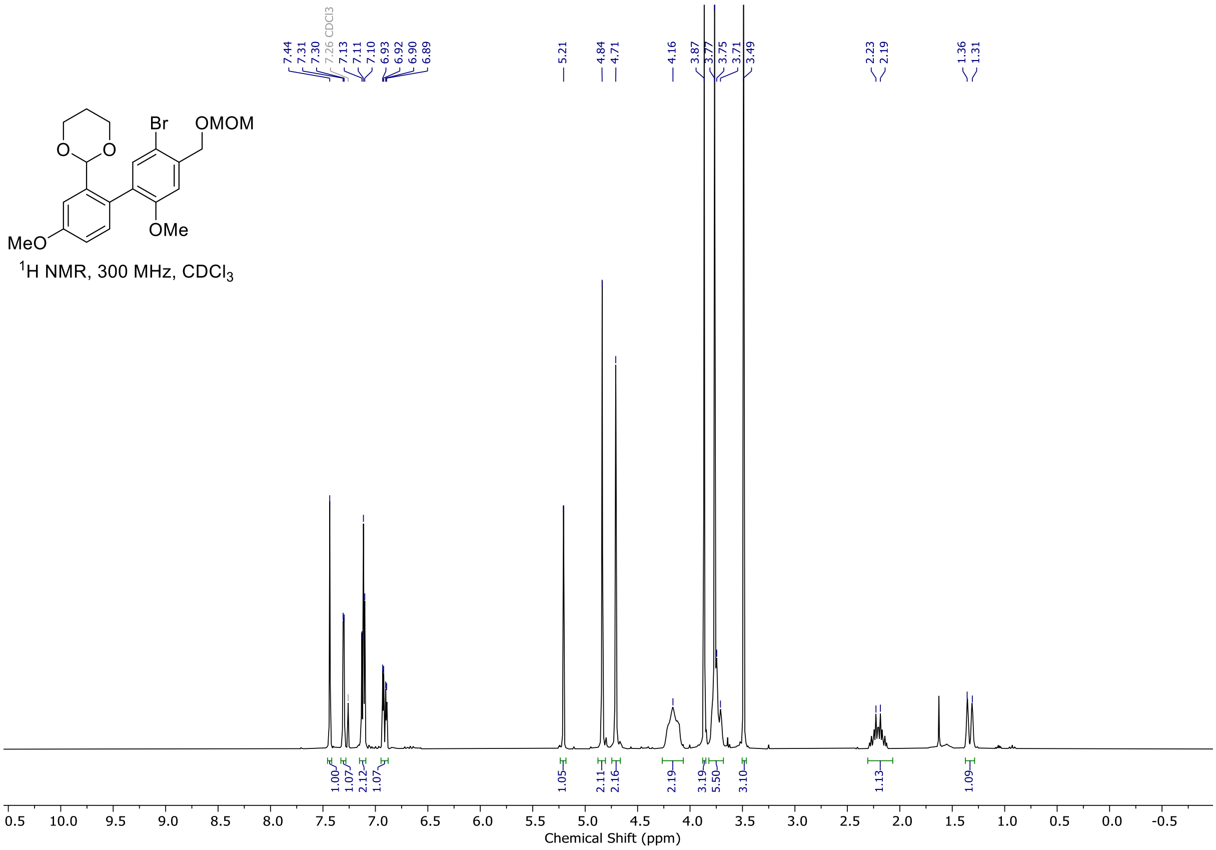

^{1}H NMR, 300 MHz, $CDCl_3$

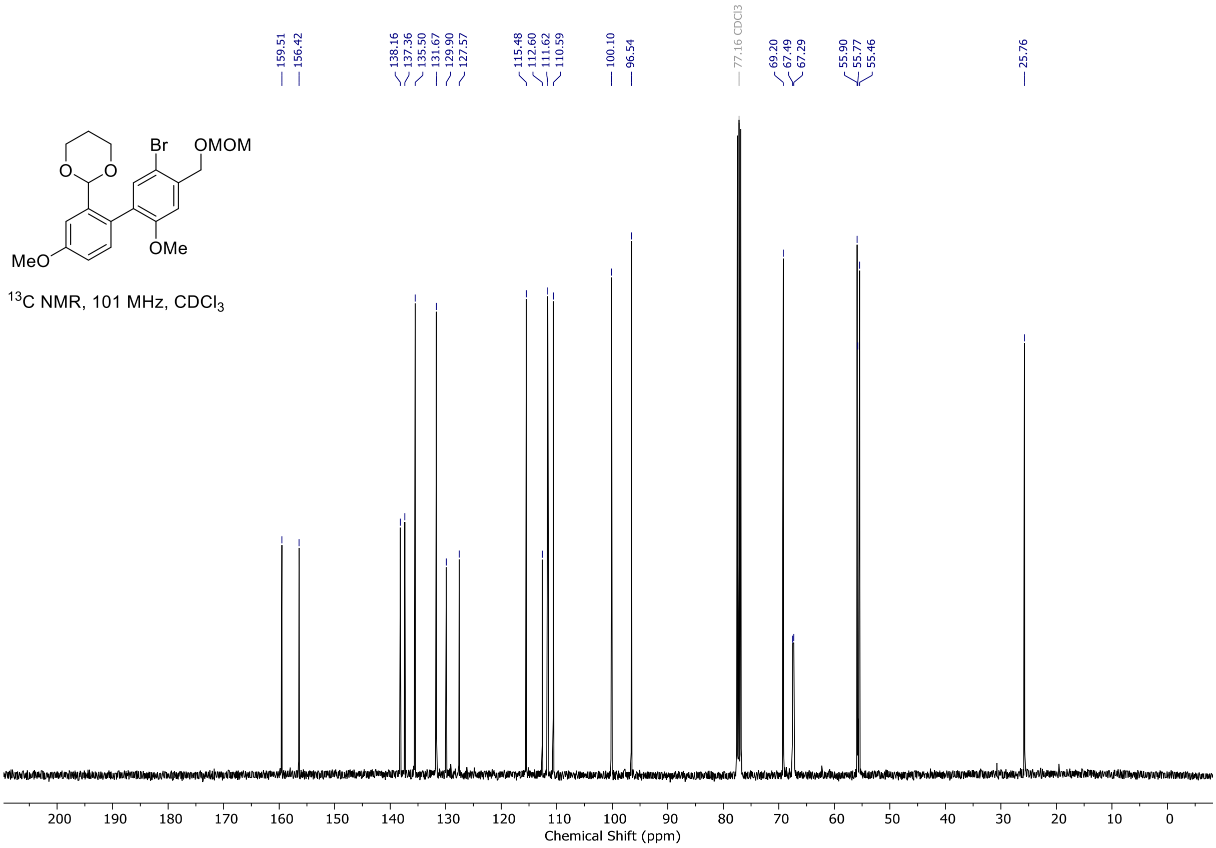

^{13}C NMR, 101 MHz, $CDCl_3$

Compound 12

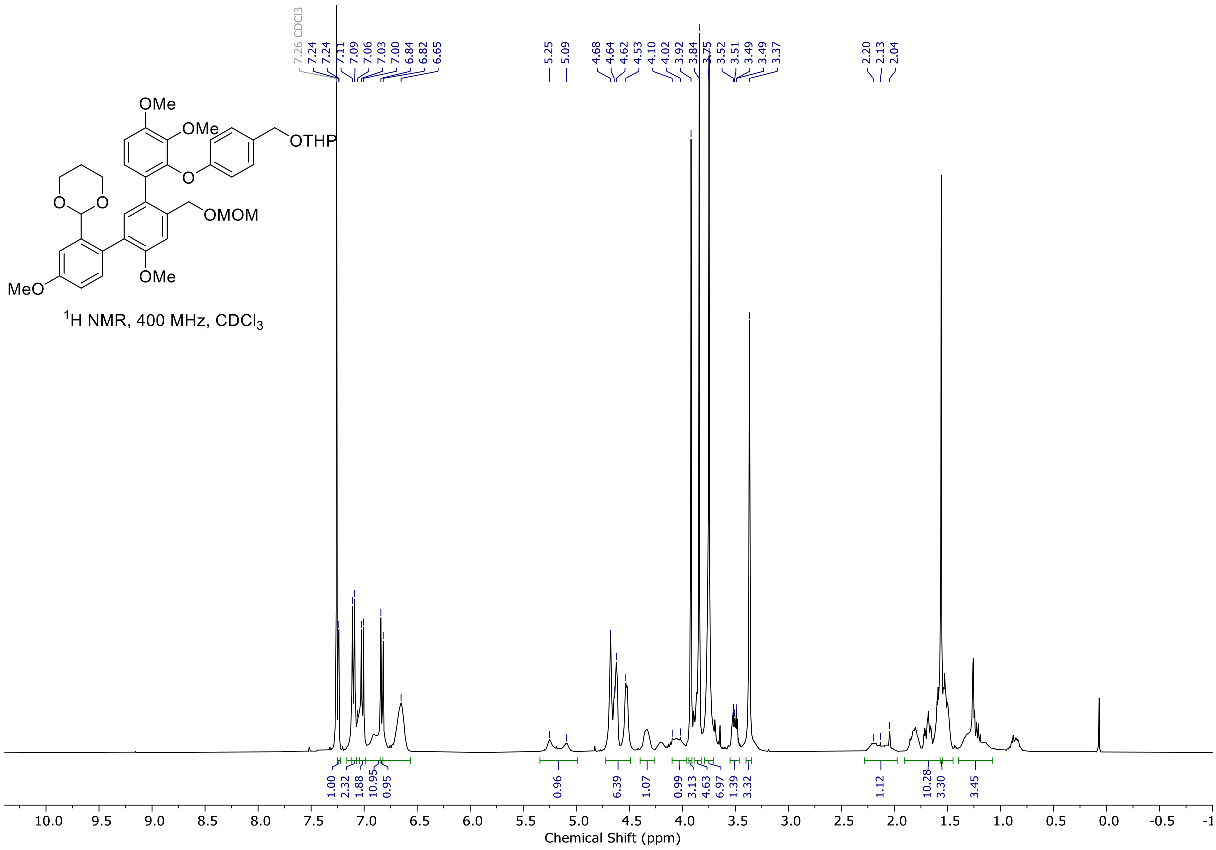
OMe
OMe
OTHP
O
O O
OMOM
MeO
OMe
^{1}H NMR, 400 MHz, $CDCl_3$
7.26 CDCl3
7.24 7.24 7.11 7.09 7.06 7.03 7.00 6.84 6.82 6.65
5.25 5.09
4.68 4.64 4.62 4.53 4.10 4.02 3.92 3.84 3.75 3.52 3.51 3.49 3.49 3.37
2.20 2.13 2.04
1.00 2.32 1.88 10.95 0.95 0.96 6.39 1.07 0.99 3.13 4.63 6.97 1.39 3.32 1.12 10.28 3.30 3.45
Chemical Shift (ppm)

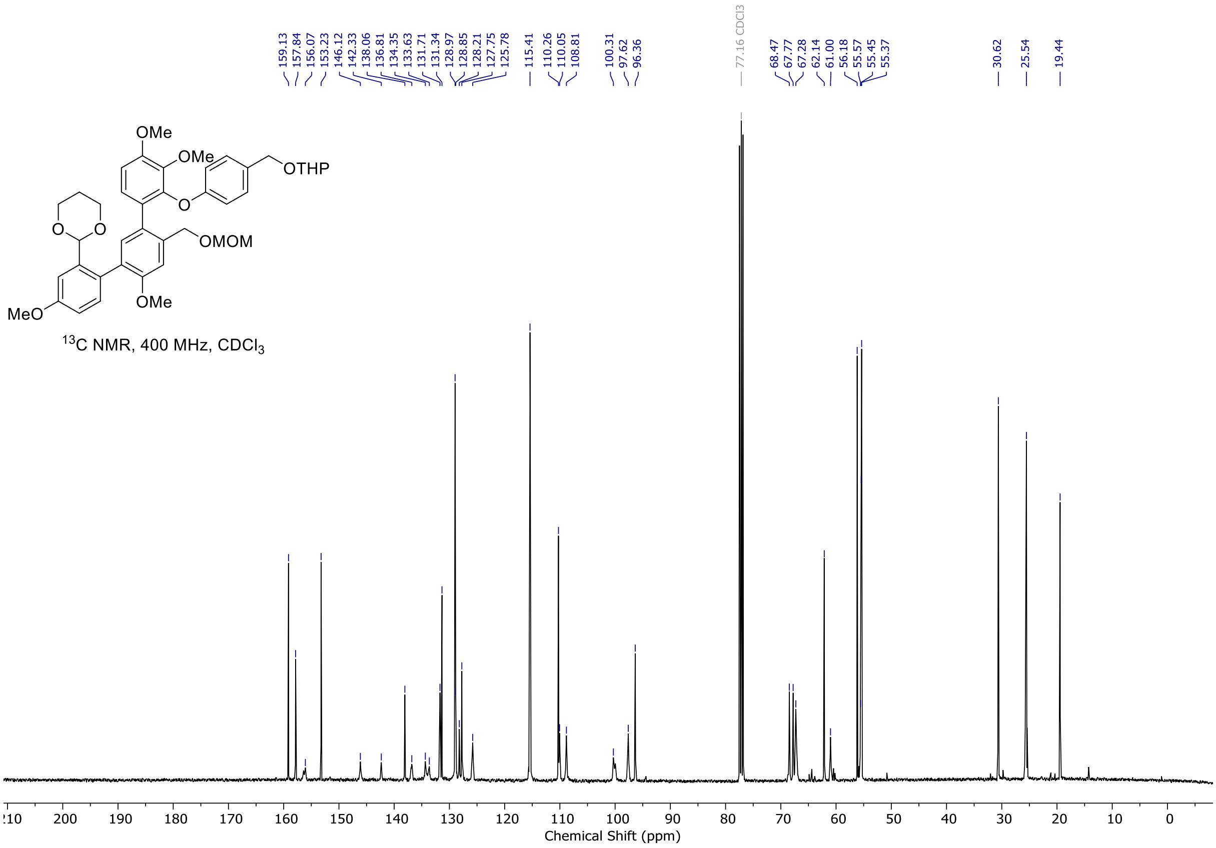
OMe
OMe
OTHP
O
O O
OMOM
MeO
OMe
^{13}C NMR, 400 MHz, $CDCl_3$
159.13 157.84 156.07 153.23 146.12 142.33 138.06 136.81 134.35 133.63 131.71 131.34 128.97 128.85 128.21 127.75 125.78 115.41 110.26 110.05 108.81 100.31 97.62 96.36
77.16 CDCl3
68.47 67.77 67.28 62.14 61.00 56.18 55.57 55.45 55.37 30.62 25.54 19.44
Chemical Shift (ppm)

Compound 13

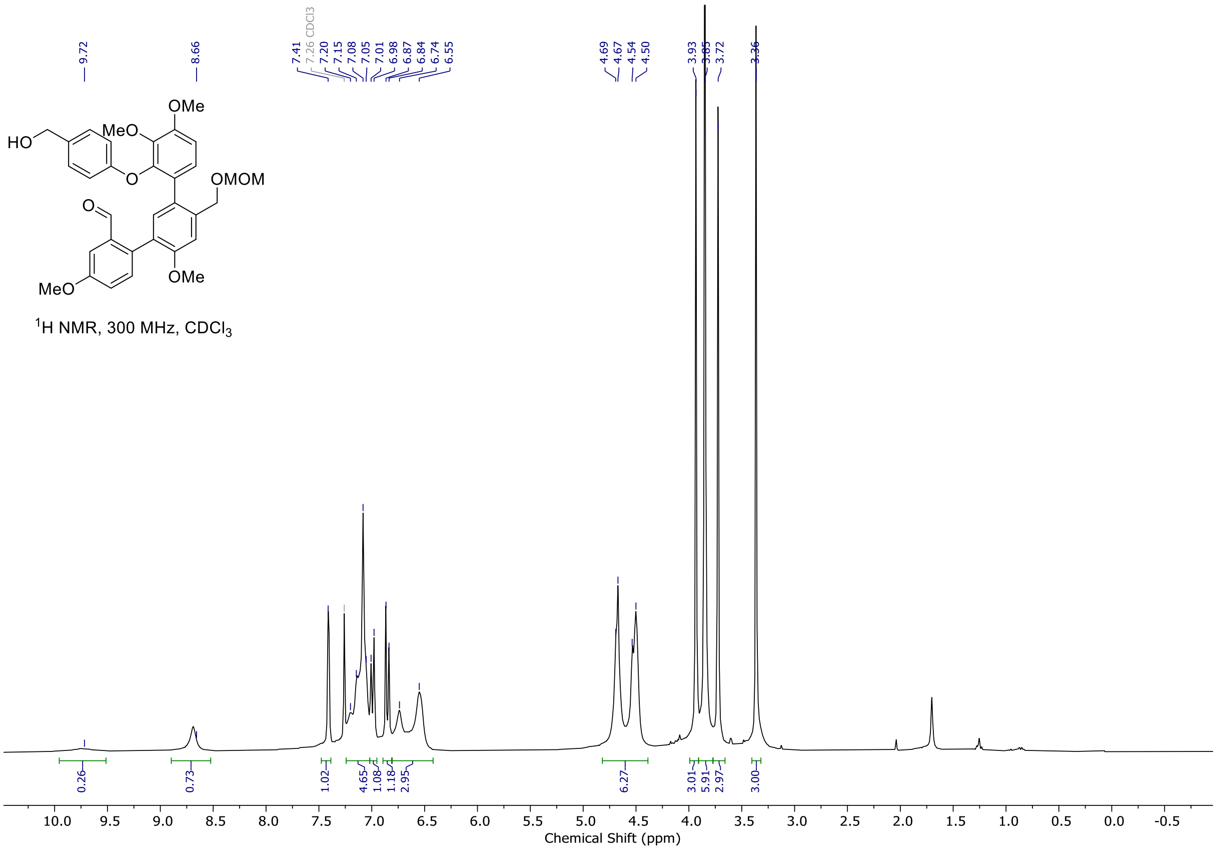

^1H NMR, 300 MHz, $CDCl_3$

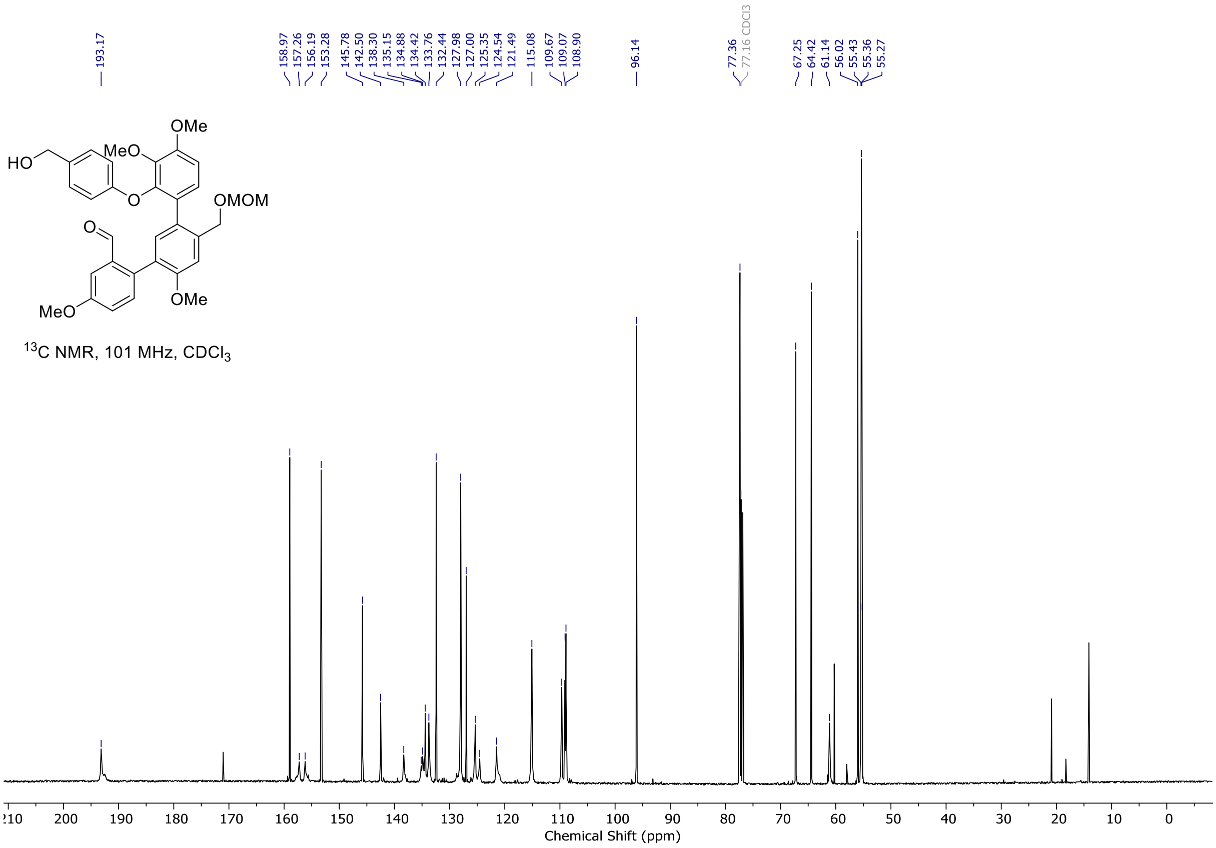

^{13}C NMR, 101 MHz, $CDCl_3$

Compound 14

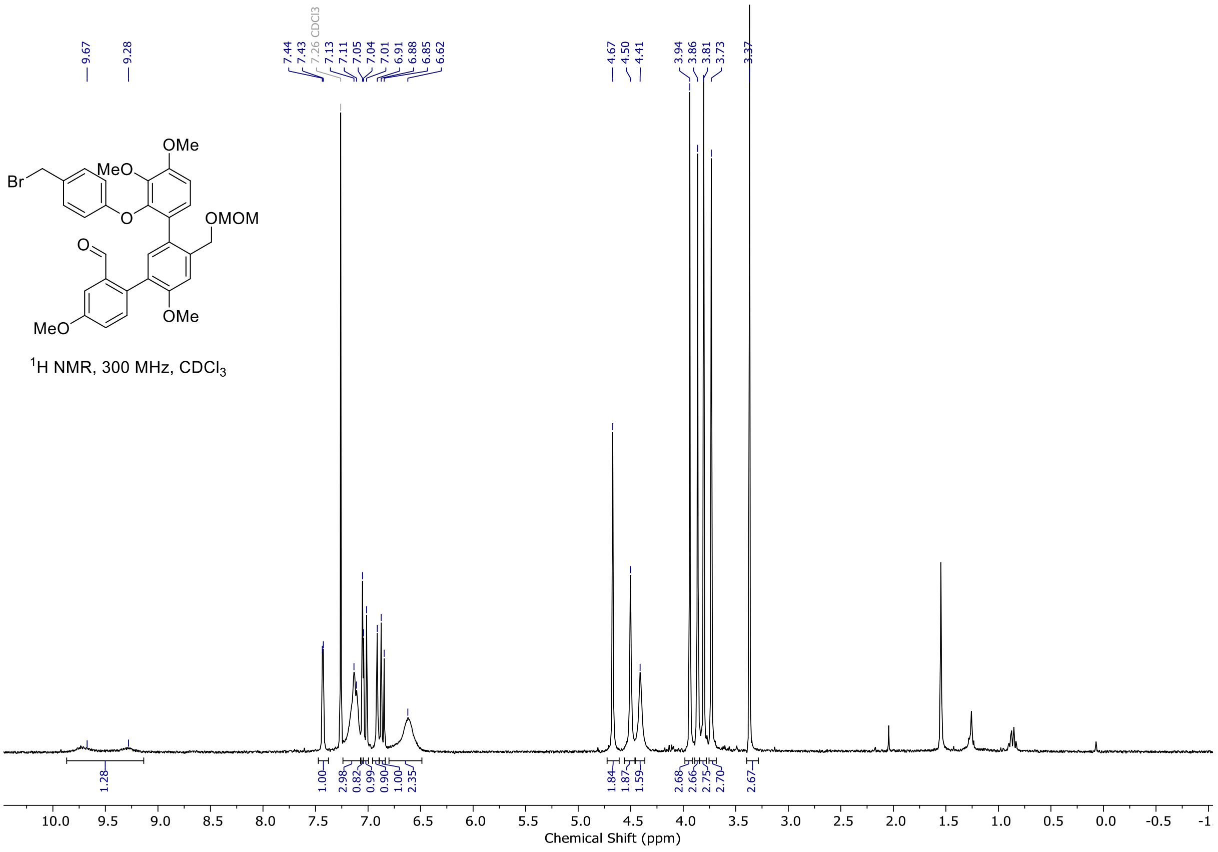

OMe
MeO
Br
O
OMOM
O
MeO
OMe
^{1}H NMR, 300 MHz, $CDCl_3$
9.67
9.28
7.44
7.43
7.26 CDCl3
7.13
7.11
7.05
7.04
7.01
6.91
6.88
6.85
6.62
4.67
4.50
4.41
3.94
3.86
3.81
3.73
3.37
1.28
1.00
2.98
0.82
0.99
0.90
1.00
2.35
1.84
1.87
1.59
2.68
2.66
2.75
2.70
2.67
Chemical Shift (ppm)

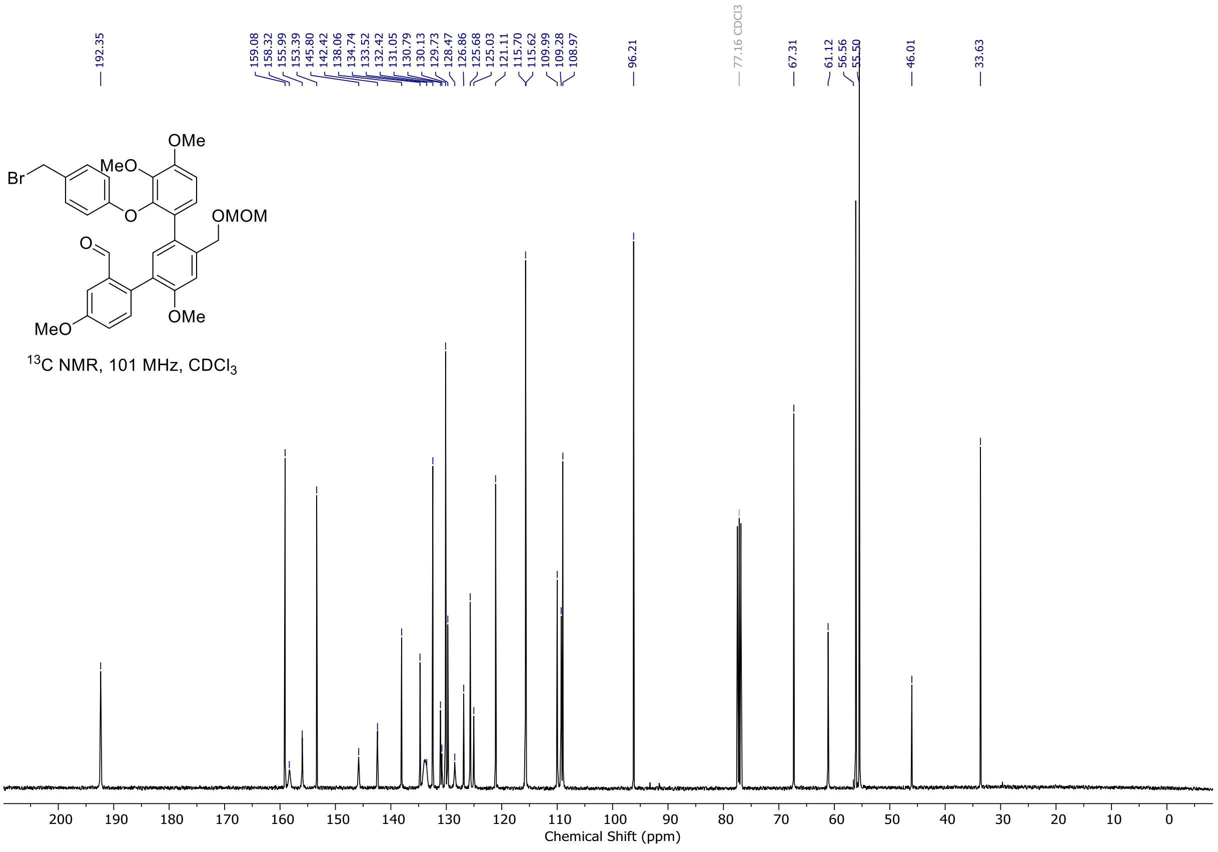

OMe
MeO
Br
O
OMOM
O
MeO
OMe
^{13}C NMR, 101 MHz, $CDCl_3$
192.35
159.08
158.32
155.99
153.39
145.80
142.42
138.06
134.74
133.52
132.42
131.05
130.79
130.13
129.73
128.47
126.86
125.68
125.03
121.11
115.70
115.62
109.99
109.28
108.97
96.21
77.16 CDCl3
67.31
61.12
56.56
55.50
46.01
33.63
Chemical Shift (ppm)

Compound 15

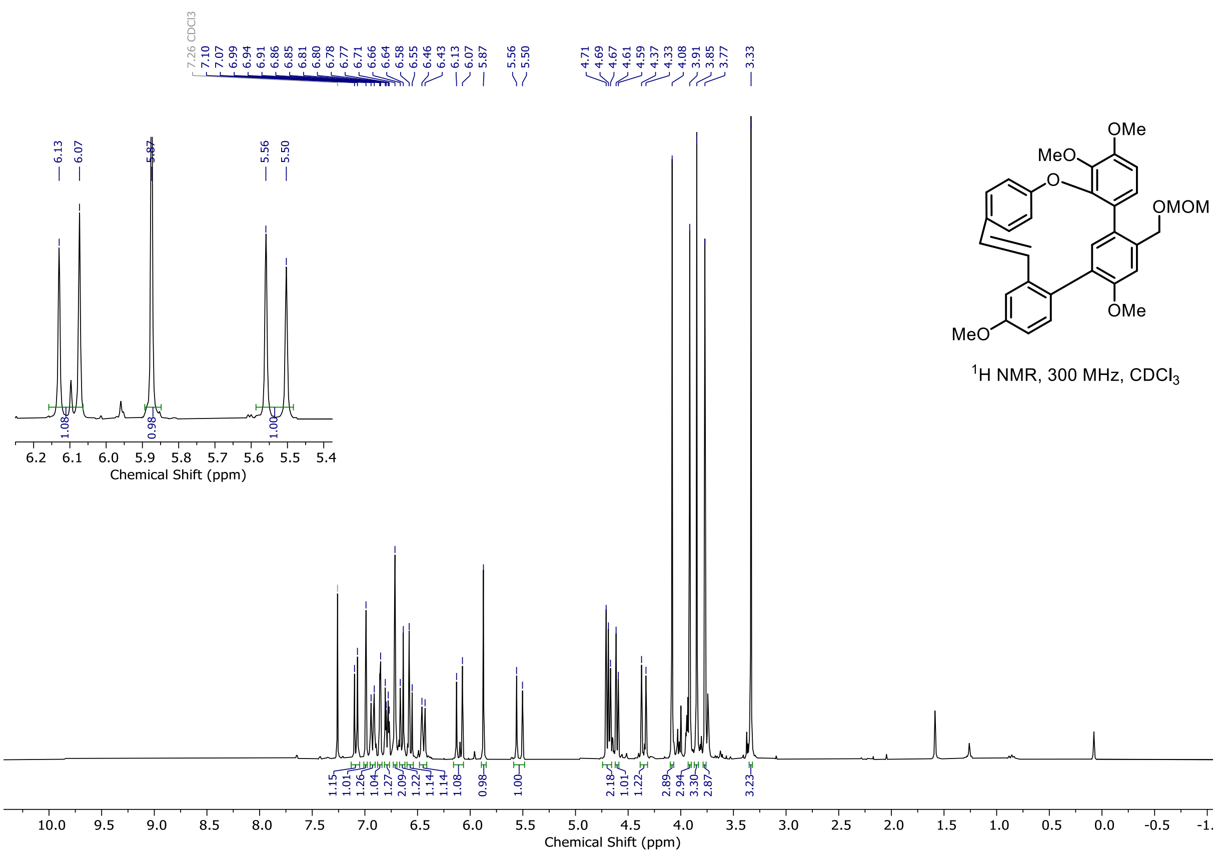

^{1}H NMR, 300 MHz, $CDCl_3$

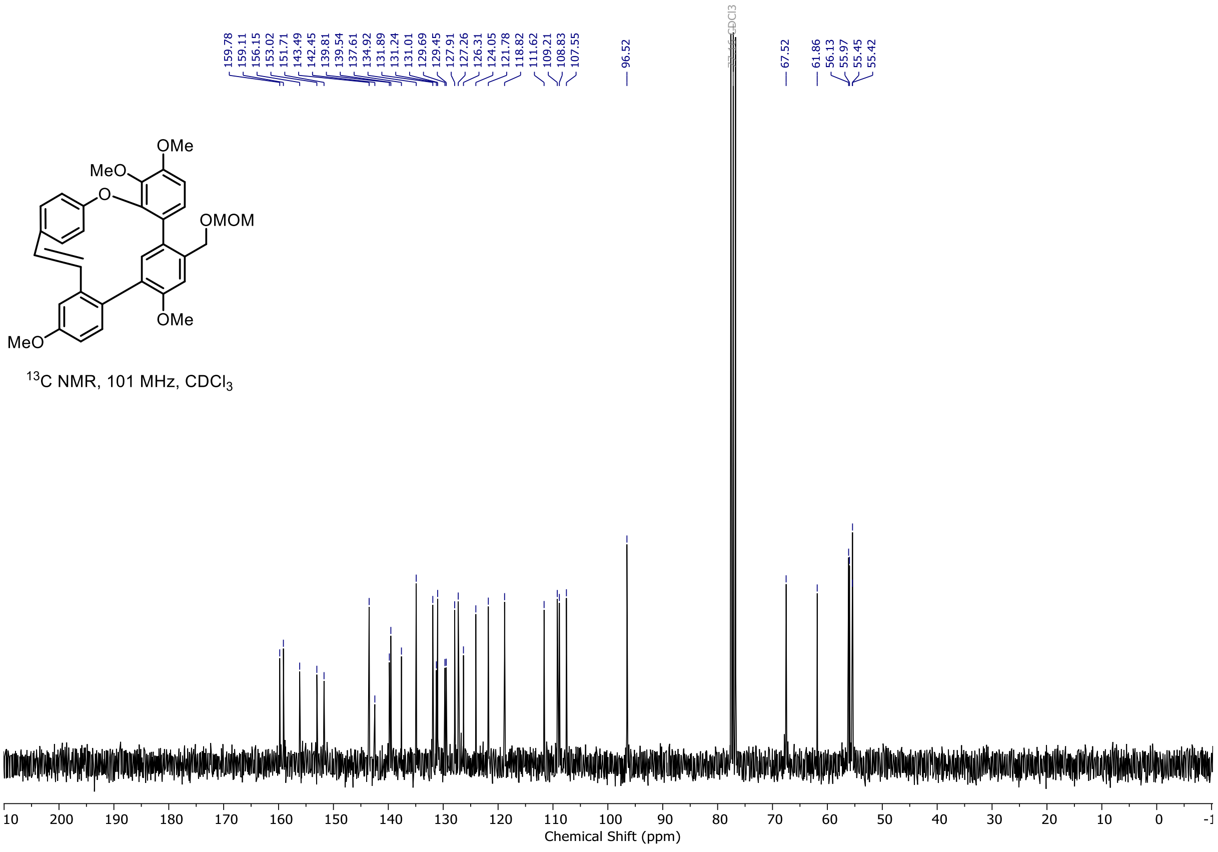

^{13}C NMR, 101 MHz, $CDCl_3$

Compound 16

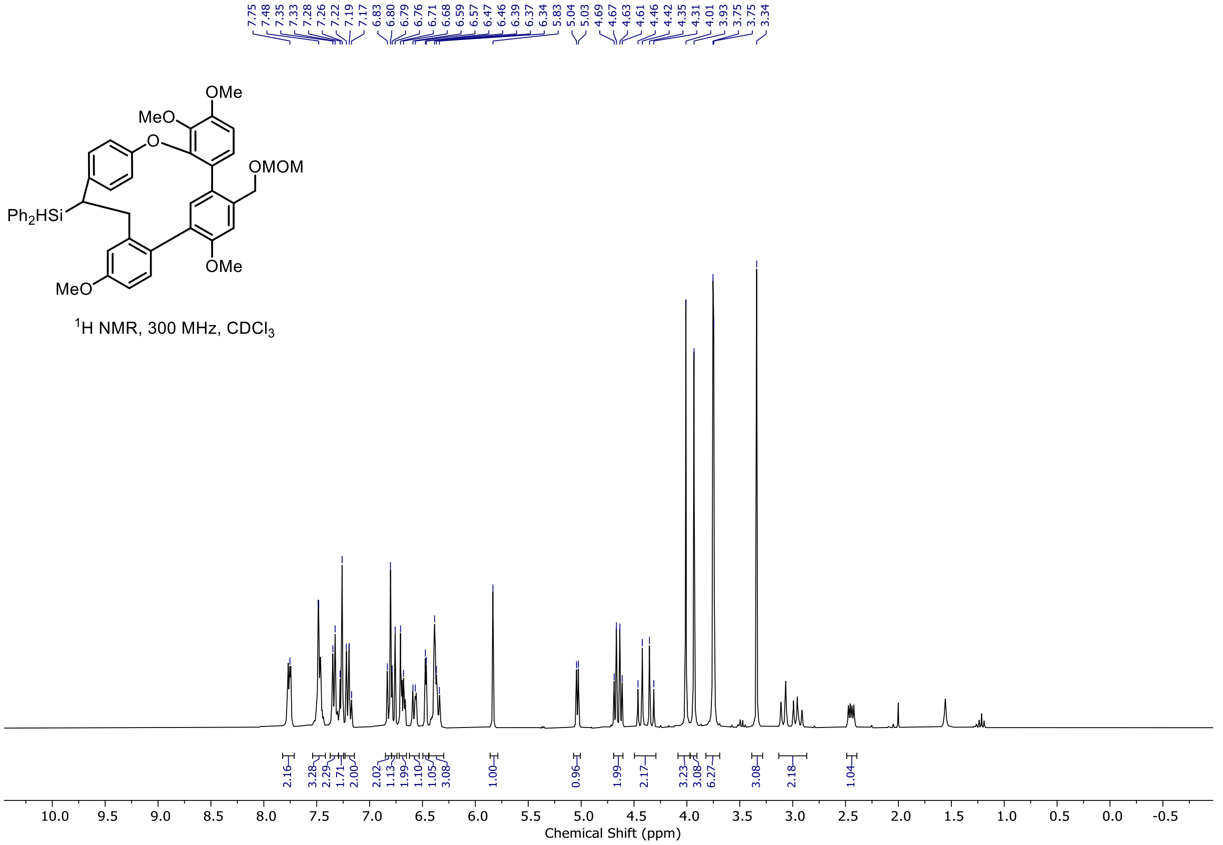

^{1}H NMR, 300 MHz, $CDCl_3$

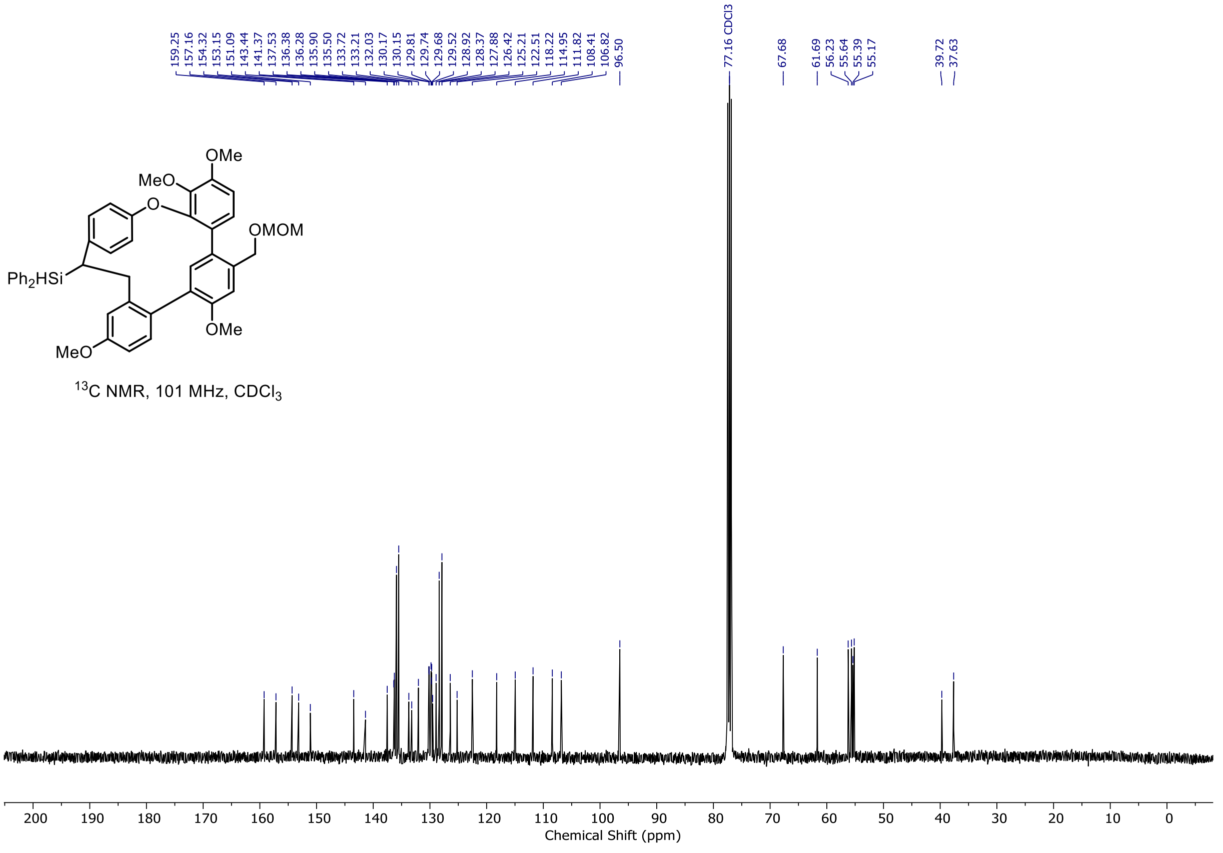

^{13}C NMR, 101 MHz, $CDCl_3$

Compound 17

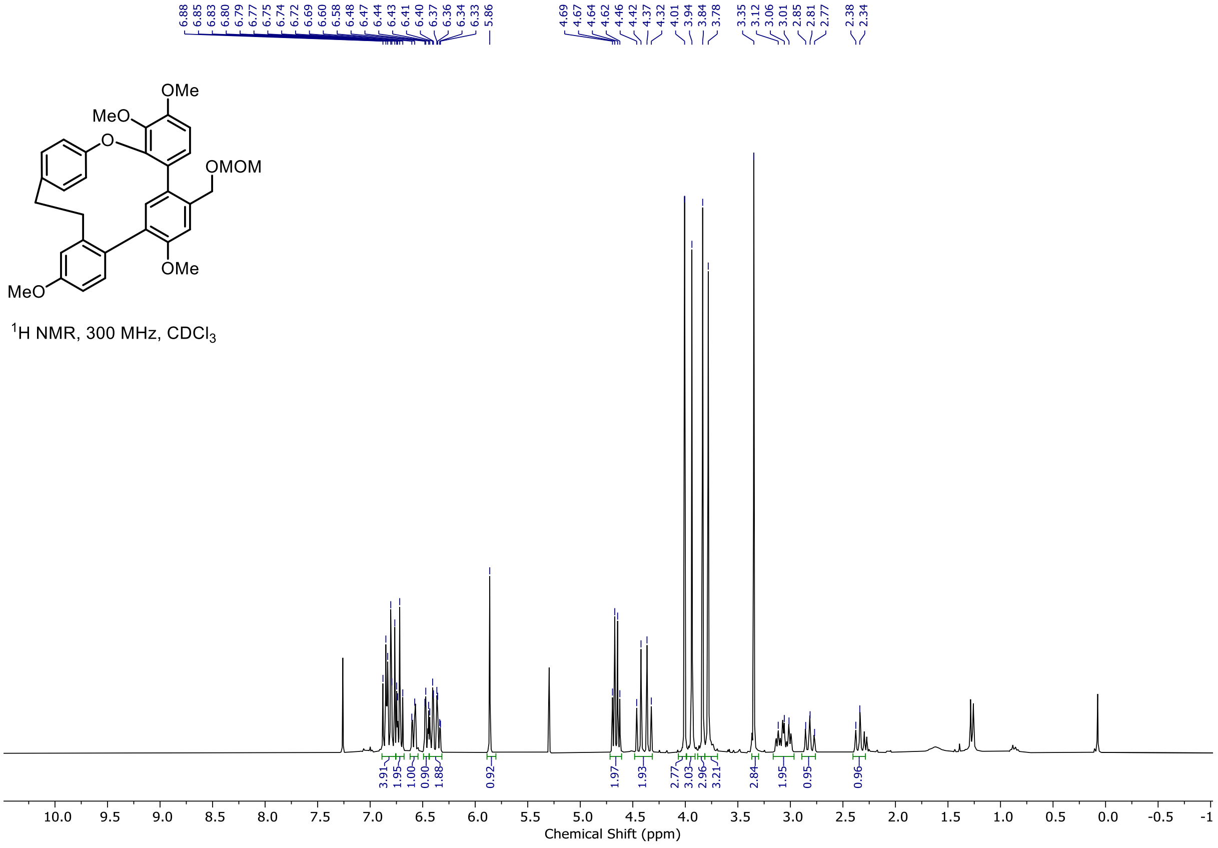

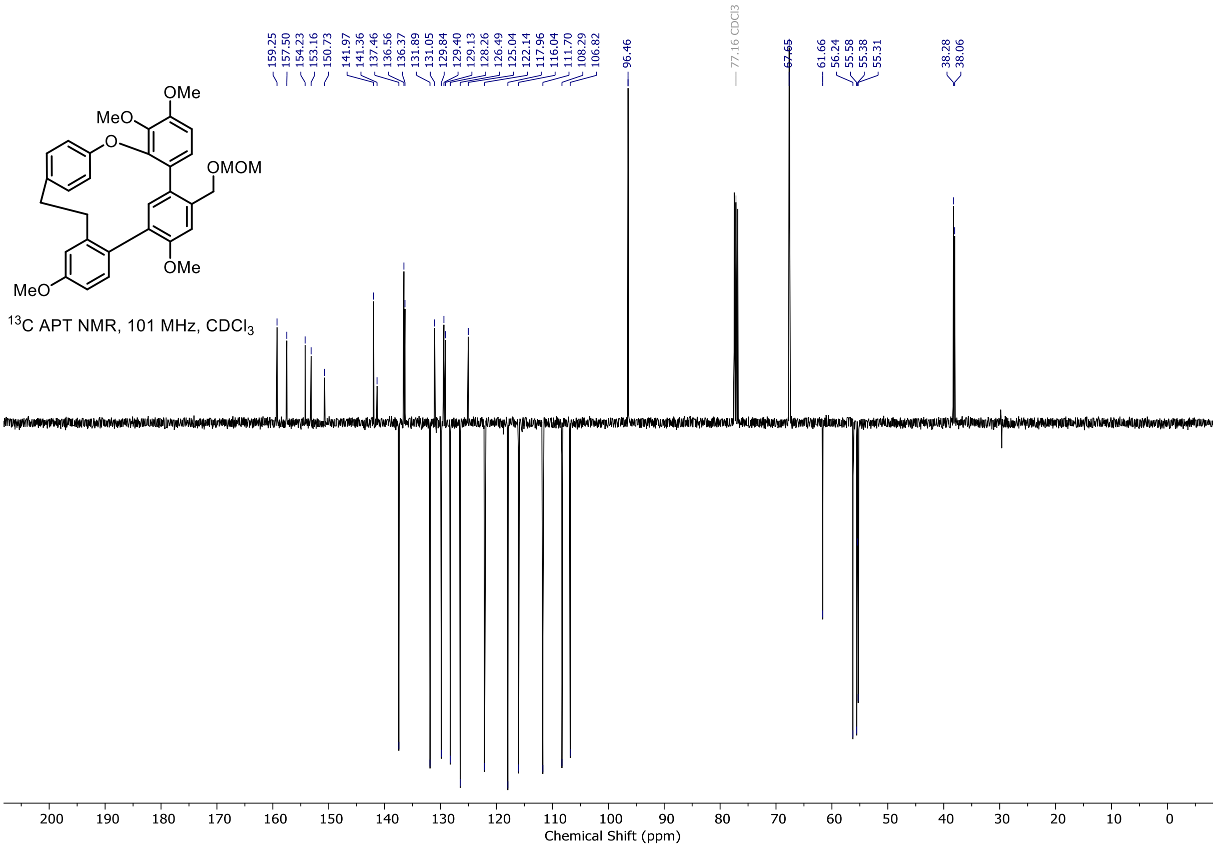

Compound 18

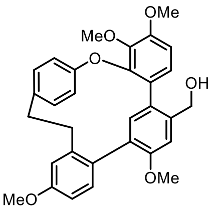

^{1}H NMR, 300 MHz, $CDCl_3$

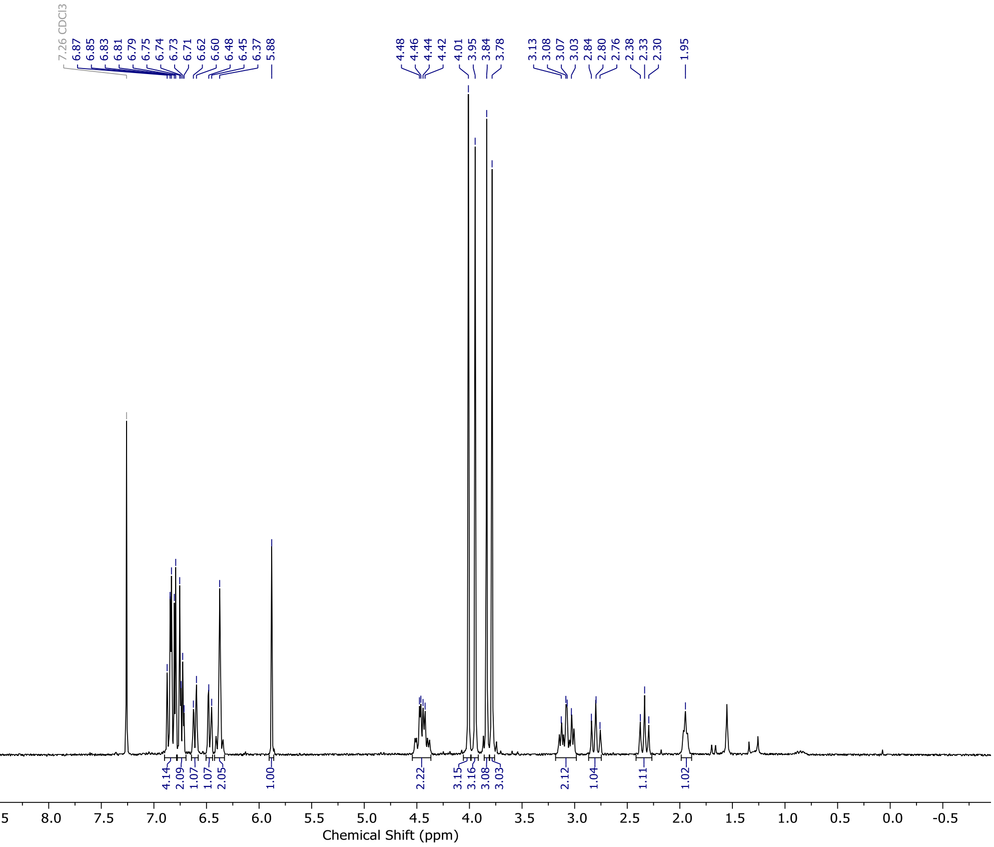

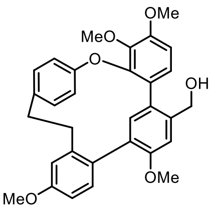

^{13}C NMR, 101 MHz, $CDCl_3$

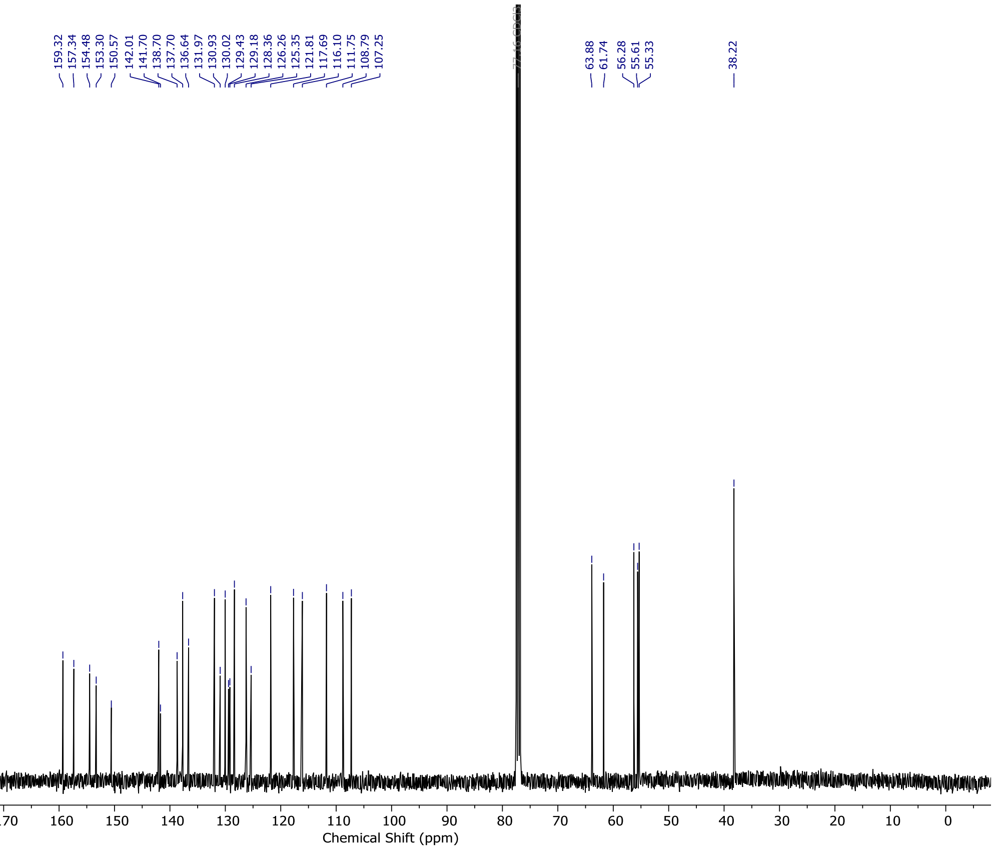

Compound 19

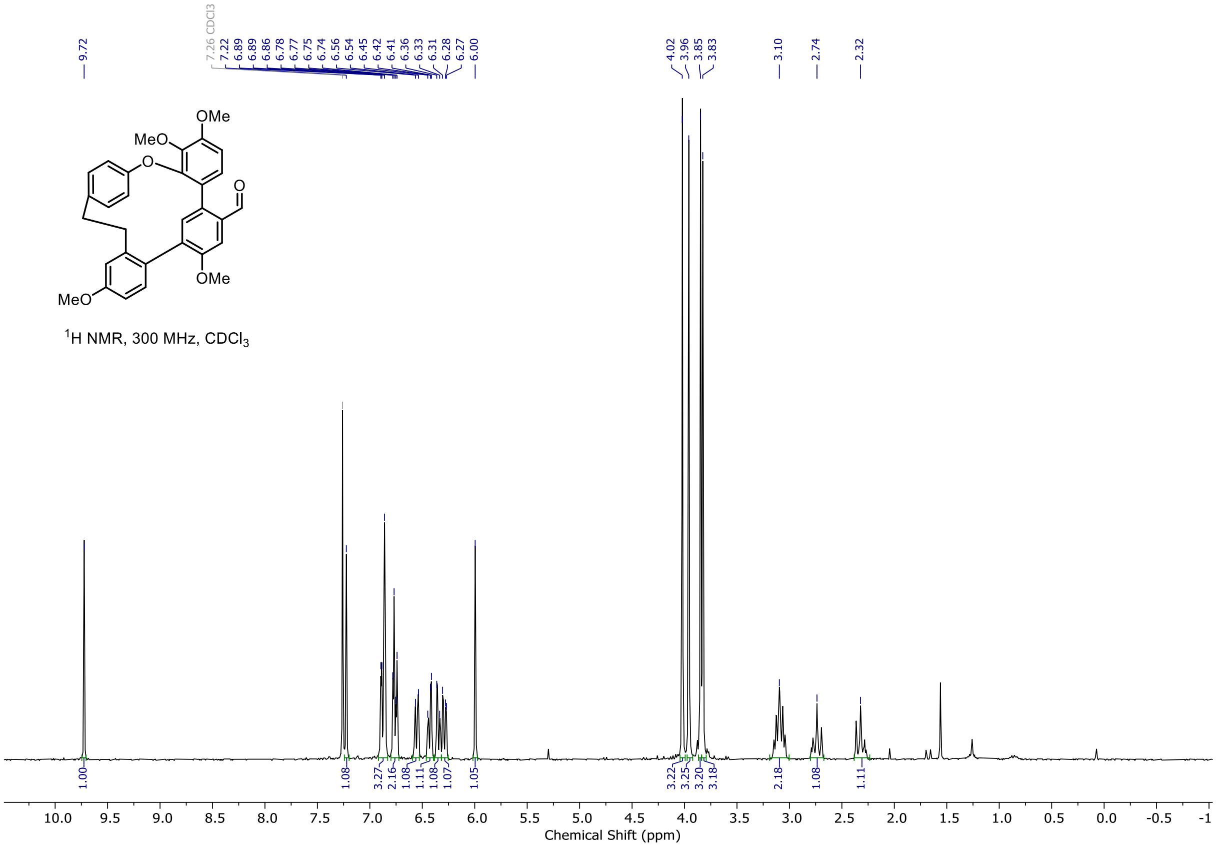
9.72
7.26 CDCl3
7.22
6.89
6.89
6.86
6.78
6.77
6.75
6.74
6.56
6.54
6.45
6.42
6.41
6.36
6.33
6.31
6.28
6.27
6.00
4.02
3.96
3.85
3.83
3.10
2.74
2.32
OMe
MeO
O
O
OMe
MeO
1H NMR, 300 MHz, CDCl3
1.00
1.08
3.27
2.16
1.08
1.11
1.08
1.07
1.05
3.22
3.25
3.20
3.18
2.18
1.08
1.11
Chemical Shift (ppm)

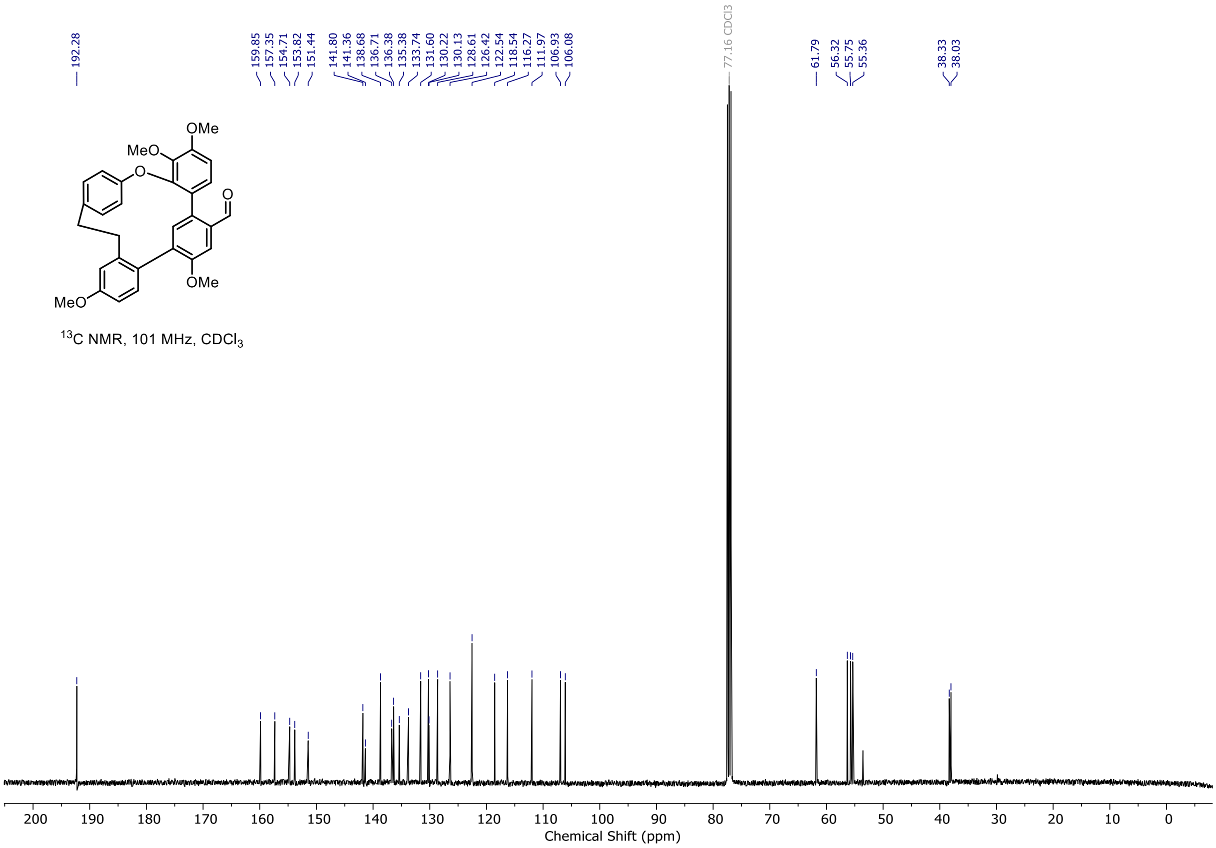
192.28
159.85
157.35
154.71
153.82
151.44
141.80
141.36
138.68
136.71
136.38
135.38
133.74
131.60
130.22
130.13
128.61
126.42
122.54
118.54
116.27
111.97
106.93
106.08
77.16 CDCl3
61.79
56.32
55.75
55.36
38.33
38.03
OMe
MeO
O
O
OMe
MeO
13C NMR, 101 MHz, CDCl3
Chemical Shift (ppm)

Compound 20

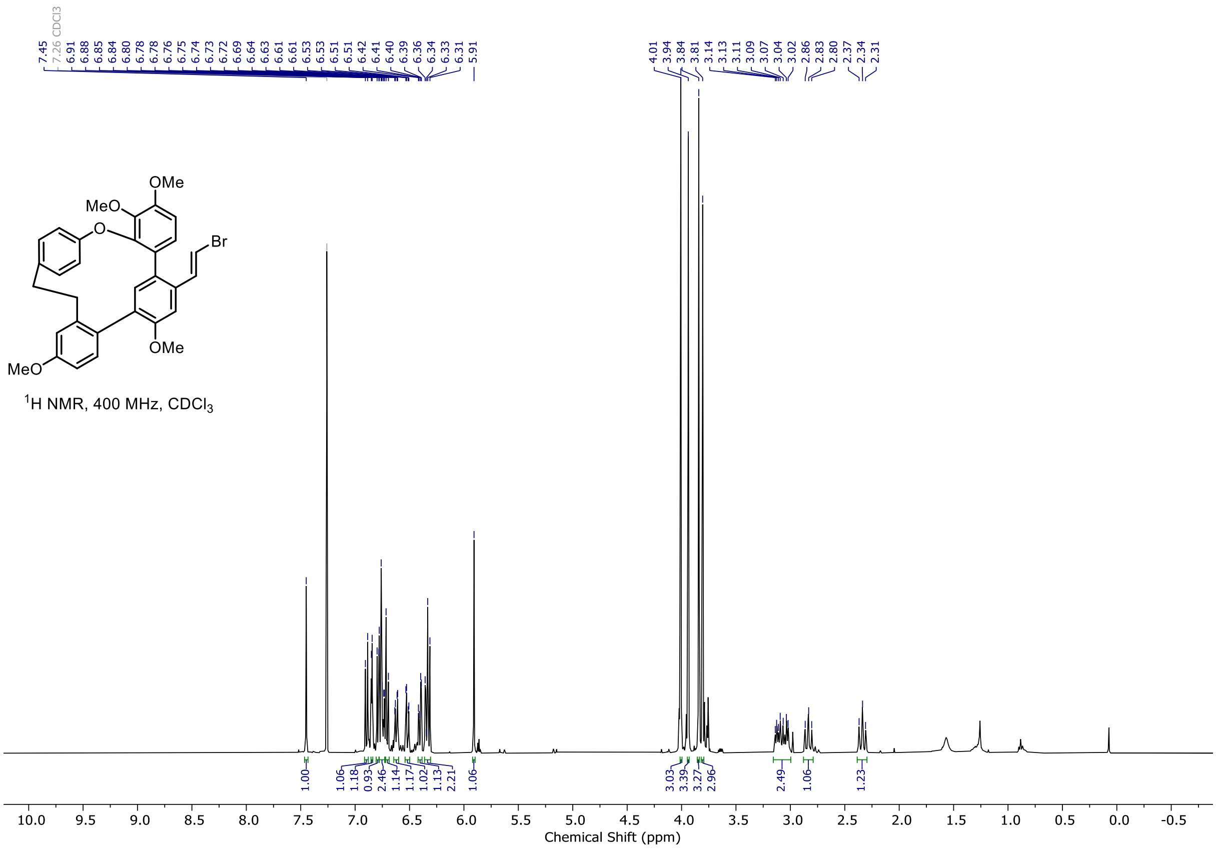

^{1}H NMR, 400 MHz, $CDCl_3$

0

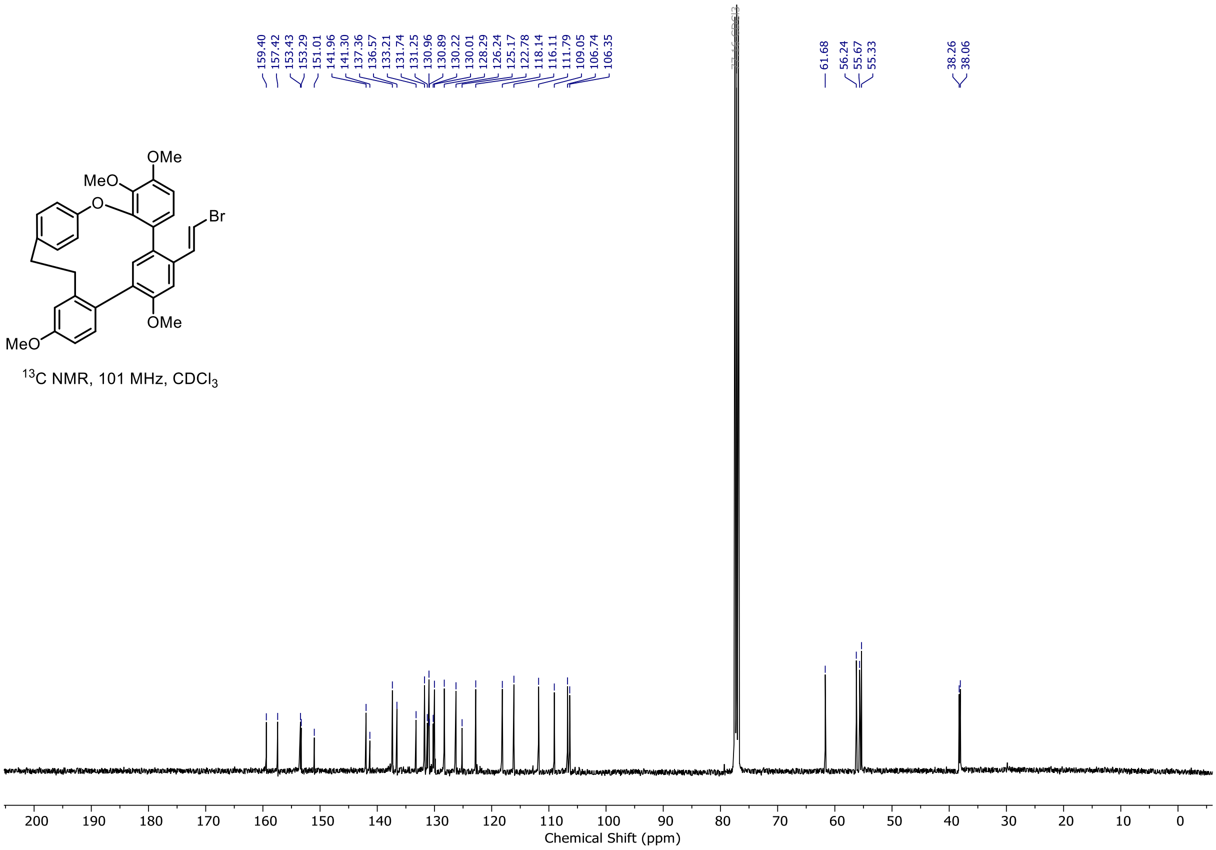

^{13}C NMR, 101 MHz, $CDCl_3$

Compound 21

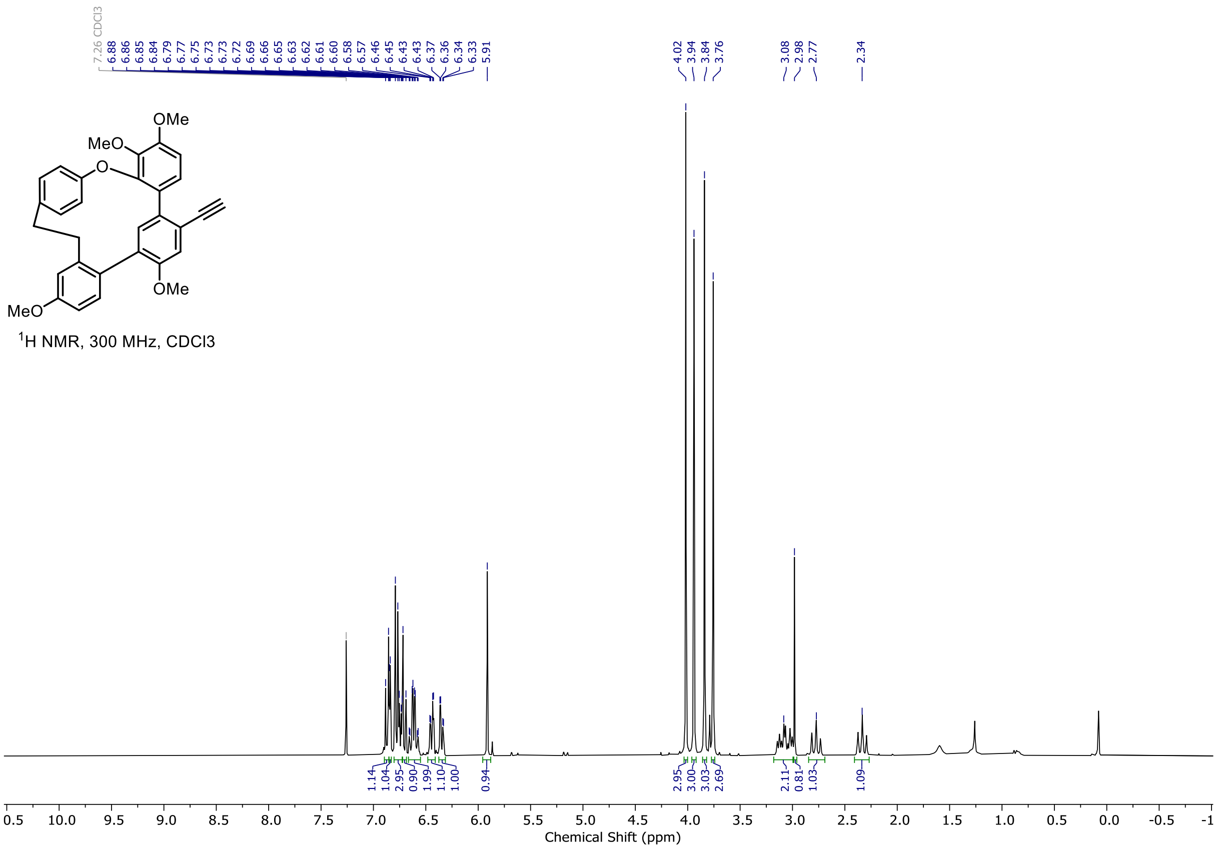

^{1}H NMR, 300 MHz, CDCl3

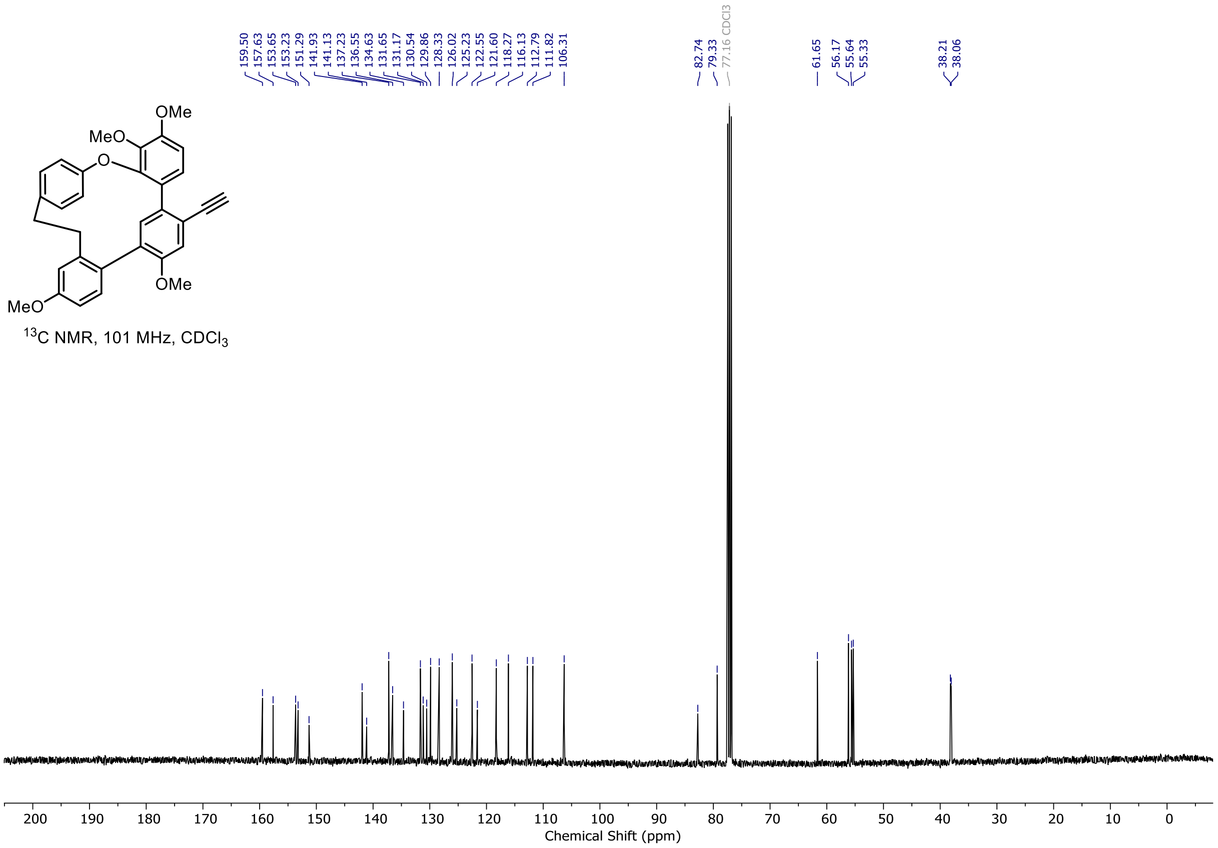

^{13}C NMR, 101 MHz, $CDCl_3$

Compound 24

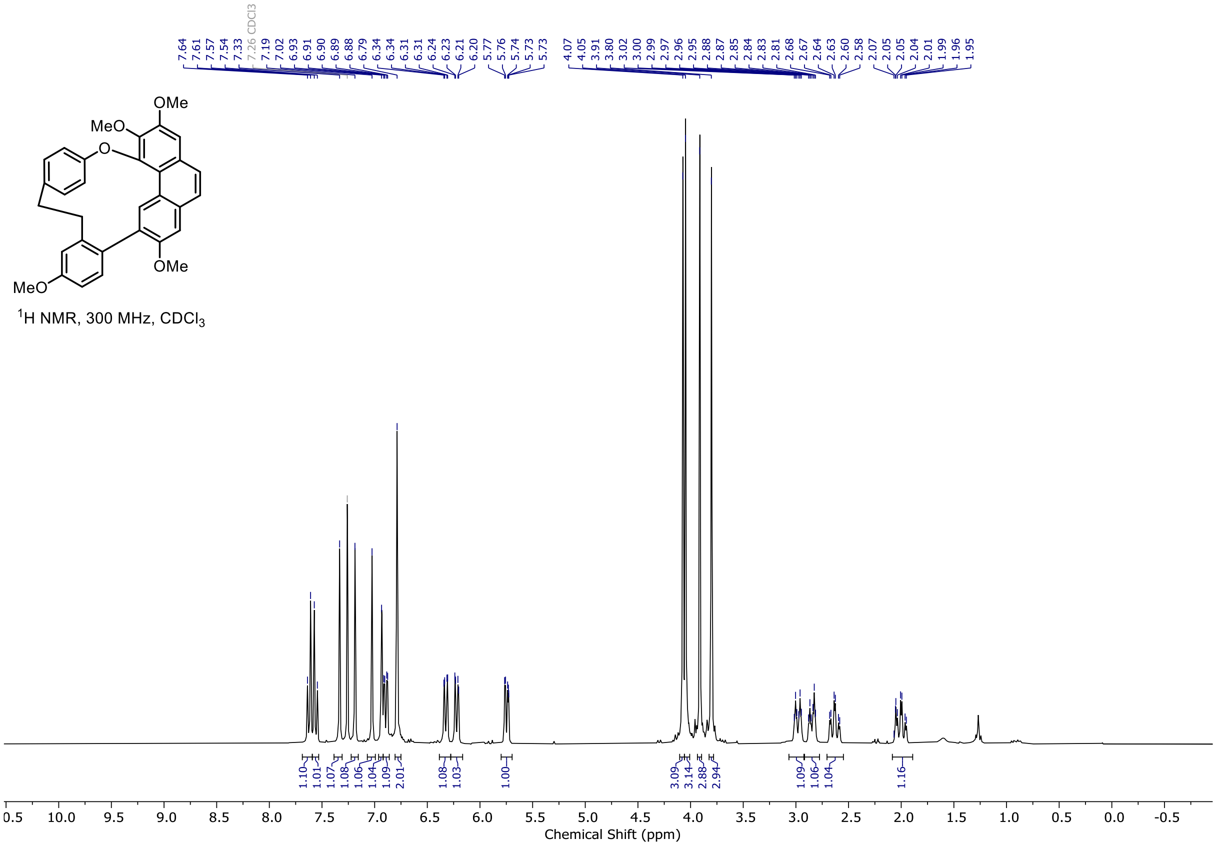

^{1}H NMR, 300 MHz, $CDCl_3$

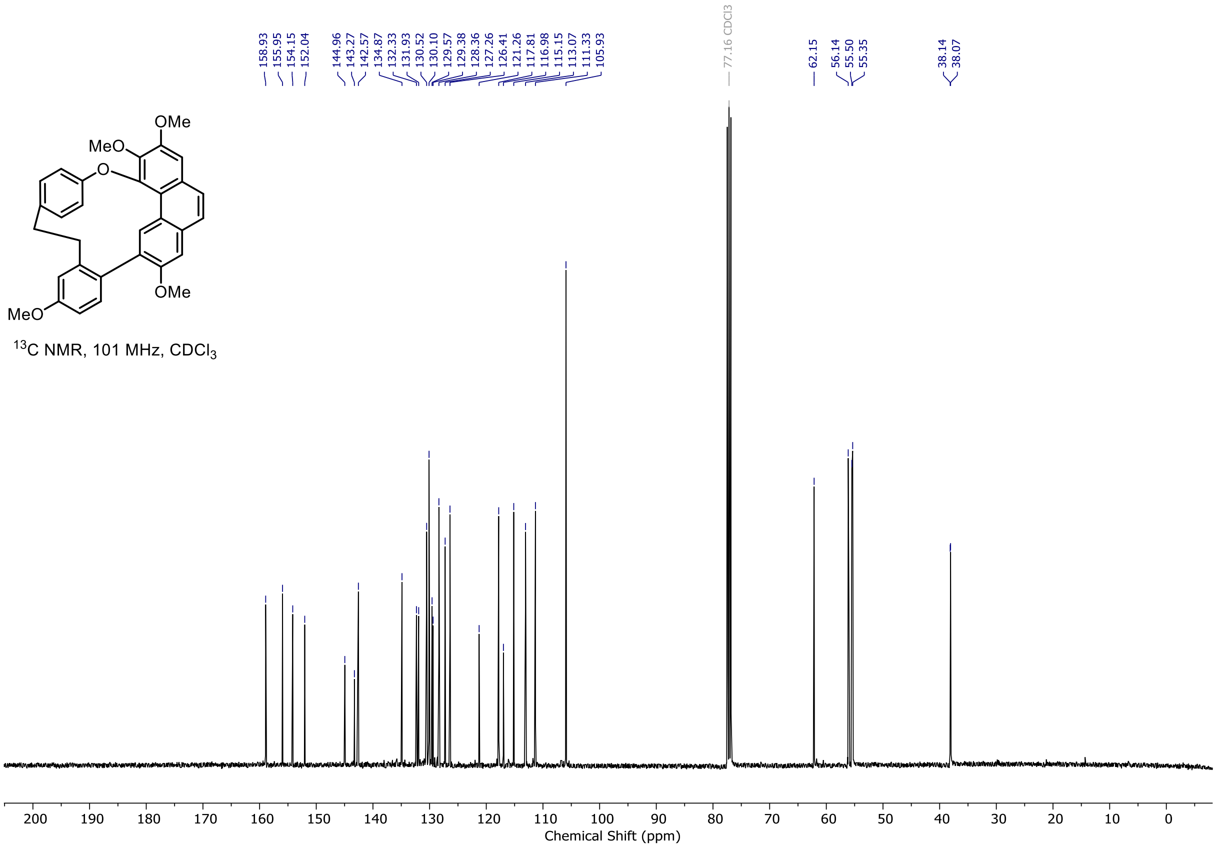

^{13}C NMR, 101 MHz, $CDCl_3$

Compound 1

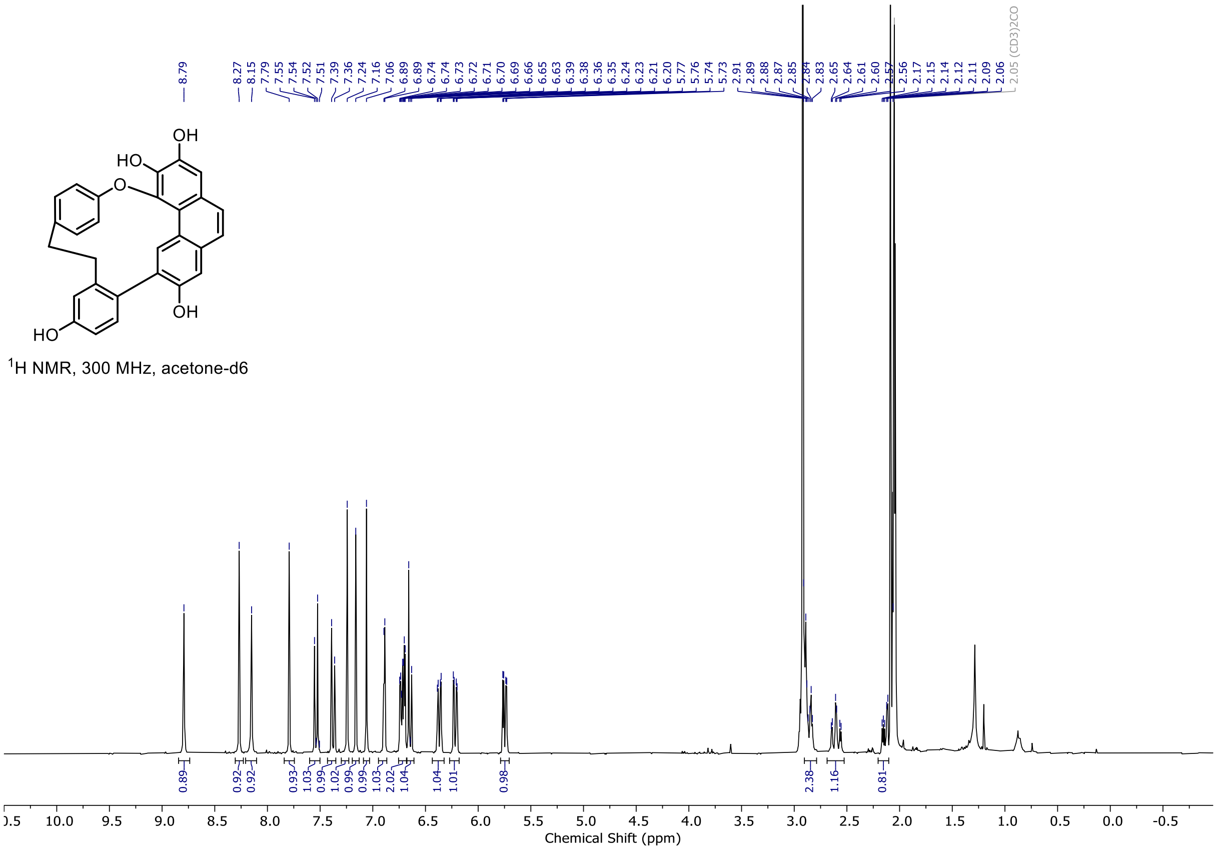

^{1}H NMR, 300 MHz, acetone-d6

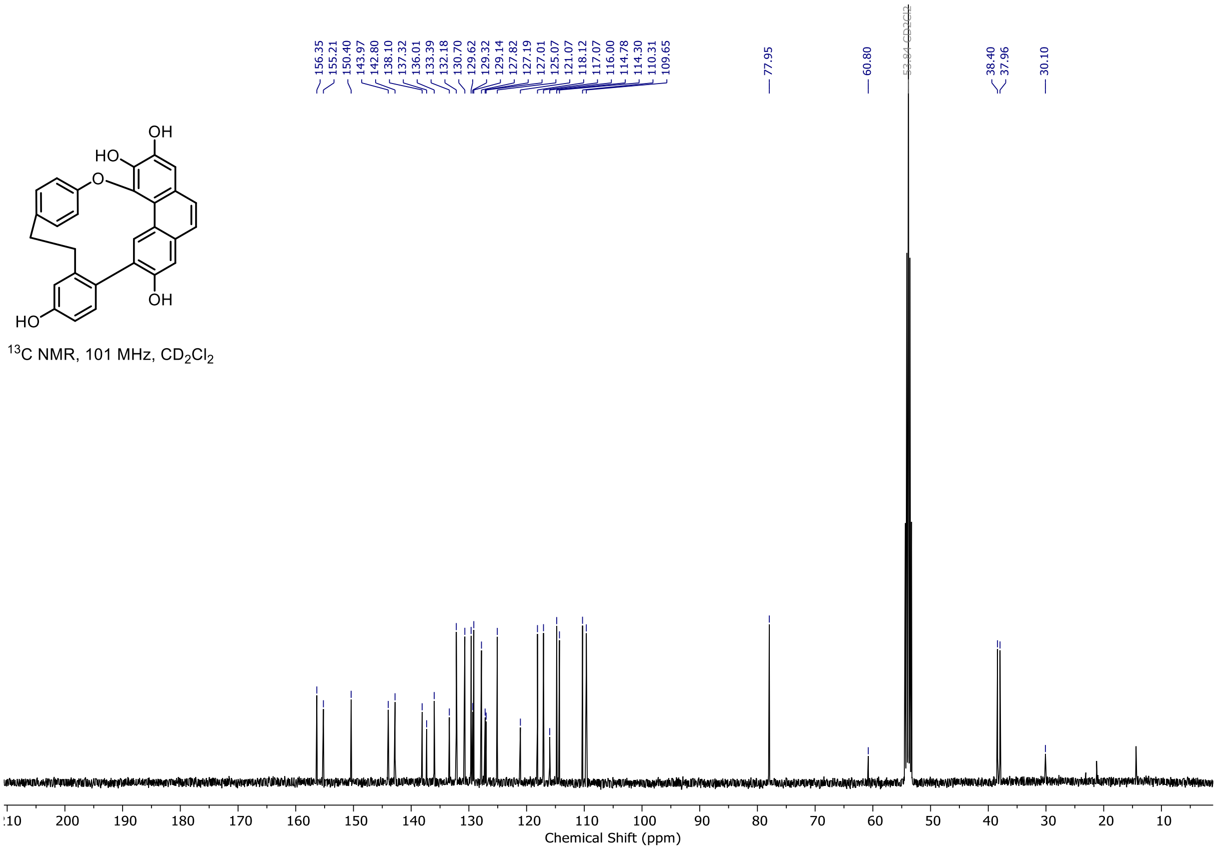

^{13}C NMR, 101 MHz, CD_2Cl_2

CD measurements

The separation of the constitutional enantiomers of **1** was carried out via semi-preparative HPLC with a chiral stationary phase (Daicel IA-3 SFC). Enantiomer A eluted first (red line), and enantiomer B eluted second (green line).

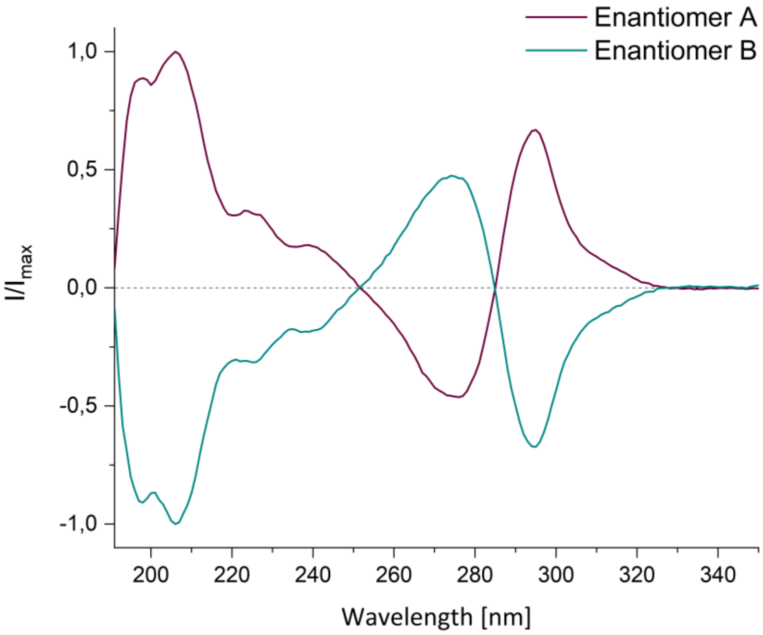

HPLC spectra for compound 1

Sample Information

Sample Name	: HoDoa-D27
Injection Volume	: 10
Data File	: HoDoa-D27_Hex-IPA_50-50_IA-3.lcd
Method File	: IA-3_Hex-IPA_50-50_25C_20m.lcm
Comment	: HoDoa-D27 in Hex-IPA 90-10<br>Hex-IPA 50-50, 20 min isocratic<br>1.0 mL/min, 298 K<br>IA-3, 4.6x150mm, 3µm
Level#	: 0

Chromatogram

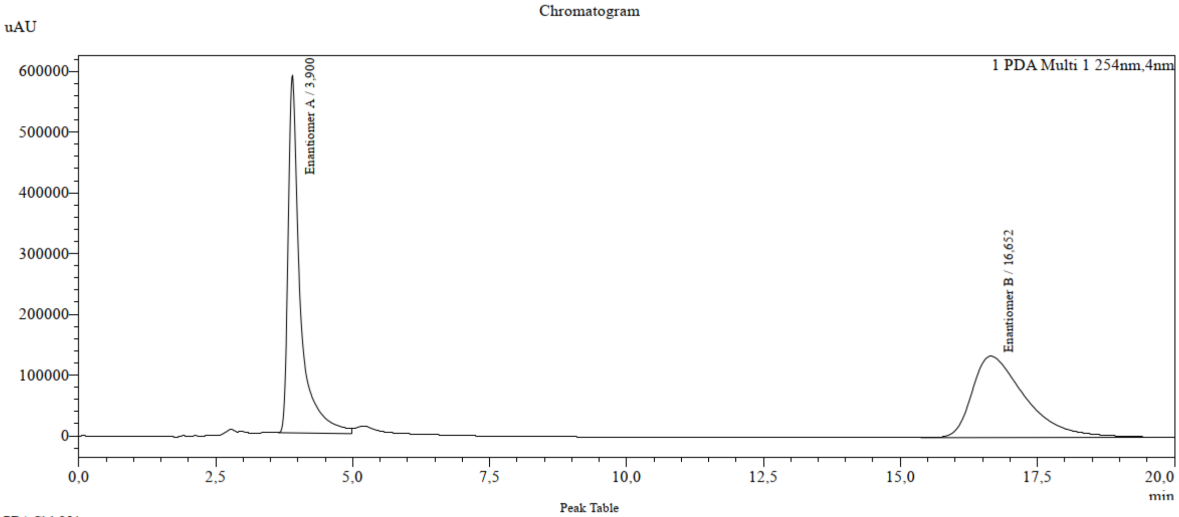

Peak Table

PDA Ch1 254nm

Peak#	Ret. Time	Area	Height	Area%
1	3,900	8980769	588170	49,785
2	16,652	9058433	133802	50,215
Total		18039202	721972	100,000

Crystallographic Supplement

Compound 1◦2MeOH◦KOAc

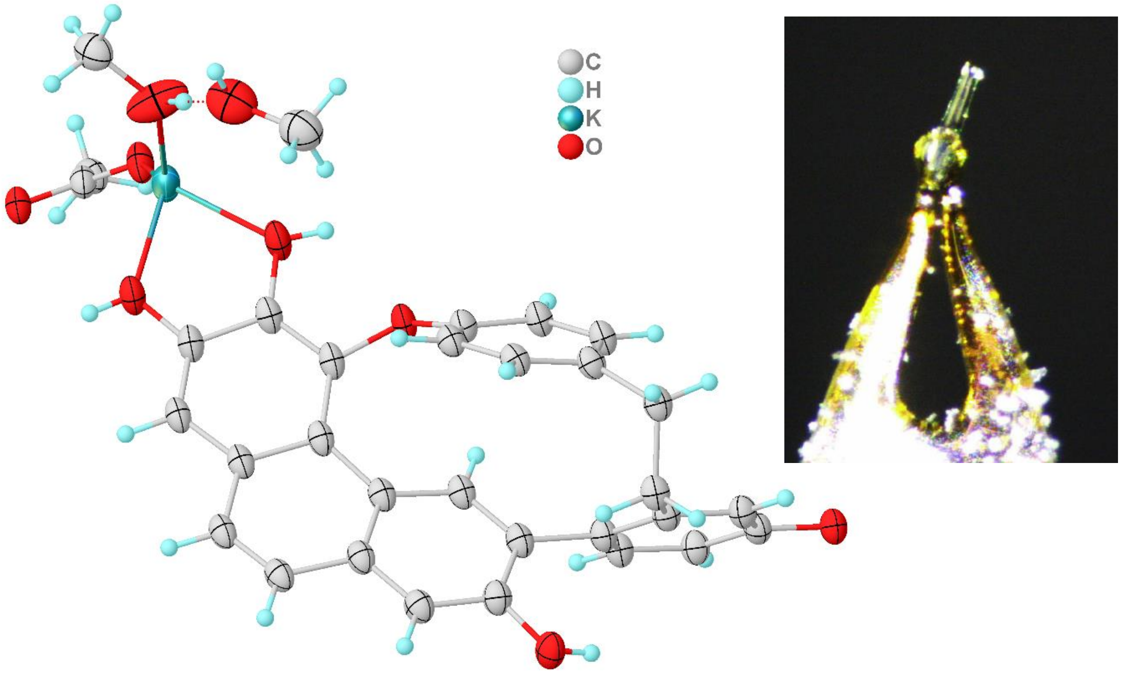

*Figure S 1: Full asymmetric unit of **1**◦2MeOH◦KOAc. Ellipsoids drawn at 30 % probability level. Single crystals were grown from methanol solution by slow evaporation.*

CCDC number	2283942
Empirical formula	$C_{62}H_{54}K_2O_{16}$
Formula weight	1133.25
Temperature [K]	100.00
Crystal system	monoclinic
Space group (number)	$P2_1/n$ (14)
a [Å]	15.1227(6)
b [Å]	11.2857(4)
c [Å]	15.5631(6)
α [°]	90
β [°]	101.861(3)
γ [°]	90
Volume [Å^3]	2599.45(17)
Z	2
ρ_{calc} [gcm^{-3}]	1.448
μ [mm^{-1}]	2.252
F(000)	1184
Crystal size [mm^3]	0.388×0.045×0.042
Crystal color	colorless
Crystal shape	needle
Radiation	CuK_α (λ=1.54178 Å)

2θ range [°]	7.42 to 159.12 (0.78 Å)
Index ranges	$-19 \le h \le 19$<br>$-13 \le k \le 14$<br>$-19 \le l \le 19$
Reflections collected	92531
Independent reflections	5632<br>R_{int} = 0.0595<br>R_{sigma} = 0.0221
Completeness to θ = 67.679°	100.0 %
Data / Restraints / Parameters	5632/5/398
Goodness-of-fit on F^2	1.033
Final R indexes [I≥2σ(I)]	R_1 = 0.0554<br>wR_2 = 0.1654
Final R indexes [all data]	R_1 = 0.0603<br>wR_2 = 0.1714
Largest peak/hole [eÅ^{-3}]	0.41/-0.88

Compound 11

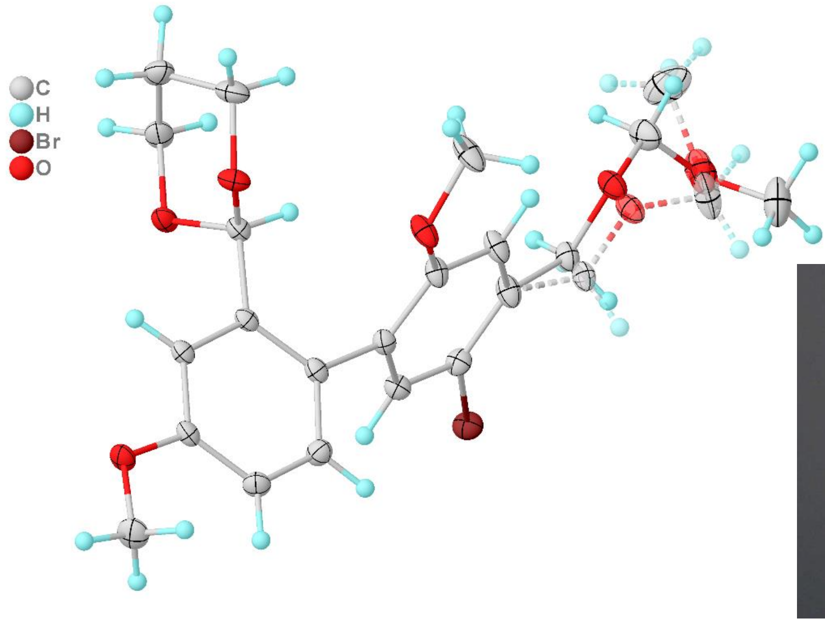

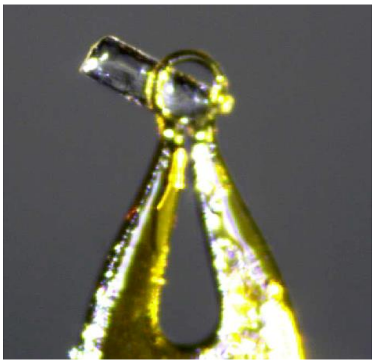

*Figure S 2: Full asymmetric unit of **11**. Ellipsoids drawn at 50% probability level. Minor (0.329) disorder part of the MOM protected alcohol depicted translucent with stippled bonds. Single crystals were obtained by evaporation of a solution in diethyl ether.*

CCDC number	2283943
Empirical formula	$C_{21}H_{25}BrO_6$
Formula weight	453.32
Temperature [K]	100.00
Crystal system	monoclinic
Space group (number)	$P2_1/c$ (14)
a [Å]	15.4017(14)
b [Å]	8.5022(8)
c [Å]	16.8527(13)
α [°]	90
β [°]	110.562(3)
γ [°]	90
Volume [Å^3]	2066.2(3)
Z	4
ρ_{calc} [gcm^{-3}]	1.457
μ [mm^{-1}]	2.023
F(000)	936
Crystal size [mm^3]	0.489×0.142×0.054
Crystal color	colorless
Crystal shape	plank

Radiation	MoK_α (λ=0.71073 Å)
2Θ range [°]	4.94 to 61.10 (0.70 Å)
Index ranges	$-21 \leq h \leq 22$ $-12 \leq k \leq 12$ $-24 \leq l \leq 24$
Reflections collected	53017
Independent reflections	6279 R_{int} = 0.0408 R_{sigma} = 0.0233
Completeness to Θ = 25.242°	99.7 %
Data / Restraints / Parameters	6279/18/285
Goodness-of-fit on F^2	1.113
Final R indexes [$I \geq 2\sigma(I)$]	R_1 = 0.0397 wR_2 = 0.0966
Final R indexes [all data]	R_1 = 0.0491 wR_2 = 0.1005
Largest peak/hole [eÅ^{-3}]	0.94/-0.57

Compound 16◦0.5 $CHCl_3$

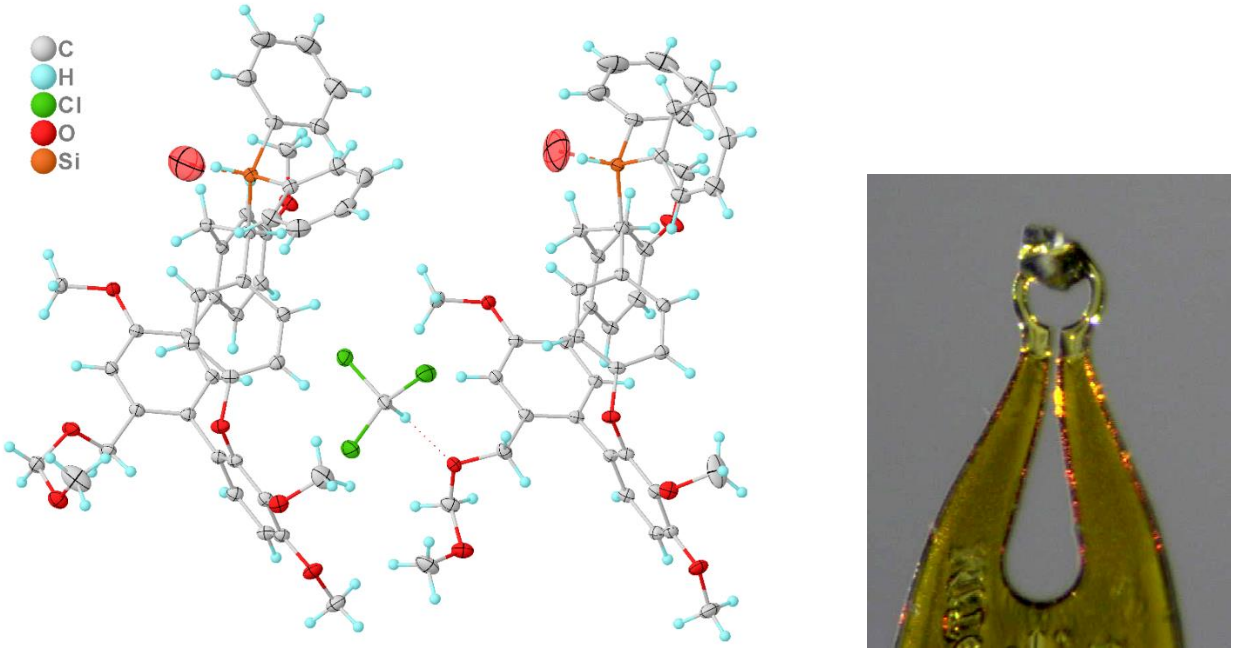

*Figure S 3: Full asymmetric unit (Z'=2) of **16**◦0.5 CHCl₃. The silanes were found to partially oxidize during crystallization under ambient conditions, which was modelled with partial oxygen positions instead of hydrides and are depicted as translucent ellipsoids with stippled bonds. Refined site occupancy factors for Si-O were 0.136(7) and 0.121(7). Ellipsoids drawn at 50% probability level. Single crystals were obtained by evaporation of a solution in chloroform.*

CCDC number	2283944
Empirical formula	$C_{91}H_{89}Cl_3O_{14}Si_2$
Formula weight	1569.15
Temperature [K]	100.00
Crystal system	triclinic
Space group (number)	$P\bar{1}$ (2)
a [Å]	14.0387(9)
b [Å]	15.0330(11)
c [Å]	20.1884(16)
α [°]	82.588(2)
β [°]	72.670(2)
γ [°]	80.451(2)
Volume [$Å^3$]	3996.5(5)
Z	2
ρ_{calc} [gcm^{-3}]	1.304
μ [mm^{-1}]	0.211
F(000)	1652
Crystal size [mm^3]	0.145×0.133×0.129
Crystal color	colorless
Crystal shape	block
Radiation	MoK_α (λ=0.71073 Å)

2Θ range [°]	3.62 to 59.53 (0.72 Å)
Index ranges	-19 ≤ h ≤ 19<br>-20 ≤ k ≤ 20<br>-28 ≤ l ≤ 28
Reflections collected	206544
Independent reflections	22521<br>R_{int} = 0.0353<br>R_{sigma} = 0.0182
Completeness to Θ = 25.242°	99.9 %
Data / Restraints / Parameters	22521/0/1022
Goodness-of-fit on F^2	1.020
Final R indexes [I≥2σ(I)]	R_1 = 0.0370<br>wR_2 = 0.0929
Final R indexes [all data]	R_1 = 0.0438<br>wR_2 = 0.0980
Largest peak/hole [$eÅ^{-3}$]	0.56/-0.45

Compound 19

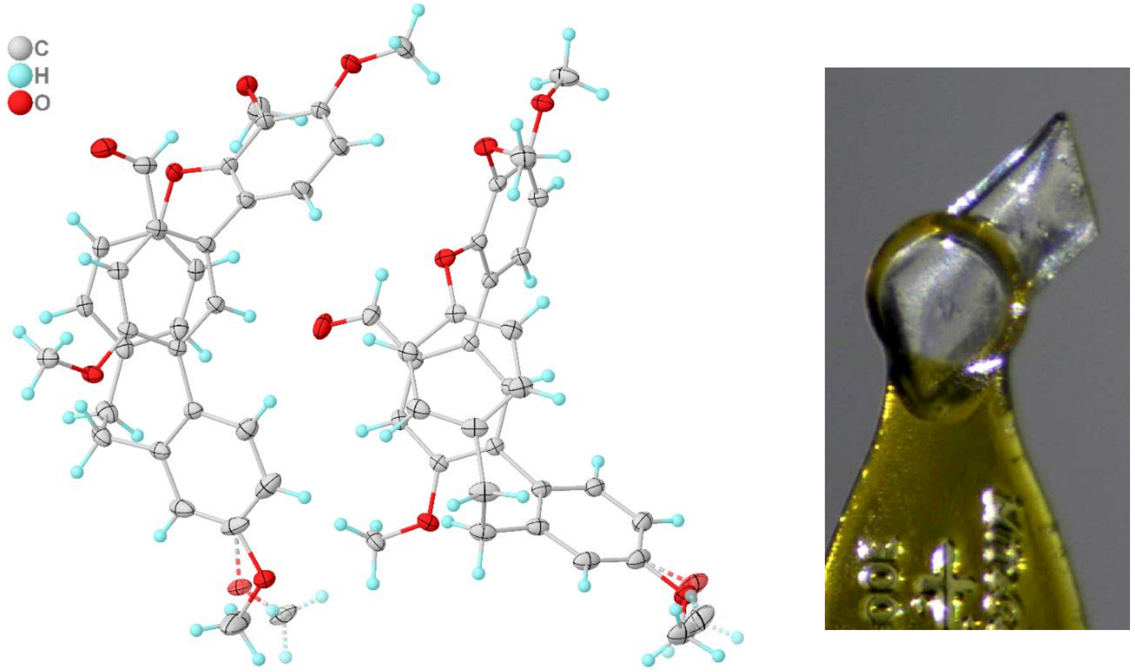

*Figure S 4: Full asymmetric unit (Z'=2) of **19**. Minor (0.362 and 0.375) disorder parts of the methoxy groups depicted translucent with stippled bonds. Ellipsoids drawn at 50% probability level. Single crystals were obtained by evaporation of a solution in ethyl acetate and hexane mixture. A cavity was treated with solvent mask, since attempts to model its content failed.*

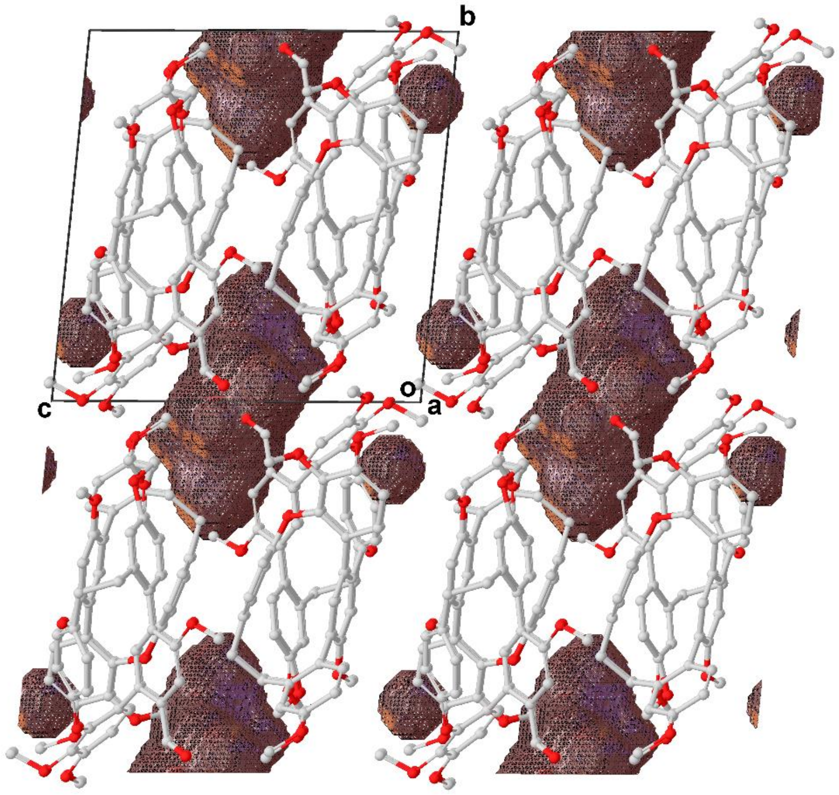

*Figure S 5: Packing diagram of **19** showing the masked volume. The cavity has a volume of 153.4 Å^3 and contains 46 electrons, which would be consistent with one molecule of hexane.*

CCDC number	2283945
Empirical formula	$C_{32.50}H_{31.50}O_6$
Formula weight	518.08
Temperature [K]	100.00
Crystal system	triclinic
Space group (number)	$P\overline{1}$ (2)
a [Å]	13.3408(10)
b [Å]	14.1636(13)
c [Å]	15.1985(15)
α [°]	94.918(3)
β [°]	112.732(2)
γ [°]	90.794(2)
Volume [Å^3]	2635.5(4)
Z	4
ρ_{calc} [gcm^{-3}]	1.306
μ [mm^{-1}]	0.089
F(000)	1098
Crystal size [mm^3]	0.825×0.253×0.108
Crystal color	colorless
Crystal shape	plate

Radiation	MoK_α (λ=0.71073 Å)
2Θ range [°]	3.90 to 65.34 (0.66 Å)
Index ranges	-20 ≤ h ≤ 20 -21 ≤ k ≤ 21 -23 ≤ l ≤ 22
Reflections collected	169563
Independent reflections	18369 R_{int} = 0.0366 R_{sigma} = 0.0223
Completeness to Θ = 25.242°	99.9 %
Data / Restraints / Parameters	18369/6/715
Goodness-of-fit on F^2	1.020
Final *R* indexes [I≥2σ(I)]	R_1 = 0.0451 wR_2 = 0.1180
Final *R* indexes [all data]	R_1 = 0.0576 wR_2 = 0.1281
Largest peak/hole [eÅ^{-3}]	0.40/-0.46

Compound 22

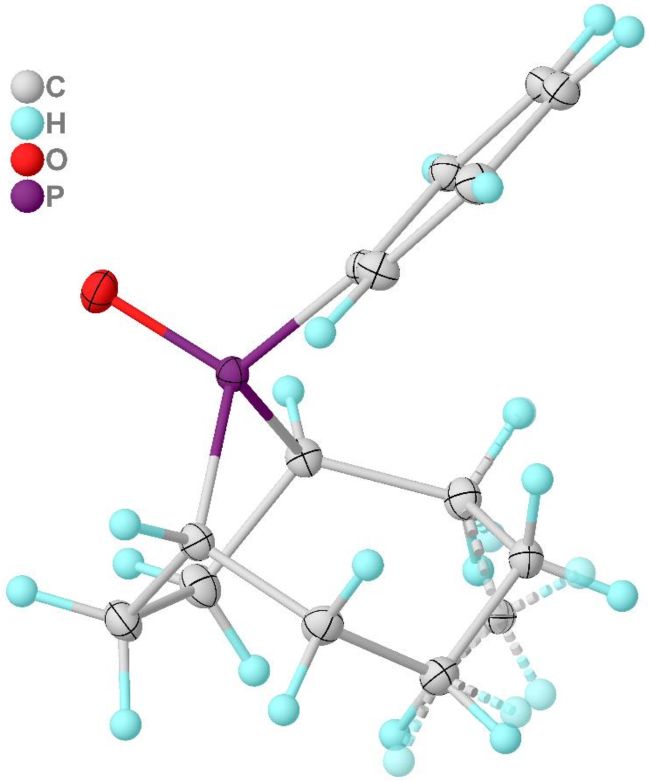

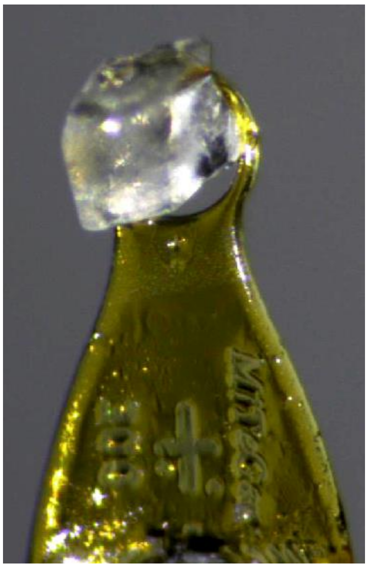

*Figure S 6: Full asymmetric unit of **22**. Ellipsoids drawn at 50% probability level. Minor (0. 307) disorder part depicted translucent with stippled bonds. Single crystals were obtained by evaporation of a solution in dichloromethane.*

CCDC number	2283946
Empirical formula	$C_{14}H_{19}OP$
Formula weight	234.26
Temperature [K]	100.00
Crystal system	Monoclinic
Space group (number)	$P2_1/n$ (14)
a [Å]	7.4756(3)
b [Å]	13.3050(5)
c [Å]	11.9637(4)
α [°]	90
β [°]	97.5720(10)
γ [°]	90
Volume [Å^3]	1179.57(8)
Z	4
ρ_{calc} [gcm^{-3}]	1.319
μ [mm^{-1}]	0.209
F(000)	504
Crystal size [mm^3]	0.389×0.341×0.245
Crystal color	Colorless
Crystal shape	Block

Radiation	MoK_α (λ=0.71073 Å)
2Θ range [°]	4.60 to 71.02 (0.61 Å)
Index ranges	-11 ≤ h ≤ 11<br>-21 ≤ k ≤ 20<br>-19 ≤ l ≤ 19
Reflections collected	41289
Independent reflections	5085<br>R_{int} = 0.0238<br>R_{sigma} = 0.0119
Completeness to Θ = 25.242°	100.0 %
Data / Restraints / Parameters	5085/6/155
Goodness-of-fit on F^2	1.089
Final R indexes [I≥2σ(I)]	R_1 = 0.0260<br>wR_2 = 0.0756
Final R indexes [all data]	R_1 = 0.0276<br>wR_2 = 0.0767
Largest peak/hole [eÅ^{-3}]	0.48/-0.32

Compound 24

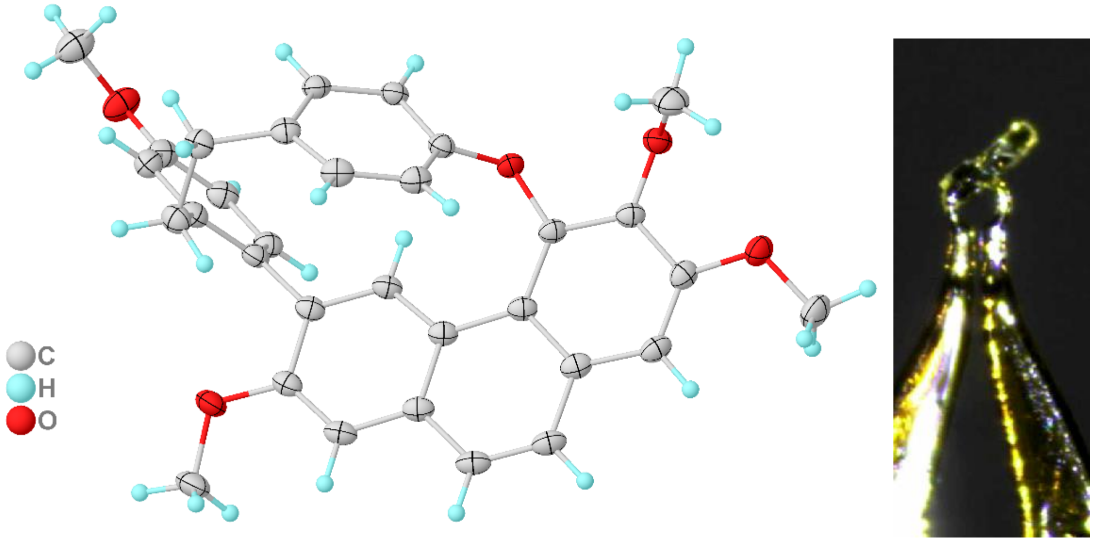

Figure S 7: Full asymmetric unit of 24. Ellipsoids drawn at 50% probability level. Crystals were obtained by evaporation of a solution in ethyl acetate and hexane mixture. The material was found to inherently form multi-crystals; in the used specimen three minor domains were identified and the data was integrated as a twin, with the major domains' ratio at ca 90%. The final refinement was done against the detwinned hklf4 data, since the data was found to be superior to the combined hklf5.

CCDC number	2283947
Empirical formula	$C_{32}H_{28}O_5$
Formula weight	492.54
Temperature [K]	100.00
Crystal system	monoclinic
Space group (number)	$P2_1/c$ (14)
a [Å]	11.1020(11)
b [Å]	19.896(2)
c [Å]	11.0378(11)
α [°]	90
β [°]	90.587(4)
γ [°]	90
Volume [Å^3]	2437.9(4)
Z	4
ρ_{calc} [gcm^{-3}]	1.342
μ [mm^{-1}]	0.090
F(000)	1040
Crystal size [mm^3]	0.251×0.073×0.051
Crystal color	colorless
Crystal shape	needle

Radiation	MoK_α (λ=0.71073 Å)
2Θ range [°]	4.20 to 59.16 (0.72 Å)
Index ranges	-15 ≤ h ≤ 14 -27 ≤ k ≤ 27 -15 ≤ l ≤ 15
Reflections collected	47807
Independent reflections	6826 R_{int} = 0.0383 R_{sigma} = 0.0268
Completeness to Θ = 25.242°	99.9 %
Data / Restraints / Parameters	6826/0/338
Goodness-of-fit on F^2	1.089
Final R indexes [I≥2σ(I)]	R_1 = 0.0525 wR_2 = 0.1313
Final R indexes [all data]	R_1 = 0.0752 wR_2 = 0.1480
Largest peak/hole [eÅ^{-3}]	1.29/-0.26
